# Enriched stable ^204^Pb as tracer at ultra-low levels in clinical investigations

**DOI:** 10.1007/s00216-022-04311-0

**Published:** 2022-09-22

**Authors:** Johanna Irrgeher, Thomas Berger, Anastassiya Tchaikovsky, Cornelius Tschegg, Ghazaleh Gouya, Peter Lechner, Anika Retzmann, Christine Opper, Christa Firbas, Michael Freissmuth, Kerstin Peschel-Credner, Karolina Anderle, Claudia Meisslitzer, Michael Wolzt, Thomas Prohaska

**Affiliations:** 1 Department of General, Analytical and Physical Chemistry, Montanuniversität Leoben, Franz-Josef-Straße 18, 8700 Leoben, Austria; 2 Glock Health Science and Research GmbH, Hausfeldstraße 17, 2232 Deutsch-Wagram, Austria; 3 Gouya Insights, Elisabethstrasse 22/12, 1010 Vienna, Austria; 4 LGS-INSIGHTS GmbH, Elisabethstrasse 22/12, 1010 Vienna, Austria; 5 TB Unterfrauner GmbH, Umseerstraße 39, 3040 Neulengbach, Austria; 6 Department of Clinical Pharmacology, Medical University of Vienna, Währinger Gürtel 18-20, 1090 Vienna, Austria; 7 Institute of Pharmacology and the Gaston H. Glock Research Laboratories for Exploratory Drug Development, Center of Physiology and Pharmacology, Medical University of Vienna, Währingerstrasse 13a, Vienna, Austria

**Keywords:** Blood, Zeolite, Lead, Clinoptilolite, Isotope pattern deconvolution, ICP-MS

## Abstract

The potential of enriched Pb (^204^Pb) was assessed to monitor pathways of trace levels of Pb in the pg range within the human body via isotope pattern variation in situations where natural lead cannot be used as a tracer due to regulatory limitations. Isotope ratio measurements were accomplished by means of (multi-collector) inductively coupled plasma mass spectrometry including a comparison of single and multi-collector ICP-MS for low-level ^204^Pb assessment. Isotopic pattern results from a blend of a large quantity of the element with a natural isotopic composition and an enriched stable isotope at orders of magnitude lower levels pose a nontrivial analytical problem. Isotope pattern deconvolution was successfully applied as mathematical tool based on multiple linear regressions. The method allowed for deconvolving the isotope pattern from measured isotope ratios without knowing the quantities of different isotope sources incorporated and mixed into the sample at levels of < 1 pg ^204^Pb/g blood. The objective of this manuscript is to evaluate and summarize the analytical aspects for Pb isotope pattern deconvolution based on the results of a clinical trial, where a ^204^Pb-enriched isotope tracer was applied to investigate the bioavailability of orally applied Pb along with purified clinoptilolite tuff as potential supplement. This unique approach allows to reduce tracer amounts to harmless levels to human health, which are in accordance with the legal regulative to study enrichment levels of < 0.01% in human blood.

**Graphical abstract:**

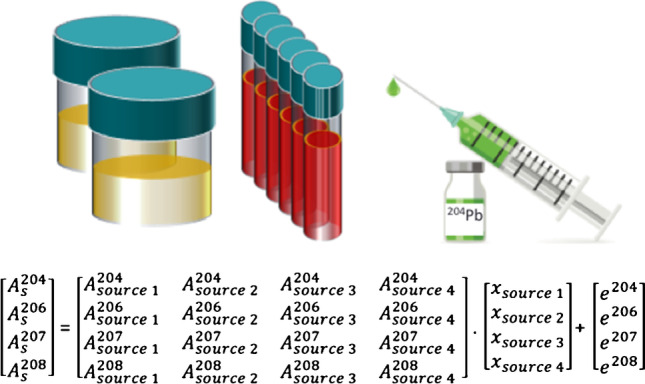

**Supplementary Information:**

The online version contains supplementary material available at 10.1007/s00216-022-04311-0.

## Introduction

Enriched stable isotopes provide an outstanding tool for tracer studies in living systems. In the past, radioactive isotopes were used for addressing questions related to the biology of metals and trace metals [1–3]. Radioisotopes can be used at ultra-low levels to trace the biological fate of elements. However, possible side effects caused by the radioactivity had to be taken into account [4]. Enriched spikes of naturally occurring non-radioactive isotopes have experienced increasing interest as monitors of metabolic processes in life sciences [5, 6]. Since these spikes are unique markers, they allow to monitor elemental uptake, distribution, deposition, or mobilization in living organism and to study the fate of materials applied for medical purposes. The advantages and challenges of introducing an isotopically enriched tracer into an organism are described in detail elsewhere [7–9].

A prerequisite of tracer-based studies is to minimize or preclude the possible negative impact (i.e. the toxicity) of tracers on the organism (in particular on human subjects). Thus, only very low molar amounts of tracer material ideally below any limits of toxicity are to be introduced into biological systems. The resulting challenge is to decrypt isotopic mixtures composed of the enriched stable tracer isotope and natural-abundance levels of the investigated element. Isotope pattern deconvolution (IPD) has been proven as method of choice in this context [8].

Any exposure to lead is considered to cause adverse health effects [10, 11]. Blood lead levels as low as 5 µg dL^−1^ (or about 50 ng g^−1^) are associated with increased risk of cardiovascular disease mortality [12]. Subtle neuropsychological impairment and headaches appear at blood lead levels of 25 µg dL^−1^ (or about 250 ng g^−1^). Blood lead levels exceeding 100 µg dL^−1^ (or about 1,000 ng g^−1^) are associated with an increased intracranial hypertension, encephalopathy with delirium and peripheral motor neuropathy [13]. Treatment of acute lead poisoning is based on administration of chelating agents, which reduce lead blood levels by forming chelator-lead complexes that are excreted in urine [14]. However, these drugs have serious side effects and require repeated courses of therapy. Thus, they are recommended only for patients with blood lead levels of greater than 45 µg dL^−1^ (450 ng g^−1^) [13, 15]. As a consequence, elimination of the source of lead exposure recommended as the single most effective way to prevent lead-induced adverse health effects [11]. The toxico-kinetics of lead are outlined in detail in Samekova et al. [16].

Contaminated water and food are the primary sources of lead exposure for the general population [17]. Lead in drinking water originates from the environment (natural and industrial sources) and/or lead containing water pipes [18]. Crops, fruits and vegetables can accumulate lead from (contaminated) soil in which they grow [10].

Clinoptilolite, a natural non-toxic mineral of the zeolite group, can be administered as preventive measure to bind lead with high selectivity and efficiency [19]. Due to its specific crystal structure and negative framework-charge, clinoptilolite acts as an active sorbent and ion-exchanger for free lead ions. In mice, which were exposed to elevated levels of lead (i.e. 50 mmol L^−1^) in their drinking water for 90 days, the concomitant administration of clinoptilolite reduced the accumulation of lead in the kidney and bones by about 90% and 70%, respectively [20]. These and related data [20–32] imply that clinoptilolite is potentially useful for reducing the amount of bioavailable heavy metals in people. It is worth pointing out, though, that people are exposed to submicromolar rather than millimolar levels of lead in their drinking water.

Accordingly, a clinical study was designed in accordance with Good Clinical Practice, the ISO 14155 [33] and the European Union Directive 98/63 EG [34] recommending an upper threshold of lead in drinking water of 10 µg L^−1^. The study aimed at the investigation of enteral lead uptake in healthy humans with concomitant oral intake of purified clinoptilolite-tuff (approved by the Food and Drug Administration for use as a dietary supplement with the brand name G-PUR®) [16]. Based on literature, the expected amounts of ^204^Pb tracer to be administered in blood and urine of human subjects required elaborated analytical measurement procedures and validation. This included the comprehensive assessment of blanks and of consumables used in the clinical study, sample preparation and the evaluation and comparison of methods based on single collector and multi collector inductively coupled plasma mass spectrometry for isotope ratio measurements. Isotope pattern deconvolution had to be adapted to allow for detection of ultra-low level enriched stable isotope tracers and for its successful application to a large sample set from a clinical trial.

## Materials and methods^1^

Preparatory laboratory work was performed in a class 100,000 clean room. Type I reagent-grade water (18 MΩ cm) (F + L GmbH, Vienna, Austria) was further purified by sub-boiling distillation (Milestone-MLS GmbH, Leutkirch, Germany). Analytical reagent-grade nitric acid (*w* = 65%, Merck-Millipore) was purified by double sub-boiling using a DST-1000 sub-boiling distillation system (AHF Analysentechnik, Tübingen, Germany). Polyethylene (PE) flasks, tubes and pipette tips (VWR International, Radnor, USA), as well as perfluoroalkoxy (PFA) screw cap vials (Savillex, Eden Prairie, USA) were pre-cleaned in a two-stage washing procedure using nitric acid (*w* = 10% and 1% respectively).

Certified reference material NIST SRM 981 (high purity lead metal, NIST, Gaithersburg, US) was used for Pb isotopic analysis to optimize instrument parameters as well as for calibration of instrumental isotopic fractionation (IIF). Solutions of this standard were prepared gravimetrically in nitric acid (*w* = 2%) and diluted to final mass fraction of *w* = 50–100 ng g^−1^ as isotopic reference for standard-sample bracketing (SSB) to correct for IIF.

### Blank assessment of materials used in clinical study and in lab

#### Mineral water (Vöslauer Still®): tracer vector

Commercially available 500 mL bottles (*n* = 5) of Vöslauer Still® (Vöslauer, Vienna, Austria) water were opened using a commercially available, pre-cleaned crown cork opener, and 10 mL of water was transferred directly into pre-cleaned test tubes. For the measurement using ICP-MS, the solutions were acidified with HNO_3_ (*w* = 65%, 2 × subboiled) in order to obtain a HNO_3_ solution of *w* = 2%. A triplicate determination was carried out for each bottle.

#### Crown corks for re-sealing of bottles

Crown corks were covered with reagent grade type I H_2_O in pre-cleaned plastic containers and leached out overnight. After leaching, the solution was acidified for the measurement.

#### Medical test tubes and needles

Different commercially available test tubes dedicated to sampling of blood during the clinical trial were leached with 4 mL HNO_3_ (*w* = 2%) (prepared from HNO_3_ (*w* = 65%, 2 × subboiled and ultrapure water, 1 × subboiled) for 24 h. This solution was measured directly with ICP-MS. The necessary sample preparation and leaching steps were adapted according to the material. Needles were placed in water overnight or leached directly by pressing the solution through using pre-cleaned syringes. The tube types tested included routinely used Vacuette tubes (Greiner Bio-One, purple K3EDTA (Ref. 454217); green (lithium heparin) (Ref. 454217); green (lithium heparin) Ref. 455084; blue (9NC Coagulation sodium citrate 3.2%) (Ref. 454332); grey (FE Sodium Fluoride/K3EDTA) (Ref. 454033); yellow (Z Urine No Additive) (Ref. 455028)); Safety Blood Collection Greiner Bio-One; VASOFIX SAFETY PUR 18G (Braun); and BD Venflon Pro Safety 20G (Beckton Dickinson Infusion Therapy).

### Lead isotope tracer

The 2.5 μg dose of ^204^Pb was prepared by solving 11.74 mg of 99.94% atom-enriched metal ^204^Pb in 5.0 mL of *w* = 15% nitric acid and further diluting it with ultrapure water to a final lead mass concentration of 2681 μg L^−1^ for spiking bottled water.

### Experimental design using certified reference materials of blood

Blood was the primary target matrix in the clinical study. Thus, preliminary laboratory spiking experiments using matrix-matched blood certified reference materials and the ^204^Pb tracer were carried out. Therefore, we used blood certified reference material quantities, which were in accordance with the planned human study. This allowed to characterize the expected degree of enrichment and develop the analytical measurement procedure accordingly.

Three different blood certified reference materials were available for the experiments: BCR-634 (*γ*(Pb) = 46 μg L^−1^), BCR-635 (*γ*(Pb) = 210 μg L^−1^) and BCR-636 (*γ*(Pb) = 520 μg L^−1^). The lead isotope tracer solution of the dissolved ^204^Pb Spike-Tracer was used for spiking after further dilution to *γ* (^204^Pb) = 1 µg L^−1^. For the spike experiment, 10 mixtures of blood and tracer and 10 blanks with microwave digestion were produced and, after dilution, measured using ICP-MS. BCR-636 (*γ*(Pb) = 520 μg L^−1^) was chosen for the spike tests. The high Pb concentration allowed a large number of parallel experiments from the same starting material. Relative enrichment levels in the range from 0.01 to 0.50% were analysed, beside blank digests. The levels were chosen accordingly in order to determine the limitations of the method (i.e. ^204^Pb minimum amount that is necessary to monitor a significant change in the Pb isotopic composition).

A final volume of 1 mL was selected for each mixture prepared for digestion. A volume of the diluted tracer solution (*γ*(^204^Pb) = 1 µg L^−1^, *V* ranging from 10 to 520 µL) was added to a volume of 200 μL blood (BCR-636) (*m*(Pb_nat_) = 104 ng) and then filled up to 1000 μL with HNO_3_ (*w* = 2%). After a reaction time of 10 min, 4 mL HNO_3_ (*w* = 65%, 2 × subb.) and 0.5 mL H_2_O_2_ were added as digestion reagents. After the addition of HNO_3_, the solution was shaken, and the cap of the reagent tube was opened 2–3 times in order to allow gases to be released to reduce the pressure in the vessel.

Subsequently, mixtures of BCR-636 and ^204^Pb-traver were digested by means of microwave pressure digestion (Multiwave Pro, Anton Paar GmbH, Graz, Austria) in a 48MF50 rotor, using pre-cleaned vessels. The microwave program was ramped up at 1400 W for 15 min and then held at power for 20 min before being cooled for 15 min. The Pb isotope ratio measurements of mixtures of BCR-636 and ^204^Pb tracer were performed using multi-collector inductively coupled plasma mass spectrometry (MC ICP-MS) on a Nu Plasma HR (NP048) (Nu Instruments, Wrexham, UK). See the “Measurement procedures based on ICP-MS” section for details on Pb isotope ratio measurements by MC ICP-MS.

### Clinical trial

This trial was conducted as a medical device clinical trial according to the regulatory provisions in Austria. Glock Health, Science and Research GmbH acted as sponsor of this single centre study. The study protocol was approved by the Ethics Committee of the Medical University of Vienna (no. 1285/2019) and the Austrian Federal Office for Safety in Health Care (BASG) and is registered at clinicaltrials.gov (NCT04138693). This study was conducted at the Department of Clinical Pharmacology at the Medical University of Vienna in accordance with the Declaration of Helsinki. All study participants provided written informed consent before any study-specific procedures were performed. The study was initiated on 28th of August 2019 and the clinical phase completed on the 12th of February 2020. The study followed a randomized, placebo controlled, double-blind, parallel-group design. Male or female healthy volunteers aged 18–45 years with a body mass index (BMI) of 19–27 kg/m^2^ for males and 17–25 kg/m^2^ for females were eligible for participation. Main inclusion criteria were whole blood lead concentration < 40 μg/L as measured by graphite furnace atomic absorption spectroscopy, serum ferritin concentration within the sex-specific normal range and 1 month of stable eating habits. Key exclusion criteria were pregnancy or breastfeeding in women, regular use of medication or iron supplements in the previous 2 months, any relevant organ disease, especially gastrointestinal pathology, recent diarrhoea, diabetes, symptomatic food allergies or aluminium and/or silicon hypersensitivity. Participants were excluded, if they showed any clinically relevant laboratory abnormalities at screening, or alcohol, nicotine or drug abuse.

The investigational medical device (IMD) investigated in this study consisted of purified clinoptilolite-tuff (product name is G-PUR®). The raw material of the product originated from a large quarry in Eastern Slovakia and was refined with a technically sophisticated and fully quality-controlled production process. The patented purification process results in removal of natural bioavailable impurities to meet highest safety standards and regulatory requirements. For details about the clinoptilolite-tuff characteristics and source, see [36, 37].

The major component of the product is clinoptilolite, a natural non-toxic mineral of the zeolite group that due to its specific crystal structure and negative framework-charge, and acts as an active sorbent and ion-exchanger, capable of binding lead (among other heavy metals) with high selectivity and efficacy [19]. When orally ingested, the IMD remains inside the digestive tract and is eliminated via the stool. As the IMD is stable even in an acid pH environment, there is no major degradation of the structure.

In short, in this clinical investigation, healthy volunteers received 250 mL drinking water spiked with stable lead isotope ^204^Pb in a concentration of 10 µg L^−1^; no harm to the trial subjects was expected with the dose applied as kept below the Austrian drinking water regulation [38]. Overall, 42 subjects were enrolled and randomized in three groups, who received in addition to ^204^Pb spiked drinking water, 4.0 g G-PUR®, 2.0 g G-PUR® and placebo (no addition to water) (note that the numbers of subjects follow the numbers of recruitment; subjects not meeting the requirements of the trial, e.g. due to elevated Pb levels in blood, were excluded in the study). Blood samples for assessment of blood lead levels and the lead isotopes were taken at baseline (*t* = 0 h) and at specific timepoints of 4 h, 8 h, 12 h, 24 h, 48 h and 192 h after tracer administration. Urine was sampled at baseline and during the first 24 h after tracer administration. The clinical trial was carried out in two stages with 6 subjects in stage 1 sampled at more times as compared to stage 2 (36 subjects). Details about the clinical trial can be found in Samekova et al. [16].

#### Preparation of ^204^Pb enriched water for drinking

As the final dilution of ^204^Pb in mineral water was discovered to be only semi-stable (precipitation of PbCO_3_ and other insoluble Pb compounds may occur over time), the final dilution step was performed on-site, adding 925 μL of spiking solution to the 250 mL “Vöslauer Still®” glass bottles resulting in a final concentration of 10.0 μg L^−1^. After an overnight stay at the study site, all subjects received 2.5 μg ^204^Pb in 250 mL of mineral water (spiked 250 mL “Vöslauer Still®” in glass bottles). Subjects drank the bottle content using a straw; the exact amount of fluid/^204^Pb intake was assessed by weighing the spiked “Vöslauer Still®” glass bottles (straw included) before and after drinking. The concentration of ^204^Pb administered to the subjects did not exceed the maximum levels listed in the drinking water ordinance for Pb concentration [38]. The same applied for the nitrate concentration and pH of the final solution (0.67 mg L^−1^ nitrate, pH > 6.5 due to the buffering capacity of the mineral water).

### Sample preparation (clinical trial)

#### Blood sample digestion

Blood samples were pre-digested at the study site. 3.5 mL HNO_3_ (*w* = 65%*,* 1 × subb.) was added to 500 μL blood. The solutions were transferred under controlled conditions from the study site to the laboratories of Montanuniversität Leoben for further processing. Prior to digestion of the samples, purification digestion runs with HNO_3_ were performed. 0.5 mL H_2_O_2_ was added to pre-digested blood samples (500 μL blood + 3500 μL HNO_3_). After a reaction time of 10 min, the digestions of blood samples were performed by microwave pressure digestion (see details above). Samples were further diluted using ultrapure water or diluted nitric acid.

#### Urine sample digestion

Urine samples were stabilized at the study site by adding HNO_3_ (6 mol L^−1^) depending on the urine volume sampled aiming for *w* (HNO_3_) = 3–7%. The solutions were transferred from the study site to the laboratories of Montanuniversität Leoben under controlled conditions for further processing. Acidified urine samples were digested using 3.5 mL HNO_3_ (*w* = 65%, 2 × subb.) and 0.5 mL H_2_O_2_. The digestion of urine samples was performed by microwave pressure digestion (see details above). Samples were further diluted using ultrapure water or diluted nitric acid. Further treatment was done following the same procedures as for blood samples after digestion.

#### Blood sample preparation of real samples for MC ICP-MS measurements

Based on the results of the quantification by ICP-QMS of real blood samples and the expected sensitivity of the MC ICP-MS method, 4 mL of selected blood sample digest (subject-01 and subject-02) was evaporated to almost dryness on a hot-plate at 120 °C (note: the set temperature *T* does not equal *T* in the vessel; *T* was kept below boiling point of the sample solution to avoid boiling retardation) in 2 × pre-cleaned PFA vessels. This way, the samples reached a total amount of Pb ranging from 1 to 2 ng to allow measurements by MC ICP-MS using Faraday cups as detectors. The evaporated samples were re-dissolved in 1 mL HNO_3_ (*w* = 2%) for the MC ICP-MS measurement.

All seven blood digests (7 time points) of subject-01 and subject-02 were measured by MC ICP-MS.  Due to the low Pb levels present in the blood samples, this pre-concentration step was required, leading to increased matrix load during the measurement. The matrix caused the sample introduction system (Aridus II, Teledyne Cetac, Omaha, USA) to clog, eventually resulting in a total loss of signal. Thus, measurements of the entire sample batch were carried out using ICP-QMS and introducing a wet aerosol.

### Measurement procedures based on ICP-MS

Elemental analysis was performed on selected samples for quantification of analyte and matrix elements for validation purposes. Details are provided in the Supporting information SI Elemental analysis by ICP-MS.

Pb isotopic compositions of mixtures of BCR-636 and ^204^Pb tracer and blood of selected subjects were analysed to compare the performance of ICP-QMS to MC ICP-MS. ICP-QMS and MC ICP-MS were compared to determine the optimum measurement protocol for Pb isotope ratio measurements of low-level Pb in a blood matrix. The two setups available differ in their performance regarding Pb analysis primarily by sensitivity, with the ICP-QMS system being more sensitive than MC ICP-MS when Faraday cups are being used as detectors. In contrast, MC ICP-MS stands out because of its high-precision capabilities, given that a decent amount of analyte is present.

A single collector ICP-QMS instrument (NexION 2000, PerkinElmer, Ontario, Canada) equipped with a FAST introduction system and a multi collector ICP-MS (Nu Plasma HR, Nu Instruments) equipped with a desolvation nebulization membrane unit (Aridus II, Cetac) in combination with a PFA nebulizer (Microflow ST Nebulizer, Elemental Scientific, Omaha, USA) as sample introduction system were used. The ICP-QMS instrument was operated using the quantitative method option by Syngistix software. Measurements of isotopes ^202^Hg (20 ms), ^204^Pb (75 ms), ^206^Pb (20 ms), ^207^Pb (20 ms), ^208^Pb (20 ms) and ^115^In (20 ms) were performed with dwell times given in parenthesis and a total measurement time of ca. 3 min (1 sweep, 1 reading, 900 replicates), allowing the simultaneous Pb quantification and Pb isotope ratio analysis. The number of replicates was chosen to optimize the precision for determining the ^20x^Pb/^204^Pb isotope ratio.

The MC ICP-MS was operated in low mass resolution and static mode allowing for the simultaneous measurement of all natural stable Pb isotopes, as well as of ^202^Hg for monitoring the mercury background in the samples to correct a possible isobaric interference from ^204^Hg to ^204^Pb (see collector block configuration in Table S01). Data collection was accomplished in 6 blocks of 10 measurements with an integration time of 10 s, resulting in a total of 60 measurements per sample. The samples and the isotopic standards NIST SRM 981 were introduced into the plasma in the following sequence: standard_SSB1-SRM981_—sample – standard_SSB2-SRM981_, to enable correction for time-dependent instrumental isotopic fractionation (IIF) via classical sample standard bracketing (SSB). Concentrations of sample and SSB standard were matched within 10% [39, 40].

The final results of isotope ratio measurements are expressed as molar fractions (corresponding to an enrichment of ^204^Pb in the lead isotopic composition). A normalization to BMI (body mass index) and hematocrit was required for data obtained from blood samples to account for inter-subject variability. Normalization of the molar fraction of ^204^Pb tracer in urine was normalized to creatinine was also performed. In summary, ^204^Pb tracer enrichment factors assessed by mass spectrometric analysis and IPD were normalized by division with a correction factor calculated from BMI/hematocrit and from creatinine levels for data from blood and urine samples, respectively.

### Data processing

The Pb isotope data are given both in absolute notation as ^208^Pb/^204^Pb and in relative isotope notation with NIST SRM 981 acting as zero-anchor on the relative (= delta) scale. The actual enrichment in the samples due to the addition of the ^204^Pb tracer was calculated using isotope pattern deconvolution (IPD). This chemometric method is based on multiple linear regressions with assignment of the proportions of total lead to known end members of the mixture for the final isotopic composition in the sample (see equations below) [8].

#### Data processing: MC ICP-MS

1. Blank correction was performed using the *measure zero*—method provided by the instrument software automatically on-peak by subtracting the raw intensity of the analytical blank (calibration blank, *w* = 2% HNO_3_) from the raw intensity of each individual sample.
2. Outliers were removed on-the-run based on a 2*SD*-test (95% confidence interval) in the software.
3. Corrections for isobaric interferences: A correction approach referred to as “peak stripping” was applied to check for the impact of remaining Hg in the samples on the final Pb isotope ratio. This strategy was also applied to correct ^204^Pb^+^ for minor interferences of ^204^Hg^+^ arising from residual Hg (< 0.02%, expressed as relative ratio of *int*(^202^Hg)/*int*(^208^Pb)) in the purified sample solutions. Here, the simultaneously measured ^202^Hg (*int*(^202^Hg)_spl_) signal and the IUPAC/CIAAW value for *n*(^204^Hg)/*n*(^202^Hg)_nat_ (= 0.2293) [41], which was corrected for IIF via SSB with NIST SRM 981 of calculated isotopic compositions (*n*(^206^Pb)/n(^204^Pb)_cert_ = 16.937) [42], assuming the same IIF for *n*(^206^Pb)/*n*(^204^Pb)_spl_ and *n*(^204^Hg)/*n*(^202^Hg)_spl_, were used to calculate the ^204^Pb (*int*(^204^Pb)_spl_), according to Eqs. 1a–c:
  1a $${int\left({}^{204}Pb\right)}_{spl}={int(204)}_{spl}-{int({}^{202}Hg)}_{spl}\bullet {\left(\frac{n\left({}^{204}Hg\right)}{n\left({}^{202}Hg\right)}\right)}_{nat}\bullet {\left(\frac{M\left({}^{204}Hg\right)}{M\left({}^{202}Hg\right)}\right)}^{f}$$
  1b $$f=\frac{{f}_{{SSB}_{1}}+{f}_{{SSB}_{2}}}{2}$$
  1c $${f}_{SSB}=\mathrm{ln}\left(\frac{{\left(\frac{n({}^{206}Pb)}{n({}^{204}Pb)}\right)}_{cert}}{{\left(\frac{n({}^{206}Pb)}{n({}^{204}Pb)}\right)}_{SSB}}\right)/\mathrm{ln}\left(\frac{M\left({}^{206}Pb\right)}{M\left({}^{204}Pb\right)}\right)$$
  where all intensities correspond to blank corrected beam intensities, *int*(^206^Pb)/*int*(^204^Pb)_SSB_ was the measured raw ratio in the SSB standard, and *M*(X) were the atomic weights extracted from the IUPAC/CIAAW tables [41]. This correction was considered insignificant for real blood and urine samples and only applied during method optimization.
4. Blank corrected, averaged isotope ratios of ^204^Pb/^208^Pb, ^206^Pb/^208^Pb and ^207^Pb/^208^Pb were corrected for IIF by classical SSB using the isotope reference materials NIST SRM 981 (natural Pb). The NIST SRM 981 solutions (*w*(Pb) = 1 ng g^−1^) measured before and after a sample were used for IIF correction. Correction factors were calculated for each isotope ratio, respectively (*f*(^204^Pb/^208^Pb), *f*(^206^Pb/^208^Pb) and *f*(^207^Pb/^208^Pb)) and applied to correct the blank corrected, averaged isotope ratios of ^204^Pb/^208^Pb, ^206^Pb/^208^Pb and ^207^Pb/^208^Pb.
5. Blank and IIF corrected Pb isotope ratios followed isotope pattern deconvolution (IPD) to deconvolute the enrichment of ^204^Pb administration following equations Eqs. 2a and 2b. The isotopic composition of the ^204^Pb tracer (as provided in the certificate of the manufacturer) was taken as Pb source (= end-member) 1. The Pb isotopic abundances as determined in blood sample “BL” (corresponding to *t* = 0 before administration of the Pb-tracer) were taken as Pb source 2 providing the reference isotopic composition before artificial adulteration of the Pb isotopic composition in each individual. (Note: the natural Pb isotopic composition in human blood varies significantly between individuals. Thus, tabulated representative values for the natural Pb isotopic abundances cannot be used as sources/end-members in the IPD.) As there are more parameters (isotopic abundances) than unknowns (molar amounts of two sources) in the equation system, an error vector is included. (Note: a total of 4 sources of different Pb isotopic composition could be treated in the equation; in this study, only two sources were present: source 1: natural Pb; source 1: enriched ^204^Pb tracer.)
  2a $${A}_{s}^{i}=\frac{{R}_{i}}{{\sum }_{i=1}^{n}{R}_{i}}$$
  2b $$\left[\begin{array}{c}{A}_{s}^{204}\\ {A}_{s}^{206}\\ \begin{array}{c}{A}_{s}^{207}\\ {A}_{s}^{208}\end{array}\end{array}\right]=\left[\begin{array}{c}{A}_{source 1}^{204}\\ {A}_{source 1}^{206}\\ \begin{array}{c}{A}_{source 1}^{207}\\ {A}_{source 1}^{208}\end{array}\end{array}\right]\left[\begin{array}{c}{A}_{source 2}^{204}\\ {A}_{source 2}^{206}\\ \begin{array}{c}{A}_{source 2}^{207}\\ {A}_{source 2}^{208}\end{array}\end{array}\right]\left[\begin{array}{c}{A}_{source 3}^{204}\\ {A}_{source 3}^{206}\\ \begin{array}{c}{A}_{source 3}^{207}\\ {A}_{source 3}^{208}\end{array}\end{array}\right]\left[\begin{array}{c}{A}_{source 4}^{204}\\ {A}_{source 4}^{206}\\ \begin{array}{c}{A}_{source 4}^{207}\\ {A}_{source 4}^{208}\end{array}\end{array}\right]\cdot \left[\begin{array}{c}{x}_{source 1}\\ {x}_{source 2}\\ \begin{array}{c}{x}_{source 3}\\ {x}_{source 4}\end{array}\end{array}\right]+\left[\begin{array}{c}{e}^{204}\\ {e}^{206}\\ \begin{array}{c}{e}^{207}\\ {e}^{208}\end{array}\end{array}\right]$$
6. The molar fraction (in %), indicating the amount of ^204^Pb tracer (*x*(^204^Pb)) contributing to the total amount of Pb in blood as result of the IPD, was further normalized using HCT and BMI as provided by the study site. *x*(^204^Pb) was divided by the factor (HCT/BMI) for normalization, resulting in x-norm(^204^Pb). The average HCT (from two different measurements performed at the study site) was used. In case of urine, data were normalized to creatinine levels.

#### Data processing: ICP-QMS

Data was processed offline in Microsoft Excel® spreadsheet according to the following procedure point-by-point prior to averaging data.

1. Blank correction at each *m*/*z* was performed automatically by the Syngistix software by subtracting the raw intensity of the analytical blank (calibration blank, *w* = 2% HNO_3_) from the raw intensity of each individual sample.
2. As outlined above, no correction of the signal at *m*/*z* = 204 for ^204^Hg was necessary as Hg background could be considered negligible.
3. Blank corrected ratios of ^204^Pb/^208^Pb, ^206^Pb/^208^Pb and ^207^Pb/^208^Pb were calculated point-by-point, and the average and standard deviation of 9 blocks of 100 measurements were calculated. (Measurement statistics have previously been optimized to reach best precision for ^204^Pb/^208^Pb, ^206^Pb/^208^Pb and ^207^Pb/^208^Pb.)
4. IIF correction followed the similar procedure as described in detail for MC ICP-MS measurements.
5. IPD calculations following the exact same procedure as for MC ICP-MS measurements were performed.
6. x-norm(^204^Pb) was calculated as described above for MC ICP-MS measurements.
7. In addition, *δ*-values (in $\permille$ for Pb isotope ratios were calculated relative to the average isotopic ratio of the used reference standard (NIST SRM 981) from the SSB in accordance with Eq. 3:
  3 $$\begin{array}{cc}\delta{\left({}^iPb/{}^{204}Pb\right)}_{SRM981}=\left(\frac{R_{spl}-R_{SRM981}}{R_{SRM981}}\right)&\lbrack\permille\rbrack\end{array}$$

*δ*: delta value

^*i*^*Pb*: isotopes 206, 207 or 208 of Pb

*R*: isotope ratio of the sample (*spl*) or the standard (*SRM981*) after blank and interference correction of a specific ^i^Pb/^204^Pb ratio

#### Uncertainty calculations

The total combined uncertainty budget for Pb isotopic analysis was calculated using a simplified Kragten approach according to established protocols in our laboratory [39, 40, 43]. The precision of the measured isotope ratio of the sample and of the standards, as well as the within-run-repeatability of the measured isotope ratio in the bracketing standards as proxy for instrument stability, was taken into account as main contributors to the uncertainty.

The uncertainty of the x-norm(^204^Pb) was calculated by combining the total measurement uncertainty of the determination of ^204^Pb molar fraction and the total analytical error of HCT (*u* = 10%) as provided by the responsible laboratory [44].

## Results and discussion

### Blank levels of consumables and tracer vector

The total amount of Pb leached from consumables routinely used for blood sampling was compared to the detection and determination limits of the method. This showed that most materials did not contain significant amounts of leachable Pb. *LOD* (*γ*(Pb) = 0.013 µg L^−1^) and *LOQ* (*γ*(Pb) = 0.044 µg L^−1^) were determined using the analytical blank values (pure HNO_3_ (*w* = 2%) used for leaching experiments) via three or ten times the standard deviation of 10 repeated measurements of the analytical blank value from individually prepared solutions over the measurement sequence. Only measured values that were above the *LOQ* of the measurement were further processed. The examined materials showed no significant amounts of natural Pb especially considering the expected natural Pb levels in human blood as well as ^204^Pb tracer to be administered.

Only one material (Vacuette purple) showed leachable Pb levels above *LOQ* with *γ*(Pb) = 0.284 µg L^−1^. One product (Vacuette blue) showed increased levels of alkali and alkaline earth elements, which possibly originated from the internal heparin solutions contained. Based on the results of the leaching experiment, tubes “Vacuette green” were chosen for blood sampling during the clinical trial in order to avoid any cross contamination. Crown corks showed leached Pb amounts < *LOD*. The tracer vector mineral water (Vöslauer Still®) had Pb levels < *LOD*, as well.

To monitor the digestion blanks, 10 digestion blanks were processed. The lead background, measured on the raw signal of ^208^Pb in the digestion solutions diluted analogously to the measurement solutions, was 0.016 ng g^−1^ ± 0.005 ng g^−1^ Pb, thus considered insignificant.

### Blood CRM spiking experiments with ^204^Pb tracer

The calculation of the absolute Pb isotope ratios (in the following the ^208^Pb/^204^Pb isotope ratio is used) and the enrichment (based on IPD) was carried out via external calibration using an isotope-certified standard reference material. In addition, the influence of traces of mercury was estimated by measuring the signal of ^202^Hg.

Results of the spiking experiment shown in Table 1 show a significant shift in the ^208^Pb/^204^Pb isotopic composition already at a ^204^Pb enrichment level of 0.01% based on IPD. No significant effect was detected based on possible contribution of traces of Hg in the sample, and thus, Hg correction was considered insignificant for further analysis.

**Table 1:** Results of the spiking experiments using BCR-636 and ^204^Pb tracer. Isotope ratios reported as absolute and relative values (relative to NIST SRM 981), with and without correction for isobaric interference from ^204^Hg to ^204^Pb

Sample ID	^208^Pb/^204^Pb (no Hg corr)	^208^Pb/^204^Pb (Hg corr)	*δ* Pb-^208^/^204^ / ‰ (no Hg corr)	*δ* Pb-^208^/^204^ / ‰ (Hg corr)
BCR-363 (no ^204^Pb spike added)	38.259	38.281	41.85	42.46
BCR-363 (no ^204^Pb spike added)	38.235	38.246	41.21	41.50
BCR-363 0.01% enriched in ^204^Pb	37.955	37.987	33.58	34.45
BCR-363 0.02% enriched in ^204^Pb	37.758	37.759	28.21	28.25
BCR-363 0.05% enriched in ^204^Pb	36.954	36.995	6.31	7.45
BCR-363 0.10% enriched in ^204^Pb	35.687	35.650	− 28.18	− 29.20
BCR-363 0.15% enriched in ^204^Pb	34.570	34.586	− 58.60	− 58.18
BCR-363 0.15% enriched in ^204^Pb	34.471	34.506	− 61.28	− 60.33
BCR-363 0.30% enriched in ^204^Pb	31.327	31.307	− 146.91	− 147.46
BCR-363 0.50% enriched in ^204^Pb	27.799	27.812	− 242.99	− 242.65

**Fig. 1:**
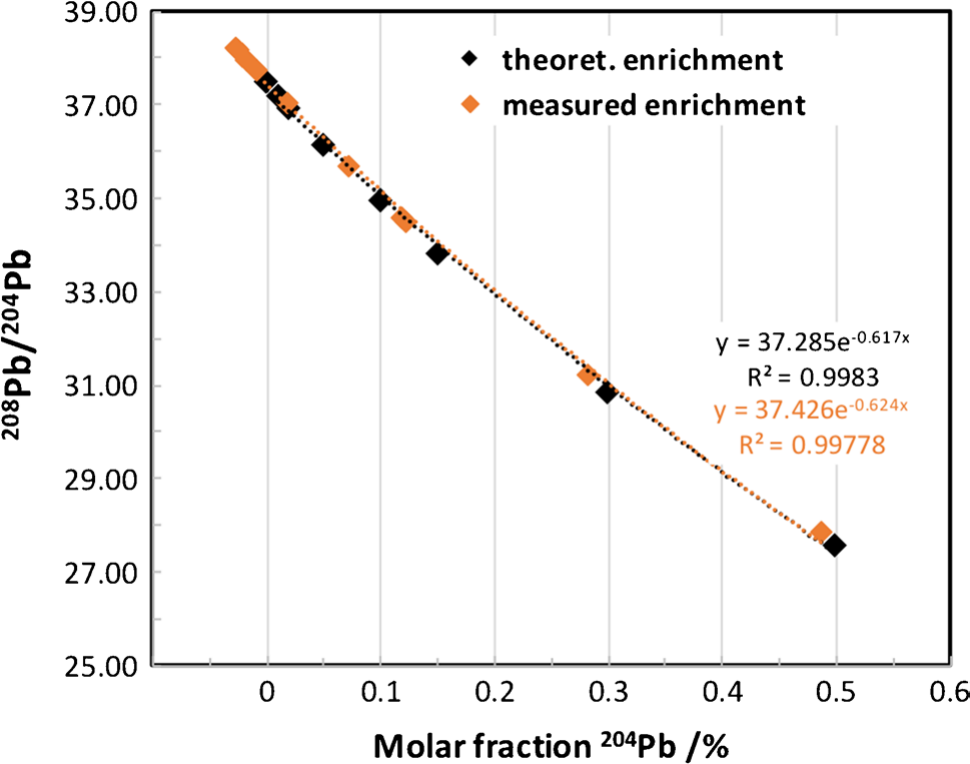
Molar fraction of ^204^Pb tracer calculated by IPD vs. measured ^208^Pb/^204^Pb ratio. The theoretical enrichment levels from experimental design are compared to the actual measured enrichment levels assessed from spiking experiments using BCR-636 spiked with increasing amounts of ^204^Pb tracer solution

Figure 1 shows the molar fraction of ^204^Pb calculated based on IPD vs the absolute ^208^Pb/^204^Pb ratio.

The theoretically calculated change in isotopic composition corresponds very well with the signature achieved in the spike experiments. The natural Pb isotopic composition in the blood based on tabulated values deviates from the estimated calculated values, albeit, not significantly. As a consequence, the true natural Pb isotopic composition was determined for each blood sample from the clinical trial and used as end member for IPD calculations. In the expected degree of enrichment of 0.15% with an administration of ^204^Pb at the limit of the drinking water ordinance of 10 μg L^−1^ in 250 mL Vöslauer mineral water, a shift in the ^208^Pb/^204^Pb ratio from 38.25 to 34.50 was to be expected. This corresponds to a relative change of approx. 9–10%. The average total measurement uncertainty (both for MC ICP-MS and ICP-QMS measurements) of the ^208^Pb/^204^Pb was approx. 0.06% with a measurement signal of ^204^Pb > 0.004 mV. Accordingly, the bias to be expected in the human experiments was significant with regard to the measurement uncertainty. In conclusion, the method including the data processing based on IPD was considered valid for the carrying out the clinical trial using the protocol outlined above.

### Comparison of MC ICP-MS and ICP-QMS for Pb isotope ratio assessment in real blood and urine samples

Pb isotope ratio data and enrichment based on ^204^Pb administration was obtained in blood of 2 subjects (subject-01 and subject-02) by both MC ICP-MS and ICP-QMS for comparison of instrument performance at low levels of Pb.

Data obtained by MC ICP-MS and ICP-QMS were compared for subject-01 and subject-02 (see Tables 2 and 3).

**Table 2:** Comparison of results obtained by MC ICP-MS and ICP-QMS. Molar fraction of ^204^Pb tracer (normalized to HCT/BMI) by IPD of subject-01 for *t* = 0, 4, 8, 12, 24, 48 and 192 h

Sample ID	Molar fraction ^204^Pb tracer (%)		Molar fraction ^204^Pb tracer (%) (normalized to HCT/BMI)	
	ICP-QMS	MC ICP-MS	ICP-QMS	MC ICP-MS
	*RSD* (*k* = 1) = 3%	*RSD* (*k* = 1) = 3%	*u*_rel_ (*k* = 1) = 10%	*u*_rel_ (*k* = 1) = 10%
G-LEAD_01_BL_B1_DIG	0.0000	0.0000	0.0000	0.0000
G-LEAD_01_4h_B1_DIG	0.0541	0.0425	0.0245	0.0193
G-LEAD_01_8h_B1_DIG	0.0764	0.0704	0.0347	0.0320
G-LEAD_01_12h_B1_DIG	0.0840	0.0861	0.0381	0.0391
G-LEAD_01_24h_B1_DIG	0.0981	0.0970	0.0445	0.0440
G-LEAD_01_48h_B1_DIG	0.1126	0.1221	0.0511	0.0554
G-LEAD_01_192h_B1_DIG	0.0997	0.1224	0.0453	0.0555

**Table 3:** Comparison of results obtained by MC ICP-MS and ICP-QMS. Molar fraction of ^204^Pb tracer (normalized to HCT/BMI) by IPD of subject-02 for *t* = 0, 4, 8, 12, 24, 48 and 192 h

Sample ID	Molar fraction ^204^Pb tracer (%)		Molar fraction ^204^Pb tracer (%) (normalized to HCT/BMI)	
	ICP-QMS	MC ICP-MS	ICP-QMS	MC ICP-MS
	*RSD* (*k* = 1) = 3%	*RSD* (*k* = 1) = 3%	*u*_*rel*_ (*k* = 1) = 10%	*u *_*rel*_ (*k* = 1) = 10%
G-LEAD_02_BL_B1_DIG	0.0000	0.0000	0.0000	0.0000
G-LEAD_02_4h_B1_DIG	0.5531	0.5363	0.2572	0.2493
G-LEAD_02_8h_B1_DIG	0.9240	0.9016	0.4296	0.4192
G-LEAD_02_12h_B1_DIG	1.0974	1.0781	0.5103	0.5013
G-LEAD_02_24h_B1_DIG	1.1618	1.2073	0.5402	0.5613
G-LEAD_02_48h_B1_DIG	1.2525	1.2637	0.5824	0.5876
G-LEAD_02_192h_B1_DIG	1.1979	1.2419	0.5570	0.5774

**Fig. 2:**
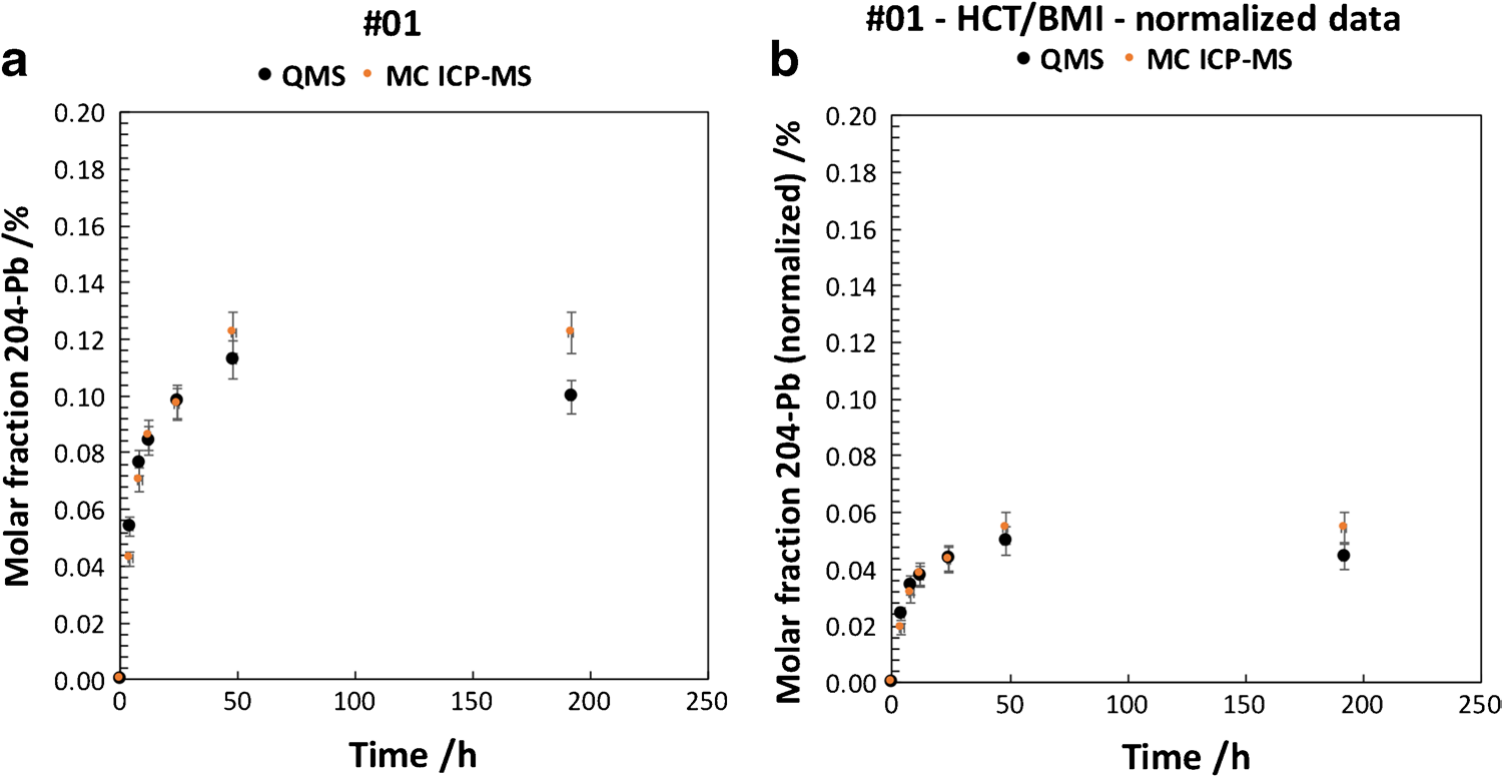
**a** (left) Molar fraction of ^204^Pb tracer by IPD of subject-01 vs sampling time after administration of tracer; error bars represent 1*SD* of the measurement. **b** (right) Molar fraction of ^204^Pb tracer normalized for HCT/BMI by IPD of subject-01 vs sampling time; error bars represent combined uncertainties *u* (*k* = 1) of the normalized molar fraction

**Fig. 3:**
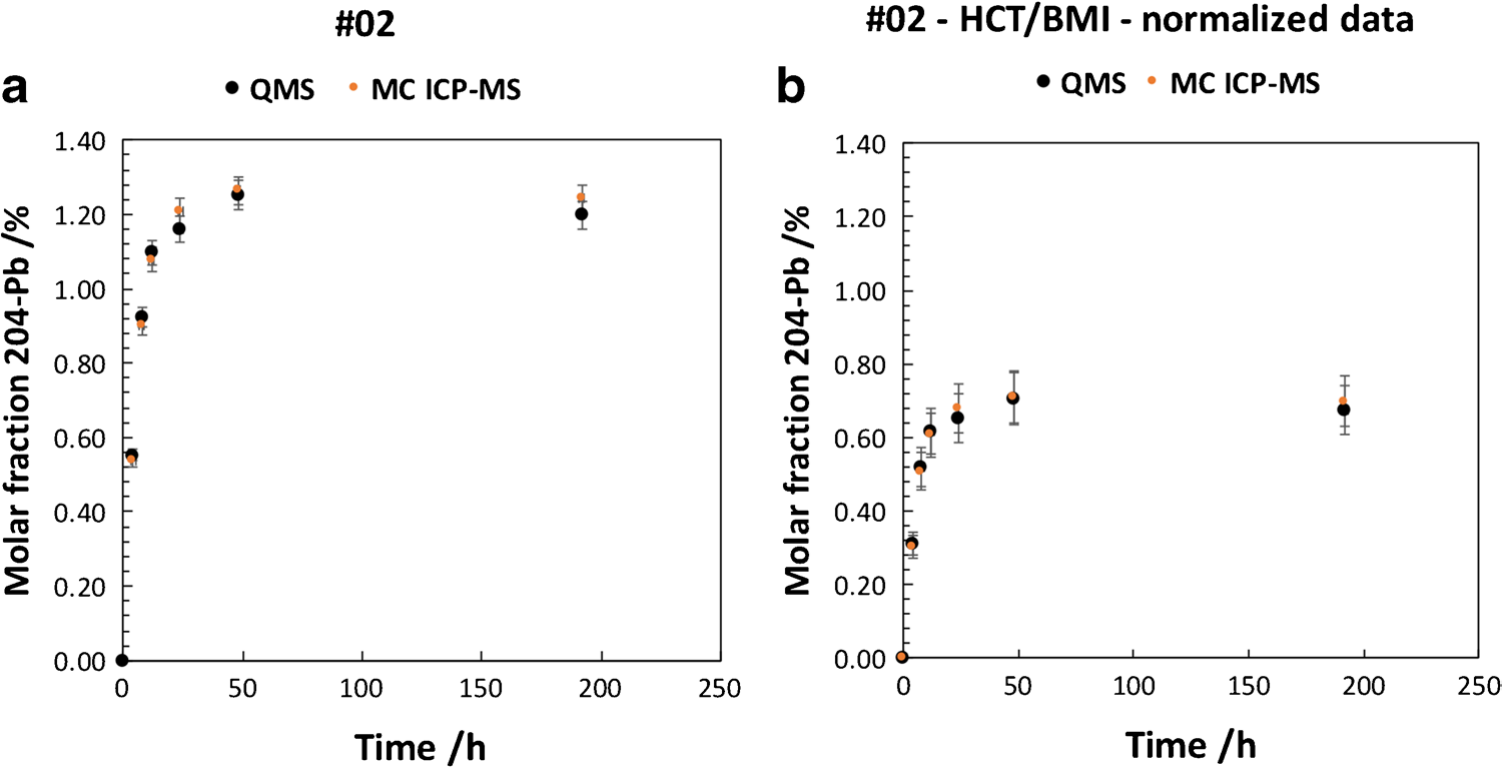
**a** (left) Molar fraction of ^204^Pb tracer by IPD of subject-02 vs sampling time after administration of tracer; error bars represent 1*SD* of the measurement. **b** (right) Molar fraction of ^204^Pb tracer normalized for HCT/BMI by IPD of subject-02 vs sampling time; error bars represent combined uncertainties *u* (*k* = 1) of the normalized molar fraction

Figure 2a  and b shows the molar fraction of ^204^Pb contributing to the total Pb in each sample of subject-01 obtained by IPD including a comparison of data obtained by MC ICP-MS and ICP-QMS. Figure 3a  and b shows the molar fraction of ^204^Pb contributing to the total Pb in each sample of subject-02 obtained by IPD including a comparison of data obtained by MC ICP-MS and ICP-QMS. Uncertainties plotted in Figs. 2b and 3b correspond to combined uncertainties taking into account the measurement of ^204^Pb (*RSD* = 3%) and the total analytical error of HCT determination (*u* = 10%).

Molar fractions (in *x*(^204^Pb) %; representing the amount of Pb originating from the ^204^Pb tracer) show similar kinetic curves for subject-01 and subject-02 even though the degree of enrichment differs significantly between the subjects. Data obtained by ICP-QMS and MC ICP-MS do not differ significantly taking into account the combined measurement uncertainty. The combined uncertainty budget considered the precision of the single measurement and the total analytical error of HCT determination. A possible uncertainty of the BMI to the total uncertainty of the final result was considered insignificant and thus not propagated.

The accuracy (considering uncertainty and trueness) for ICP-QMS and MC ICP-MS is considered equal. This can be explained by the very low analyte concentrations in the measurement solutions as a result of the low Pb mass fraction in the original samples. MC ICP-MS would usually be considered the method of choice for isotope-ratio analysis based on the improved precisions that can be obtained for isotope ratio analysis given that ideal measurement conditions are met. However, the ICP-QMS setup tested in this study represented the more sensitive method for measuring low concentrations. In this particular case, total Pb levels were assessed in the low ng g^−1^ to pg g^−1^ range. (Note: the natural ^204^Pb abundance is about 1.4%.) In case of MC ICP-MS measurements, ^204^Pb was assessed at signal intensities of ca. 0.001 V, resulting in decreased measurement precision by 3–4 orders of magnitude as compared to ideal conditions. In addition, the routine sample introduction system (desolvation membrane Aridus II) of MC ICP-MS, which achieves high sensitivity, cannot be used with pre-concentrated blood and urine samples due to the present high salt concentrations. It is possible to subject samples to a purification step as usually performed with samples of high ng g^−1^ to μg g^−1^ Pb levels. This was however not considered because conventional purification procedures use significant amounts of purification solutions that potentially carry the analyte in ultra-traces as well. In this case, the purification would significantly increase the Pb background levels in the purified sample solution. Subsequently, the measurements to determine enrichment of ^204^Pb administration in blood and urine were performed by ICP-QMS as robust and reliable method for all subjects.

### Measurement method by ICP-QMS: blood matrix

The Pb mass fraction *w*(Pb) of the measurement solutions of blood digests (dilution of the digestion solution by a factor of 4) ranged from 0.052 to 0.538 ng g^−1^ (this corresponds to *w*(^204^Pb) = 0.73 to 7.53 pg g^−1^). The detected (blank corrected) signal intensities of ^208^Pb and ^204^Pb were < 300 counts and < 50 counts, respectively. The average *LOD* of the analytical measurement was 4.0 pg g^−1^ (for total Pb) corresponding to 0.02 pg g^−1 204^Pb.

Method blanks showed raw signal intensities of ^208^Pb and ^204^Pb of < 80 counts and < 10 counts, respectively, resulting in Pb mass fractions ranging from *w*(Pb) = 0.010 ng g^−1^ to 0.020 ng g^−1^ (*w*(^204^Pb) = 0.00014 ng g^−1^ to 0.00028 ng g^−1^) in the method blank solutions. The resulting *LOD* of the method for total Pb determination in blood was *w*(Pb) = 0.09 ng g^−1^. The resulting *LOD* of the method for ^204^Pb determination in blood was *w*(^204^Pb) = 0.001 ng g^−1^.

BCR-634 (*γ*(Pb) = 46.0 µg L^−1^ ± 5.0 µg L^−1^ (*U*)) and BCR-635 (*γ*(Pb) = 210.0 µg L^−1^ ± 24.0 µg L^−1^ (*U*)) (Pb in blood) were processed in replicates along with blood samples. The two materials were chosen based on the certified Pb levels. The average *γ*(Pb) in BCR-634 and BCR-635 determined was 48.0 µg L^−1^ ± 19.0 µg L^−1^ (*n* = 21; 2*SD*) and 208.2 µg L^−1^ ± 57.3 µg L^−1^ (*n* = 22; 2*SD*). Pb concentrations determined in BCR-634 and BCR-635 processed during blood sample preparation overlap within uncertainties with the certified values.

### Measurement method by ICP-QMS: urine matrix

The mass fraction of natural Pb in the measurement solutions of urine digests (dilution by a factor of 4 from the original digestion solution) in ICP-QMS ranged from 0.010 to 0.030 µg L^−1^ (this corresponds to *γ*(^204^Pb) = 0.00014 to 0.00042 µg L^−1^). The detected (blank corrected) signal intensities of ^208^Pb and ^204^Pb were < 70 counts and < 10 counts, respectively. Method blanks showed raw signal intensities for ^208^Pb and ^204^Pb of < 17 counts and < 0.2 counts, respectively, resulting in an average Pb mass fraction *c*(Pb) = 0.009 µg L^−1^ (*γ*(^204^Pb) = 0.00013 µg L^−1^) in the method blank solutions. The *LOD* of the method for ^204^Pb tracer determination in urine is *γ*(^204^Pb) = 0.001 µg L^−1^.

Seronorm Trace Elements Urine L-1 (certified *γ*(Pb) = 0.72 µg L^−1^ ± 0.36 µg L^−1^ (as indicative *U*) was processed in replicates along with urine samples. The average *β*(Pb) in Seronorm Trace Elements Urine L-1 determined was 1.75 µg L^−1^ ± 1.25 µg L^−1^ (*n* = 18; 2*SD*). Pb concentrations determined in Seronorm L-1 processed during urine sample preparation overlap within uncertainties with the certified values. The relative standard deviation of a measurement was about 30% as a result of the substantial low signal intensities (*I*(^208^Pb) ranging from 40 to 60 counts). Pb concentrations were corrected for the procedural blank (= method blank from microwave digestion).

### Results from blood samples of all subjects from the clinical trial

The total amount of Pb and the enrichment of ^204^Pb by the administered tracer were normalized to HCT and BMI—values for each individual. Table S03 summarizes all data obtained in blood samples of 42 subjects (investigated in a two-stage approach: stage 1 (6 subjects) and stage 2 (36 subjects)) (note: sampling time was adjusted based on actual sampling times for each individual and rounded to an hour). The natural Pb content in the blood of the examined subjects ranged from 4.3 to 52.1 µg L^−1^ with an average of 16.1 µg L^−1^ ± 9.7 µg L^−1^ (1 *SD*).

Combined uncertainties were calculated by propagating the uncertainty of a single measurement as well as the total analytical error of HCT determination. The major contributor to the overall uncertainty of the primary end point is represented by the HCT value used for normalization with a contribution of > 90%. The uncertainty of the measurement contributes to the total uncertainty by < 10%. Subjects show a significantly different natural Pb isotopic composition in sample BL (*t* = 0). The ^204^Pb/^208^Pb isotope ratio at *t* = 0 before administration of the ^204^Pb tracer ranged from 0.026 to 0.038 with an average of 0.028 ± 0.002 (1 *SD*). This significant variation underlines the importance of using the measured natural Pb isotopic abundance as end member (= source 1) used in isotope pattern deconvolution instead of using tabulated representative natural Pb isotopic composition [8].

Figure S01–S42 show the relative enrichment (molar fraction of ^204^Pb tracer contributing to the total amount of Pb in blood normalized to HCT and BMI) for the 42 subjects (study phase 1 (6 subjects) and study phase 2 (36 subjects)) as a function of time. Error bars correspond to combined uncertainties *u* = 10% (*k* = 1). Subject-02 shows the highest enrichment of ^204^Pb, thus determining the maximum for the graphical visualization on the y-axis.

**Fig. 4:**
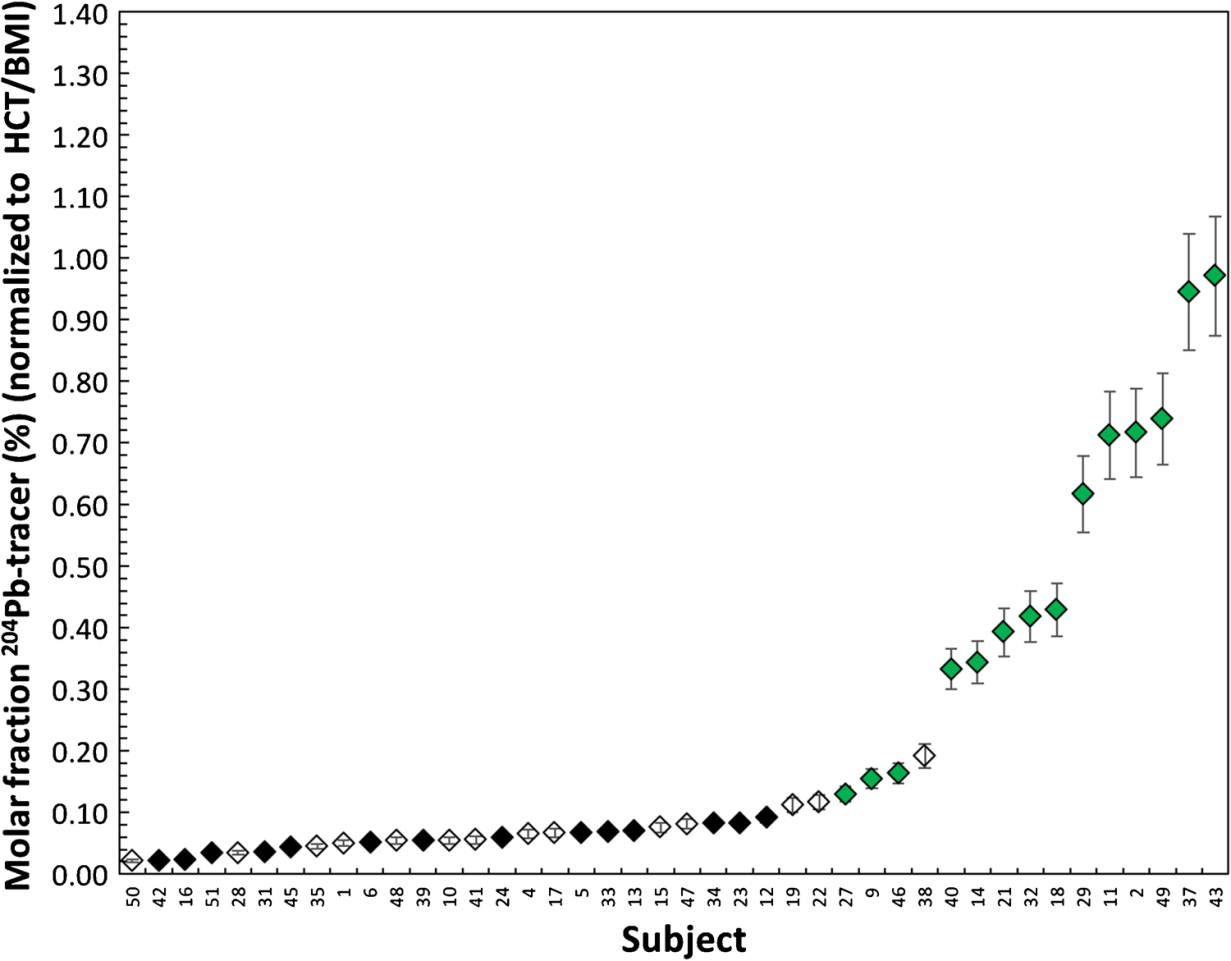
The maximum enrichment as ^204^Pb-molar fraction in Pb normalized to HCT/BMI of all 42 subjects from the three treatment groups (placebo, 1 × 2.0 g G-PUR and 2 × 2.0 g G-PUR) (independent of the time of maximum enrichment in tracer) sorted by enrichment, error bars correspond to *u* = 10% (*k* = 1)

All subjects show enrichment of the ^204^Pb spike from BL (*t* = 0) to follow-up sampling times. The maximum accumulation in the blood occurs earliest at 24 h after administration. Figure 4 shows the maximum enrichment as ^204^Pb-molar fraction in Pb normalized to HCT/BMI of all 42 subjects from the three treatment groups after un-blinding of the treatment of each individual. The mean maximum ^204^Pb tracer enrichment was significantly higher in the placebo group compared to the treated groups including the IMD.

The enrichment at ^204^Pb in blood differed significantly between the subjects. This allowed for assessing the efficacy of the administered clinoptilolite. The further medical interpretation of the results behind the clinical trial are discussed in Samekova et al. [16].

### Results from urine samples of all subjects from clinical trial

The total concentration of Pb in urine samples was determined by ICP-QMS and further normalized to creatinine in urine as provided (see Table S04). All subjects show enrichment of the ^204^Pb spike from BL (*t* = 0) to 24 h (*t* = 24 h)-urine sample.

Data from urine of subject-04 could not successfully be evaluated as natural Pb from the background of the storage vessel (as a not pre-cleaned vessel was used accidently) significantly influenced the Pb background. A bias in the input sources to the regression model results in failure of the mathematical procedure. The enrichment of ^204^Pb differs significantly between the evaluated subjects. The relative uncertainties of > 60% can be explained by the low levels of Pb present in the samples. The major contributor to the uncertainty is the precision of the ICP-QMS measurement with a contribution of > 98% to the overall uncertainty, while the analytical error of creatinine determination amounted to < 2%. Excretion of ^204^Pb in 24-h urine was also decreased in subjects receiving the IMD compared to the placebo group. The medical implications of the results, which were obtained in the clinical trial, are discussed in Samekova et al. [16].

## Conclusion

In this study, we successfully proved the applicability of isotope pattern deconvolution for data reduction when low levels of enriched isotope tracer (here: ^204^Pb) are orally administered (levels below the Austrian drinking water regulation (i.e. *c*(Pb) < 10 µg L^−1^)). The results showed for the first time that IPD allowed for the determination of tracer at levels of < 1 pg ^204^Pb/g matrix in a biological system, provided that comprehensive blank investigations are performed and that full control of the blank is achieved. ICP-QMS was selected over MC ICP-MS for Pb isotope ratio analysis, because sensitivity was the limiting factor. In this approach, we were able to measure isotope ratios of Pb with signals > 50 counts for the tracer isotope ^204^Pb. We stress that there was a substantial contribution by the uncertainty of clinical parameters used for normalization of Pb in blood and urine (BMI/HCT and CREA, respectively). Thus, the overall uncertainty was not determined by the measurement but by the normalization parameters. The combined uncertainty of enriched ^204^Pb molar fraction in blood of *U* (*k* = 2) was 20% and thus met the methodological requirement to determine a significant enrichment of the tracer. Finally, it is also conceivable that, due to sequential measurement of the isotopes of interest, the use of MC ICP-MS instead of single collector ICP-QMS may result in a further improvement of uncertainty.

The current study provides a proof-of-principle for employing enriched stable isotope tracers in clinical studies: the ultra-low levels required for the investigation keeps the administered amounts of tracer below any levels of concern.

## Supplementary Information

Below is the link to the electronic supplementary material.

Supplementary file1 (PDF 773 KB)

## Footnotes

1 Note that chemical symbols were used according to the IUPAC Goldbook [35]
