# Supplementary material for: Enriched stable 204Pb as tracer at ultra-low levels in clinical investigations
Source: Anal Bioanal Chem. 2022 Sep 22;415(2):255–68. doi: 10.1007/s00216-022-04311-0 (PMC9823027; doi:10.1007/s00216-022-04311-0)
Supplement: Supplementary file 1 — Supplementary file1 (PDF 773 KB) [file 216_2022_4311_MOESM1_ESM.pdf]

Supporting information

Enriched stable ^{204}Pb as tracer at ultra-low levels in clinical investigations

Johanna Irrgeher[1,*], Thomas Berger[2§], Anastassiya Tchaikovsky[2], Cornelius Tschegg[2], Ghazaleh Gouya[3], Peter Lechner[4], Anika Retzmann[1§], Christine Opper[1§,5], Christa Firbas[6], Michael Freissmuth[7], Kerstin Peschl-Credner[3], Karolina Anderle[7], Claudia Meisslitzer[2], Michael Wolzt[6], Thomas Prohaska[1]

[1] Chair of General and Analytical Chemistry, Department of General, Analytical and Physical Chemistry, Montanuniversität Leoben, Franz-Josef-Straße 18, 8700 Leoben, Austria

[2] Glock Health Science and Research GmbH, Hausfeldstraße 17, 2232 Deutsch-Wagram, Austria

[3] Gouya Insights, Elisabethstrasse 22/12, 1010 Vienna, Austria

[4] LGS-INSIGHTS GmbH, Elisabethstrasse 22/12, 1010 Vienna, Austria

[5] TB Unterfrauner GmbH, Umseerstraße 39, 3040 Neulengbach, Austria.

[6] Department of Clinical Pharmacology, Medical University of Vienna, Währinger Gürtel 18-20, 1090 Vienna, Austria

[7] Institute of Pharmacology and the Gaston H. Glock Research Laboratories for Exploratory Drug Development, Center of Physiology and Pharmacology, Medical University of Vienna, Währingerstrasse 13a, Vienna, Austria

[§] affiliation during the time the work was accomplished

* Corresponding author: Johanna Irrgeher

Email: johanna.irrgeher@unileoben.ac.at

Table S01

Table S01 Detector configurations for Pb isotopic analysis using Nu Plasma HR (NP048)

Nu Plasma HR

**F**	**F**	**F**	**F**	**F**	**F**	**F**	**F**	**F**	**IC**	**F**	**IC**	**F**	**IC**	**F**
**H6**	**H5**	**H4**	**H3**	**H2**	**H1**	**Ax**	**L1**	**L2**	**IC0**	**L3**	**IC1**	**L4**	**IC2**	**L5**
		^{208}**Pb**	^{207}**Pb**	^{206}**Pb**	^{205}**Tl**	^{204}**Pb**	^{203}**Tl**	^{202}**Hg**						

SI Elemental analysis by ICP-MS

Quantification of Pb and selected elements (Li, Be, B, Na, Mg, Al, K, Ca, V, Cr, Fe, Mn, Co, Ni, Cu, Zn, Ga, As, Se, Rb, Sr, Mo, Ag, Cd, Te, Ba, Nd, Tl, Bi and U) of the samples were performed using a quadrupole inductively coupled plasma mass spectrometry, ICP-QMS (NexION 2000, PerkinElmer, Ontario, Canada) coupled to an ESI SC-2 DX FAST autosampler. The instrument was optimized in a daily routine using a tuning solution, containing Li, Y, Ce, Tl or Be, In, Ce and U to maintain a reliable day-to-day-performance.

Samples were diluted by a factor of 50 for the analysis of Na, Mg, K and Fe using HNO_3 (w = 2 %). Samples were diluted by a factor of 4 for the analysis of Li, Be, B, Al, Ca, V, Cr, Mn, Co, Ni, Cu, Zn, Ga, As, Se, Rb, Sr, Mo, Ag, Cd, Te, Ba, Nd, Tl, Bi and U using ultrapure water.

ICP-QMS measurements were accomplished performing an 11-point linear external calibration (0.05 ng g^{-1}, 0.1 ng g^{-1}, 0.5 ng g^{-1}, 1 ng g^{-1}, 5 ng g^{-1}, 10 ng g^{-1}, 25 ng g^{-1}, 50 ng g^{-1}, 100 ng g^{-1}, 150 ng g^{-1}, 175 ng g^{-1}, prepared gravimetrically from a Merck multi-element standard VI (Merck, Darmstadt, Germany)) for all elements except Nd and a 5-point linear external calibration (0.25 ng g^{-1}, 2.5 ng g^{-1}, 25 ng g^{-1}, 125 ng g^{-1}, 250 ng g^{-1}), prepared gravimetrically from a AHF CAL 7 standard (Lot. Nr. A2-MEB238041, AHF, Tübingen, Deutschland), for the determination of Nd.

QC standards: In-house QC-standard solutions prepared from single-element ICP Standards (in HNO_3, w = 2 %) were measured repeatedly during the measurement sequence as quality control of the quantification. All standards, samples and blanks were measured including 1 ng g-1 indium (In) used as internal normalization standard (Merck-Millipore), which was introduced via on-line addition.

Selected isotopes: ^{7}Li, ^{9}Be, ^{11}B, ^{23}Na, ^{24}Mg, ^{27}Al, ^{39}K, ^{44}Ca, ^{51}V, ^{52}Cr, ^{55}Mn, ^{57}Fe, ^{59}Co, ^{60}Ni, ^{65}Cu, ^{68}Zn, ^{71}Ga, ^{75}As, ^{82}Se, ^{85}Rb, ^{88}Sr, ^{98}Mo, ^{107}Ag, ^{111}Cd, ^{128}Te, ^{138}Ba, ^{143}Nd, ^{205}Tl, ^{209}Bi, ^{238}U.

Instrumental parameters are given in **Table S02**.

Table S02 Instrumental parameter Nexion 2000b ICP-QMS

In intensity (1 ng g-1): 210 000 – 245 000 cps

Oxide rate: < 3 %

Rate of doubly charged ions: < 2 %

Ar neb. gas flow (L min-1): 0.97 – 1.00

Ar plasma gas flow (L min-1): 16.00

Ar auxiliary gas flow (L min-1): 1.20

Analog stage voltage (V): -2000

Pulse stage voltage (V): 1250

Data was processed using the Syngistix software for external calibration and further processed offline in Microsoft Excel spreadsheets considering the following aspects:

1. Blank correction at each *m*/*z* was performed automatically by the Syngistix software by subtracting the In-normalized raw intensity of the analytical blank.

2. Calibration curves were calculated automatically by the Syngistix software from ^{115}In-normalized-blank-corrected intensities and the gravimetrically prepared mass fraction of the calibration standards (Merck, AHF). Elemental mass fractions of each sample were calculated automatically by the Syngistix software, respectively.

3. QC-control: QC standards were measured periodically during the sequence. The validity interval was set to $\pm$ 15 % of the gravimetrically prepared standards. Analytes which are not of relevance for the further interpretation of the analytical data and were outside the +/- 15 % range were rejected and not considered further. This accounts for V and Te.

4. Procedural blank: Average elemental mass fraction of the method blank was calculated for each isotope.

5. The limits of detection and quantification (*LOD*, *LOQ*) were calculated as follows: The *LOD* was defined as elemental mass fraction of method blank (calculated automatically by the Syngistix software) plus 3 x *SD* of the method blank, the *LOQ* was defined as elemental mass fraction of method blank plus 10 x *SD* of the method blank. Only data above *LOQ* was considered further. *LOD* and *LOQ* values for urine and blood were calculated taking into account the dilution factor, average digest volume and sample volume.

6. Elemental mass fractions (calculated automatically by the Syngistix software) were corrected by the average method blank value. Total elemental concentrations of each sample were calculated considering the dilution factor, the individual volume after transfer of the digest, the sample volume and sample density.

7. Average elemental concentration and *SD* as well as minimum and maximum elemental concentration values for all samples were calculated. The average elemental concentration and *SD* in blood for each subject were calculated.

8. Uncertainties for the elemental concentration were calculated using a simplified Kragten approach considering the standard deviation of the raw intensities of the blank and the sample as well as the slope of the calibration curve.

Table S03 Results of Pb analysis by ICP-QMS in blood samples (t_{max} is highlighted in bold letters; ****primary endpoint***); n/a sample was not available.

1	2	3	4	5	6	7	8	9	10	11*	12	13	14
**SAMPLE ID**	**SUBJECT**	**time**	**Total Pb in sample (µg/L)**	**2 *SD***	**$^{204}Pb/^{208}Pb$**	**1 *SD***	**molar fraction ^{204}Pb-tracer (%)**	**BMI**	**HCT (%)**	**molar fraction ^{204}Pb-tracer (%) (normalized to HCT/BMI)***	***u* (10%)**	**absolute amount of ^{204}Pb from tracer (µg/L) - normalized to HCT/BMI**	***u* (10%)**
G-LEAD_01_BL_B1_DIG	1	0	14.0	4.2	0.0266	0.0004	0.000	20.6	46.2	0.000	0.000	0.0000	0.0000
G-LEAD_01_4h_B1_DIG	1	4	13.0	3.9	0.0280	0.0006	0.054	20.6	46.2	0.024	0.002	0.0031	0.0003
G-LEAD_01_8h_B1_DIG	1	8	12.8	3.8	0.0280	0.0005	0.076	20.6	46.2	0.034	0.003	0.0044	0.0004
G-LEAD_01_12h_B1_DIG	1	12	15.0	4.5	0.0282	0.0004	0.084	20.6	46.2	0.037	0.004	0.0056	0.0006
G-LEAD_01_24h_B1_DIG	1	24	15.2	4.6	0.0284	0.0004	0.098	20.6	46.2	0.044	0.004	0.0067	0.0007
G-LEAD_01_48h_B1_DIG	1	49	13.6	4.1	0.0288	0.0008	**0.113**	20.6	46.2	**0.050**	0.005	0.0068	0.0007
G-LEAD_01_192h_B1_DIG	1	193	14.4	4.3	0.0285	0.0006	0.100	20.6	46.2	0.044	0.004	0.0064	0.0006
G-LEAD_02_BL_B1_DIG	2	0	10.9	3.3	0.0269	0.0004	0.000	21.76	38.7	0.000	0.000	0.0000	0.0000
G-LEAD_02_4h_B1_DIG	2	4	11.9	3.6	0.0376	0.0006	0.553	21.76	38.7	0.302	0.030	0.0360	0.0036
G-LEAD_02_8h_B1_DIG	2	8	10.6	3.2	0.0448	0.0011	0.924	21.76	38.7	0.509	0.051	0.0541	0.0054
G-LEAD_02_12h_B1_DIG	2	12	9.7	2.9	0.0481	0.0014	1.097	21.76	38.7	0.610	0.061	0.0593	0.0059
G-LEAD_02_24h_B1_DIG	2	24	11.5	3.4	0.0494	0.0012	1.162	21.76	38.7	0.684	0.068	0.0786	0.0079
G-LEAD_02_48h_B1_DIG	2	49	12.5	3.7	0.0511	0.0014	**1.253**	21.76	38.7	**0.716**	0.072	0.0894	0.0089
G-LEAD_02_192h_B1_DIG	2	193	12.9	3.9	0.0501	0.0007	1.198	21.76	38.7	0.704	0.070	0.0909	0.0091
G-LEAD_04_BL_B1_DIG	4	0	13.2	4.0	0.0318	0.0007	0.000	24.17	38.2	0.000	0.000	0.0000	0.0000
G-LEAD_04_4h_B1_DIG	4	4	14.4	4.3	0.0324	0.0011	0.036	24.17	38.2	0.023	0.002	0.0033	0.0003
G-LEAD_04_8h_B1_DIG	4	8	13.6	4.1	0.0325	0.0006	0.040	24.17	38.2	0.026	0.003	0.0035	0.0003
G-LEAD_04_12h_B1_DIG	4	12	13.9	4.2	0.0332	0.0006	0.075	24.17	38.2	0.047	0.005	0.0066	0.0007
G-LEAD_04_24h_B1_DIG	4	24	15.4	4.6	0.0331	0.0007	0.076	24.17	38.2	0.048	0.005	0.0074	0.0007
G-LEAD_04_48h_B1_DIG	4	49	16.5	4.9	0.0337	0.0005	**0.103**	24.17	38.2	**0.065**	0.007	0.0107	0.0011
G-LEAD_04_192h_B1_DIG	4	193	12.6	3.8	0.0336	0.0006	0.100	24.17	38.2	0.063	0.006	0.0079	0.0008
G-LEAD_05_BL_B2_DIG	5	0	14.3	4.3	0.0294	0.0005	0.000	22.96	40.3	0.000	0.000	0.0000	0.0000
G-LEAD_05_4h_B2_DIG	5	4	15.1	4.5	0.0303	0.0006	0.045	22.96	40.3	0.026	0.003	0.0039	0.0004
G-LEAD_05_8h_B2_DIG	5	8	13.7	4.1	0.0306	0.0009	0.061	22.96	40.3	0.035	0.003	0.0048	0.0005
G-LEAD_05_12h_B2_DIG	5	12	13.3	4.0	0.0309	0.0009	0.076	22.96	40.3	0.044	0.004	0.0058	0.0006
G-LEAD_05_24h_B2_DIG	5	24	12.7	3.8	0.0310	0.0005	0.081	22.96	40.3	0.046	0.005	0.0059	0.0006
G-LEAD_05_48h_B2_DIG	5	49	12.1	3.6	0.0317	0.0009	**0.118**	22.96	40.3	**0.067**	0.007	0.0081	0.0008
G-LEAD_05_192h_B2_DIG	5	193	14.3	4.3	0.0315	0.0004	0.106	22.96	40.3	0.061	0.006	0.0087	0.0009

1	2	3	4	5	6	7	8	9	10	11*	12	13	14
**SAMPLE ID**	**SUBJECT**	**time**	**Total Pb in sample (µg/L)**	**2 *SD***	**$^{204}Pb/^{208}Pb$**	**1 *SD***	**molar fraction ^{204}Pb-tracer (%)**	**BMI**	**HCT (%)**	**molar fraction ^{204}Pb-tracer (%) (normalized to HCT/BMI)***	***u* (10%)**	**absolute amount of ^{204}Pb from tracer (µg/L) - normalized to HCT/BMI**	***u* (10%)**
G-LEAD_06_BL_B1_DIG	6	0	15.5	4.6	0.0270	0.0004	0.000	26.87	39.8	0.000	0.000	0.0000	0.0000
G-LEAD_06_4h_B1_DIG	6	4	19.4	5.8	0.0274	0.0007	0.022	26.87	39.8	0.015	0.001	0.0028	0.0003
G-LEAD_06_8h_B1_DIG	6	8	14.6	4.4	0.0281	0.0007	0.054	26.87	39.8	0.037	0.004	0.0054	0.0005
G-LEAD_06_12h_B1_DIG	6	12	19.3	5.8	0.0282	0.0005	0.063	26.87	39.8	0.042	0.004	0.0082	0.0008
G-LEAD_06_24h_B1_DIG	6	24	16.0	4.8	0.0281	0.0007	0.056	26.87	39.8	0.038	0.004	0.0060	0.0006
G-LEAD_06_48h_B1_DIG	6	48	16.9	5.1	0.0285	0.0007	**0.076**	26.87	39.8	**0.051**	0.005	0.0087	0.0009
G-LEAD_06_192h_B1_DIG	6	193	19.5	5.9	0.0283	0.0006	0.066	26.87	39.8	0.045	0.004	0.0087	0.0009
G-LEAD_09_BL_B1_DIG	9	0	13.7	4.1	0.0296	0.0005	0.000	20.68	38.9	0.000	0.000	0.0000	0.0000
G-LEAD_09_4h_B1_DIG	9	4	14.1	4.2	0.0317	0.0005	0.106	20.68	38.9	0.057	0.006	0.0080	0.0008
G-LEAD_09_8h_B1_DIG	9	8	13.0	3.9	0.0335	0.0011	0.201	20.68	38.9	0.107	0.011	0.0140	0.0014
G-LEAD_09_12h_B1_DIG	9	12	11.8	3.5	0.0334	0.0009	0.220	20.68	38.9	0.117	0.012	0.0138	0.0014
G-LEAD_09_24h_B1_DIG	9	24	14.3	4.3	0.0346	0.0011	0.255	20.68	38.9	0.136	0.014	0.0193	0.0019
G-LEAD_09_48h_B1_DIG	9	48	12.0	3.6	0.0353	0.0007	**0.291**	20.68	38.9	**0.155**	0.015	0.0186	0.0019
G-LEAD_09_192h_B1_DIG	9	197	14.1	4.2	0.0344	0.0006	0.244	20.68	38.9	0.130	0.013	0.0183	0.0018
G-LEAD_10_BL_B1_DIG	10	0	16.0	4.8	0.0299	0.0005	0.000	22.02	41	0.000	0.000	0.0000	0.0000
G-LEAD_10_4h_B1_DIG	10	4	12.1	3.6	0.0300	0.0005	0.005	22.02	41	0.003	0.000	0.0003	0.0000
G-LEAD_10_24h_B1_DIG	10	24	11.8	3.5	0.0319	0.0006	**0.102**	22.02	41	**0.055**	0.005	0.0065	0.0006
G-LEAD_10_48h_B1_DIG	10	49	12.3	3.7	0.0317	0.0006	0.092	22.02	41	0.049	0.005	0.0061	0.0006
G-LEAD_10_192h_B1_DIG	10	192	12.0	3.6	0.0318	0.0005	0.092	22.02	41	0.050	0.005	0.0059	0.0006
G-LEAD_11_BL_B1_DIG	11	0	11.9	3.6	0.0283	0.0004	0.000	22.31	36.3	0.000	0.000	0.0000	0.0000
G-LEAD_11_4h_B1_DIG	11	4	10.7	3.2	0.0355	0.0008	0.379	22.31	36.3	0.233	0.023	0.0248	0.0025
G-LEAD_11_24h_B1_DIG	11	24	10.9	3.3	0.0486	0.0011	1.058	22.31	36.3	0.650	0.065	0.0708	0.0071
G-LEAD_11_48h_B1_DIG	11	48	10.6	3.2	0.0506	0.0006	**1.160**	22.31	36.3	**0.713**	0.071	0.0758	0.0076
G-LEAD_11_192h_B1_DIG	11	197	9.2	2.8	0.0495	0.0012	1.111	22.31	36.3	0.683	0.068	0.0629	0.0063
G-LEAD_12_BL_B1_DIG	12	0	9.2	2.8	0.0308	0.0010	0.000	23.39	42.4	0.000	0.000	0.0000	0.0000
G-LEAD_12_4h_B1_DIG	12	4	8.4	2.5	0.0315	0.0006	0.041	23.39	42.4	0.023	0.002	0.0019	0.0002
G-LEAD_12_24h_B1_DIG	12	24	9.0	2.7	0.0336	0.0007	0.135	23.39	42.4	0.075	0.007	0.0067	0.0007
G-LEAD_12_48h_B1_DIG	12	48	9.6	2.9	0.0340	0.0008	**0.166**	23.39	42.4	**0.092**	0.009	0.0088	0.0009
G-LEAD_12_192h_B1_DIG	12	198	9.3	2.8	0.0325	0.0007	0.095	23.39	42.4	0.052	0.005	0.0049	0.0005

1	2	3	4	5	6	7	8	9	10	11*	12	13	14
**SAMPLE ID**	**SUBJECT**	**time**	**Total Pb in sample (µg/L)**	**2 *SD***	**$^{204}Pb/^{208}Pb$**	**1 *SD***	**molar fraction ^{204}Pb-tracer (%)**	**BMI**	**HCT (%)**	**molar fraction ^{204}Pb-tracer (%) (normalized to HCT/BMI)***	***u* (10%)**	**absolute amount of ^{204}Pb from tracer (µg/L) - normalized to HCT/BMI**	***u* (10%)**
G-LEAD_13_BL_B1_DIG	13	0	11.7	3.5	0.0338	0.0009	0.000	17.86	38.8	0.000	0.000	0.0000	0.0000
G-LEAD_13_4h_B1_DIG	13	4	10.4	3.1	0.0359	0.0007	0.103	17.86	38.8	0.047	0.005	0.0049	0.0005
G-LEAD_13_24h_B1_DIG	13	24	10.6	3.2	0.0364	0.0008	0.136	17.86	38.8	0.062	0.006	0.0066	0.0007
G-LEAD_13_48h_B1_DIG	13	49	9.5	2.9	0.0368	0.0008	**0.152**	17.86	38.8	**0.070**	0.007	0.0067	0.0007
G-LEAD_13_192h_B1_DIG	13	195	9.7	2.9	0.0360	0.0006	0.112	17.86	38.8	0.052	0.005	0.0050	0.0005
G-LEAD_14_BL_B1_DIG	14	0	24.6	7.4	0.0266	0.0005	0.000	20.91	43.2	0.000	0.000	0.0000	0.0000
G-LEAD_14_4h_B1_DIG	14	4	24.9	7.5	0.0324	0.0005	0.304	20.91	43.2	0.147	0.015	0.0367	0.0037
G-LEAD_14_24h_B1_DIG	14	24	27.6	8.3	0.0387	0.0006	0.639	20.91	43.2	0.309	0.031	0.0854	0.0085
G-LEAD_14_48h_B1_DIG	14	49	25.8	7.7	0.0402	0.0005	**0.710**	20.91	43.2	**0.344**	0.034	0.0887	0.0089
G-LEAD_14_192h_B1_DIG	14	193	27.6	8.3	0.0395	0.0006	0.679	20.91	43.2	0.329	0.033	0.0908	0.0091
G-LEAD_15_BL_B1_DIG	15	0	11.8	3.5	0.0289	0.0007	0.000	23.03	38.2	0.000	0.000	0.0000	0.0000
G-LEAD_15_4h_B1_DIG	15	4	11.6	3.5	0.0297	0.0004	0.040	23.03	38.2	0.024	0.002	0.0028	0.0003
G-LEAD_15_24h_B1_DIG	15	24	11.1	3.3	0.0313	0.0009	**0.125**	23.03	38.2	**0.075**	0.008	0.0083	0.0008
G-LEAD_15_48h_B1_DIG	15	49	11.5	3.5	0.0311	0.0006	0.114	23.03	38.2	0.069	0.007	0.0079	0.0008
G-LEAD_15_192h_B1_DIG	15	197	11.4	3.4	0.0308	0.0007	0.103	23.03	38.2	0.062	0.006	0.0071	0.0007
G-LEAD_16_BL_B1_DIG	16	0	39.4	11.8	0.0264	0.0003	0.000	22.28	40.2	0.000	0.000	0.0000	0.0000
G-LEAD_16_4h_B1_DIG	16	4	39.1	11.7	0.0266	0.0004	0.013	22.28	40.2	0.007	0.001	0.0027	0.0003
G-LEAD_16_24h_B1_DIG	16	25	40.9	12.3	0.0273	0.0004	0.041	22.28	40.2	0.023	0.002	0.0094	0.0009
G-LEAD_16_48h_B1_DIG	16	48	41.7	12.5	0.0270	0.0004	0.030	22.28	40.2	0.017	0.002	0.0069	0.0007
G-LEAD_16_192h_B1_DIG	16	196	42.3	12.7	0.0272	0.0005	**0.043**	22.28	40.2	**0.024**	0.002	0.0101	0.0010
G-LEAD_17_BL_B1_DIG	17	0	21.0	6.3	0.0290	0.0007	0.000	26.85	44.5	0.000	0.000	0.0000	0.0000
G-LEAD_17_4h_B1_DIG	17	4	19.1	5.7	0.0300	0.0007	0.055	26.85	44.5	0.033	0.003	0.0064	0.0006
G-LEAD_17_24h_B1_DIG	17	24	19.8	5.9	0.0309	0.0005	0.101	26.85	44.5	0.061	0.006	0.0121	0.0012
G-LEAD_17_48h_B1_DIG	17	50	20.7	6.2	0.0311	0.0008	**0.111**	26.85	44.5	**0.067**	0.007	0.0138	0.0014
G-LEAD_17_192h_B1_DIG	17	197	31.4	9.4	0.0293	0.0006	0.019	26.85	44.5	0.011	0.001	0.0036	0.0004
G-LEAD_18_BL_B1_DIG	18	0	15.0	4.5	0.0280	0.0005	0.000	22.59	42.4	0.000	0.000	0.0000	0.0000
G-LEAD_18_4h_B1_DIG	18	4	14.5	4.3	0.0339	0.0006	0.309	22.59	42.4	0.164	0.016	0.0238	0.0024
G-LEAD_18_24h_B1_DIG	18	24	15.3	4.6	0.0419	0.0009	0.724	22.59	42.4	0.385	0.039	0.0588	0.0059
G-LEAD_18_48h_B1_DIG	18	49	15.5	4.6	0.0431	0.0009	0.788	22.59	42.4	0.420	0.042	0.0649	0.0065

1	2	3	4	5	6	7	8	9	10	11*	12	13	14
**SAMPLE ID**	**SUBJECT**	**time**	**Total Pb in sample (µg/L)**	**2 *SD***	**$^{204}Pb/^{208}Pb$**	**1 *SD***	**molar fraction ^{204}Pb-tracer (%)**	**BMI**	**HCT (%)**	**molar fraction ^{204}Pb-tracer (%) (normalized to HCT/BMI)***	***u* (10%)**	**absolute amount of ^{204}Pb from tracer (µg/L) - normalized to HCT/BMI**	***u* (10%)**
G-LEAD_18_192h_B1_DIG	18	193	15.1	4.5	0.0435	0.0006	**0.807**	22.59	42.4	**0.430**	0.043	0.0648	0.0065
G-LEAD_19_BL_B1_DIG	19	0	6.0	1.8	0.0301	0.0011	0.000	25.99	43.8	0.000	0.000	0.0000	0.0000
G-LEAD_19_4h_B1_DIG	19	4	5.9	1.8	0.0316	0.0010	0.078	25.99	43.8	0.047	0.005	0.0027	0.0003
G-LEAD_19_24h_B1_DIG	19	24	6.3	1.9	0.0332	0.0008	0.164	25.99	43.8	0.097	0.010	0.0061	0.0006
G-LEAD_19_48h_B1_DIG	19	48	6.1	1.8	0.0336	0.0014	**0.190**	25.99	43.8	**0.112**	0.011	0.0068	0.0007
G-LEAD_19_192h_B1_DIG	19	195	6.5	2.0	0.0335	0.0007	0.181	25.99	43.8	0.108	0.011	0.0070	0.0007
G-LEAD_21_BL_B1_DIG	21	0	26.4	7.9	0.0260	0.0003	0.000	24.62	42.8	0.000	0.000	0.0000	0.0000
G-LEAD_21_4h_B1_DIG	21	4	25.1	7.5	0.0316	0.0004	0.294	24.62	42.8	0.169	0.017	0.0424	0.0042
G-LEAD_21_24h_B1_DIG	21	24	28.0	8.4	0.0374	0.0004	0.599	24.62	42.8	0.345	0.034	0.0963	0.0096
G-LEAD_21_48h_B1_DIG	21	48	26.6	8.0	0.0389	0.0006	0.678	24.62	42.8	0.390	0.039	0.1037	0.0104
G-LEAD_21_192h_B1_DIG	21	192	25.4	7.6	0.0391	0.0007	**0.683**	24.62	42.8	**0.393**	0.039	0.0998	0.0100
G-LEAD_22_BL_B1_DIG	22	0	10.8	3.2	0.0296	0.0008	0.000	20.45	37.2	0.000	0.000	0.0000	0.0000
G-LEAD_22_4h_B1_DIG	22	4	10.9	3.3	0.0311	0.0004	0.081	20.45	37.2	0.045	0.004	0.0049	0.0005
G-LEAD_22_24h_B1_DIG	22	24	10.4	3.1	0.0336	0.0025	**0.212**	20.45	37.2	**0.116**	0.012	0.0121	0.0012
n/a											0.000	0.0000	0.0000
G-LEAD_22_192h_B1_DIG	22	194	10.6	3.2	0.0336	0.0010	0.208	20.45	37.2	0.115	0.011	0.0122	0.0012
G-LEAD_23_BL_B1_DIG	23	0	9.0	2.7	0.0309	0.0008	0.000	20.66	43.6	0.000	0.000	0.0000	0.0000
G-LEAD_23_4h_B1_DIG	23	4	8.6	2.6	0.0323	0.0007	0.073	20.66	43.6	0.035	0.003	0.0030	0.0003
G-LEAD_23_24h_B1_DIG	23	24	9.5	2.8	0.0337	0.0011	0.150	20.66	43.6	0.071	0.007	0.0067	0.0007
G-LEAD_23_48h_B1_DIG	23	49	9.1	2.7	0.0343	0.0009	**0.176**	20.66	43.6	**0.084**	0.008	0.0076	0.0008
G-LEAD_23_192h_B1_DIG	23	192	7.9	2.4	0.0337	0.0003	0.151	20.66	43.6	0.072	0.007	0.0057	0.0006
G-LEAD_24_BL_B1_DIG	24	0	4.3	1.3	0.0279	0.0008	0.000	22.85	39.7	0.000	0.000	0.0000	0.0000
G-LEAD_24_4h_B1_DIG	24	4	4.7	1.4	0.0285	0.0011	0.028	22.85	39.7	0.016	0.002	0.0008	0.0001
G-LEAD_24_24h_B1_DIG	24	24	4.8	1.4	0.0296	0.0005	0.088	22.85	39.7	0.051	0.005	0.0024	0.0002
G-LEAD_24_48h_B1_DIG	24	49	4.4	1.3	0.0299	0.0008	**0.104**	22.85	39.7	**0.060**	0.006	0.0027	0.0003
G-LEAD_24_192h_B1_DIG	24	193	4.5	1.3	0.0293	0.0009	0.073	22.85	39.7	0.042	0.004	0.0019	0.0002
G-LEAD_27_BL_B1_DIG	27	0	51.3	15.4	0.0268	0.0002	0.000	19.58	42.9	0.000	0.000	0.0000	0.0000
G-LEAD_27_4h_B1_DIG	27	4	50.9	15.3	0.0289	0.0003	0.117	19.58	42.9	0.053	0.005	0.0271	0.0027

1	2	3	4	5	6	7	8	9	10	11*	12	13	14
**SAMPLE ID**	**SUBJECT**	**time**	**Total Pb in sample (µg/L)**	**2 *SD***	**$^{204}Pb/^{208}Pb$**	**1 *SD***	**molar fraction ^{204}Pb-tracer (%)**	**BMI**	**HCT (%)**	**molar fraction ^{204}Pb-tracer (%) (normalized to HCT/BMI)***	***u* (10%)**	**absolute amount of ^{204}Pb from tracer (µg/L) - normalized to HCT/BMI**	***u* (10%)**
G-LEAD_27_24h_B1_DIG	27	24	49.7	14.9	0.0315	0.0003	0.252	19.58	42.9	0.115	0.011	0.0572	0.0057
G-LEAD_27_48h_B1_DIG	27	49	51.1	15.3	0.0321	0.0004	**0.286**	19.58	42.9	**0.130**	0.013	0.0666	0.0067
G-LEAD_27_192h_B1_DIG	27	195	52.1	15.6	0.0316	0.0003	0.253	19.58	42.9	0.116	0.012	0.0602	0.0060
G-LEAD_28_BL_B1_DIG	28	0	19.5	5.9	0.0267	0.0005	0.000	20.16	43.4	0.000	0.000	0.0000	0.0000
G-LEAD_28_4h_B1_DIG	28	4	18.5	5.6	0.0271	0.0004	0.018	20.16	43.4	0.008	0.001	0.0015	0.0002
G-LEAD_28_24h_B1_DIG	28	24	18.5	5.6	0.0280	0.0005	0.067	20.16	43.4	0.031	0.003	0.0057	0.0006
G-LEAD_28_48h_B1_DIG	28	48	18.8	5.6	0.0281	0.0004	**0.074**	20.16	43.4	**0.034**	0.003	0.0065	0.0006
G-LEAD_28_192h_B1_DIG	28	192	20.4	6.1	0.0280	0.0004	0.070	20.16	43.4	0.033	0.003	0.0067	0.0007
G-LEAD_29_BL_B1_DIG	29	0	15.4	4.6	0.0288	0.0008	0.000	20.76	39.2	0.000	0.000	0.0000	0.0000
G-LEAD_29_4h_B1_DIG	29	4	15.6	4.7	0.0389	0.0006	0.529	20.76	39.2	0.280	0.028	0.0438	0.0044
G-LEAD_29_24h_B1_DIG	29	24	16.3	4.9	0.0488	0.0008	1.043	20.76	39.2	0.552	0.055	0.0901	0.0090
G-LEAD_29_48h_B1_DIG	29	48	17.4	5.2	0.0512	0.0008	**1.165**	20.76	39.2	**0.617**	0.062	0.1075	0.0108
G-LEAD_29_192h_B1_DIG	29	192	16.5	5.0	0.0497	0.0010	1.087	20.76	39.2	0.576	0.058	0.0950	0.0095
G-LEAD_31_BL_B1_DIG	31	0	24.0	7.2	0.0267	0.0004	0.000	26.25	44.4	0.000	0.000	0.0000	0.0000
G-LEAD_31_4h_B1_DIG	31	4	24.3	7.3	0.0273	0.0005	0.032	26.25	44.4	0.019	0.002	0.0046	0.0005
G-LEAD_31_24h_B1_DIG	31	24	24.6	7.4	0.0279	0.0005	**0.062**	26.25	44.4	**0.037**	0.004	0.0091	0.0009
G-LEAD_31_48h_B1_DIG	31	48	23.2	6.9	0.0279	0.0005	0.062	26.25	44.4	0.037	0.004	0.0085	0.0009
G-LEAD_31_192h_B1_DIG	31	195	24.0	7.2	0.0278	0.0006	0.056	26.25	44.4	0.033	0.003	0.0080	0.0008
G-LEAD_32_BL_B1_DIG	32	0	11.8	3.5	0.0277	0.0008	0.000	21.6	43.2	0.000	0.000	0.0000	0.0000
G-LEAD_32_4h_B1_DIG	32	4	11.7	3.5	0.0339	0.0006	0.326	21.6	43.2	0.163	0.016	0.0191	0.0019
G-LEAD_32_24h_B1_DIG	32	24	11.7	3.5	0.0421	0.0010	0.759	21.6	43.2	0.380	0.038	0.0446	0.0045
G-LEAD_32_48h_B1_DIG	32	48	11.7	3.5	0.0429	0.0008	0.799	21.6	43.2	0.400	0.040	0.0468	0.0047
G-LEAD_32_192h_B1_DIG	32	194	12.9	3.9	0.0437	0.0007	**0.836**	21.6	43.2	**0.418**	0.042	0.0540	0.0054
G-LEAD_33_BL_B1_DIG	33	0	5.4	1.6	0.0316	0.0009	0.000	21.05	36.8	0.000	0.000	0.0000	0.0000
G-LEAD_33_4h_B1_DIG	33	4	5.4	1.6	0.0322	0.0010	0.031	21.05	36.8	0.018	0.002	0.0009	0.0001
G-LEAD_33_24h_B1_DIG	33	24	5.2	1.6	0.0336	0.0010	0.100	21.05	36.8	0.057	0.006	0.0030	0.0003
G-LEAD_33_48h_B1_DIG	33	48	5.4	1.6	0.0340	0.0006	**0.122**	21.05	36.8	**0.070**	0.007	0.0038	0.0004
G-LEAD_33_192h_B1_DIG	33	193	5.8	1.7	0.0333	0.0007	0.084	21.05	36.8	0.048	0.005	0.0028	0.0003

1	2	3	4	5	6	7	8	9	10	11*	12	13	14
**SAMPLE ID**	**SUBJECT**	**time**	**Total Pb in sample (µg/L)**	**2 *SD***	**^{204}Pb/^{208}Pb**	**1 *SD***	**molar fraction ^{204}Pb-tracer (%)**	**BMI**	**HCT (%)**	**molar fraction ^{204}Pb-tracer (%) (normalized to HCT/BMI)***	***u* (10%)**	**absolute amount of ^{204}Pb from tracer (µg/L) - normalized to HCT/BMI**	***u* (10%)**
G-LEAD_34_BL_B1_DIG	34	0	5.1	1.5	0.0380	0.0007	0.000	22.16	42.3	0.000	0.000	0.0000	0.0000
G-LEAD_34_4h_B1_DIG	34	4	5.1	1.5	0.0380	0.0013	0.009	22.16	42.3	0.005	0.000	0.0002	0.0000
G-LEAD_34_24h_B1_DIG	34	24	4.9	1.5	0.0385	0.0012	0.029	22.16	42.3	0.015	0.002	0.0007	0.0001
G-LEAD_34_48h_B1_DIG	34	48	5.3	1.6	0.0395	0.0006	0.084	22.16	42.3	0.044	0.004	0.0023	0.0002
G-LEAD_34_192h_B1_DIG	34	192	5.0	1.5	0.0410	0.0011	**0.157**	22.16	42.3	**0.082**	0.008	0.0041	0.0004
G-LEAD_35_BL_B1_DIG	35	0	15.6	4.7	0.0275	0.0005	0.000	21.33	45.7	0.000	0.000	0.0000	0.0000
G-LEAD_35_4h_B1_DIG	35	4	14.7	4.4	0.0281	0.0005	0.031	21.33	45.7	0.014	0.001	0.0021	0.0002
G-LEAD_35_24h_B1_DIG	35	24	14.9	4.5	0.0291	0.0004	0.084	21.33	45.7	0.039	0.004	0.0058	0.0006
G-LEAD_35_48h_B1_DIG	35	48	15.0	4.5	0.0292	0.0006	0.086	21.33	45.7	0.040	0.004	0.0060	0.0006
G-LEAD_35_192h_B1_DIG	35	193	15.5	4.6	0.0293	0.0005	**0.095**	21.33	45.7	**0.045**	0.004	0.0069	0.0007
G-LEAD_37_BL_B1_DIG	37	0	9.7	2.9	0.0266	0.0006	0.000	21.3	37.8	0.000	0.000	0.0000	0.0000
G-LEAD_37_4h_B1_DIG	37	4	9.8	2.9	0.0377	0.0005	0.578	21.3	37.8	0.326	0.033	0.0319	0.0032
G-LEAD_37_24h_B1_DIG	37	24	9.3	2.8	0.0557	0.0007	1.513	21.3	37.8	0.852	0.085	0.0793	0.0079
G-LEAD_37_48h_B1_DIG	37	48	9.1	2.7	0.0588	0.0009	**1.678**	21.3	37.8	**0.945**	0.095	0.0864	0.0086
G-LEAD_37_192h_B1_DIG	37	196	10.1	3.0	0.0574	0.0007	1.600	21.3	37.8	0.902	0.090	0.0908	0.0091
G-LEAD_38_BL_B1_DIG	38	0	8.7	2.6	0.0287	0.0003	0.000	23.81	40.3	0.000	0.000	0.0000	0.0000
G-LEAD_38_4h_B1_DIG	38	4	8.8	2.6	0.0312	0.0001	0.131	23.81	40.3	0.077	0.008	0.0068	0.0007
G-LEAD_38_24h_B1_DIG	38	24	8.4	2.5	0.0336	0.0012	0.261	23.81	40.3	0.154	0.015	0.0129	0.0013
G-LEAD_38_48h_B1_DIG	38	49	8.7	2.6	0.0344	0.0008	0.299	23.81	40.3	0.176	0.018	0.0154	0.0015
G-LEAD_38_192h_B1_DIG	38	193	9.8	2.9	0.0349	0.0007	**0.325**	23.81	40.3	**0.192**	0.019	0.0188	0.0019
G-LEAD_39_BL_B1_DIG	39	0	15.0	4.5	0.0277	0.0006	0.000	24.05	44.6	0.000	0.000	0.0000	0.0000
G-LEAD_39_4h_B1_DIG	39	4	15.4	4.6	0.0288	0.0007	0.053	24.05	44.6	0.029	0.003	0.0044	0.0004
G-LEAD_39_24h_B1_DIG	39	24	14.5	4.4	0.0294	0.0007	0.085	24.05	44.6	0.046	0.005	0.0066	0.0007
G-LEAD_39_48h_B1_DIG	39	49	15.0	4.5	0.0297	0.0004	0.101	24.05	44.6	0.054	0.005	0.0082	0.0008
G-LEAD_39_192h_B1_DIG	39	193	14.6	4.4	0.0297	0.0007	**0.101**	24.05	44.6	**0.055**	0.005	0.0080	0.0008
G-LEAD_40_BL_B1_DIG	40	0	19.8	5.9	0.0267	0.0006	0.000	19.15	41.4	0.000	0.000	0.0000	0.0000
G-LEAD_40_4h_B1_DIG	40	4	19.6	5.9	0.0335	0.0005	0.355	19.15	41.4	0.164	0.016	0.0322	0.0032
G-LEAD_40_24h_B1_DIG	40	24	24.9	7.5	0.0397	0.0008	0.682	19.15	41.4	0.316	0.032	0.0785	0.0078

1	2	3	4	5	6	7	8	9	10	11*	12	13	14
**SAMPLE ID**	**SUBJECT**	**time**	**Total Pb in sample (µg/L)**	**2 *SD***	**$^{204}Pb/^{208}Pb$**	**1 *SD***	**molar fraction ^{204}Pb-tracer (%)**	**BMI**	**HCT (%)**	**molar fraction ^{204}Pb-tracer (%) (normalized to HCT/BMI)***	***u* (10%)**	**absolute amount of ^{204}Pb from tracer (µg/L) - normalized to HCT/BMI**	***u* (10%)**
G-LEAD_40_48h_B1_DIG	40	49	19.7	5.9	0.0403	0.0007	0.717	19.15	41.4	0.331	0.033	0.0653	0.0065
G-LEAD_40_192h_B1_DIG	40	197	18.8	5.6	0.0404	0.0008	**0.721**	19.15	41.4	**0.333**	0.033	0.0625	0.0063
G-LEAD_41_BL_B1_DIG	41	0	9.6	2.9	0.0272	0.0008	0.000	19.13	38.9	0.000	0.000	0.0000	0.0000
G-LEAD_41_4h_B1_DIG	41	4	9.8	2.9	0.0278	0.0005	0.030	19.13	38.9	0.015	0.001	0.0014	0.0001
G-LEAD_41_24h_B1_DIG	41	24	9.7	2.9	0.0291	0.0007	0.101	19.13	38.9	0.049	0.005	0.0048	0.0005
G-LEAD_41_48h_B1_DIG	41	48	9.7	2.9	0.0292	0.0005	0.107	19.13	38.9	0.052	0.005	0.0051	0.0005
G-LEAD_41_192h_B1_DIG	41	194	10.4	3.1	0.0293	0.0006	**0.112**	19.13	38.9	**0.055**	0.006	0.0057	0.0006
G-LEAD_42_BL_B1_DIG	42	0	29.7	8.9	0.0280	0.0005	0.000	23.74	40.2	0.000	0.000	0.0000	0.0000
G-LEAD_42_4h_B1_DIG	42	4	30.1	9.0	0.0283	0.0003	0.016	23.74	40.2	0.009	0.001	0.0028	0.0003
G-LEAD_42_24h_B1_DIG	42	24	27.2	8.2	0.0287	0.0003	0.037	23.74	40.2	0.022	0.002	0.0059	0.0006
G-LEAD_42_48h_B1_DIG	42	48	29.5	8.9	0.0288	0.0005	**0.038**	23.74	40.2	**0.022**	0.002	0.0066	0.0007
G-LEAD_42_192h_B1_DIG	42	194	16.7	5.0	0.0286	0.0007	0.032	23.74	40.2	0.019	0.002	0.0032	0.0003
G-LEAD_43_BL_B1_DIG	43	0	10.4	3.1	0.0271	0.0007	0.000	23.66	38	0.000	0.000	0.0000	0.0000
G-LEAD_43_4h_B1_DIG	43	4	10.8	3.2	0.0369	0.0010	0.513	23.66	38	0.319	0.032	0.0345	0.0034
G-LEAD_43_24h_B1_DIG	43	24	10.3	3.1	0.0542	0.0013	1.413	23.66	38	0.880	0.088	0.0902	0.0090
G-LEAD_43_48h_B1_DIG	43	48	11.9	3.6	0.0571	0.0013	**1.560**	23.66	38	**0.971**	0.097	0.1157	0.0116
G-LEAD_43_192h_B1_DIG	43	192	11.7	3.5	0.0543	0.0014	1.418	23.66	38	0.883	0.088	0.1035	0.0104
G-LEAD_45_BL_B1_DIG	45	0	47.3	14.2	0.0261	0.0004	0.000	22.49	39.5	0.000	0.000	0.0000	0.0000
G-LEAD_45_4h_B1_DIG	45	4	20.5	6.1	0.0271	0.0006	0.052	22.49	39.5	0.030	0.003	0.0061	0.0006
G-LEAD_45_24h_B1_DIG	45	24	20.9	6.3	0.0273	0.0005	0.064	22.49	39.5	0.036	0.004	0.0076	0.0008
G-LEAD_45_48h_B1_DIG	45	48	21.4	6.4	0.0276	0.0004	**0.078**	22.49	39.5	**0.045**	0.004	0.0096	0.0010
G-LEAD_45_192h_B1_DIG	45	194	21.4	6.4	0.0274	0.0006	0.069	22.49	39.5	0.040	0.004	0.0084	0.0008
G-LEAD_46_BL_B1_DIG	46	0	38.7	11.6	0.0268	0.0004	0.000	20.37	42.5	0.000	0.000	0.0000	0.0000
G-LEAD_46_4h_B1_DIG	46	4	38.8	11.6	0.0299	0.0003	0.163	20.37	42.5	0.078	0.008	0.0303	0.0030
G-LEAD_46_24h_B1_DIG	46	24	37.7	11.3	0.0328	0.0004	0.319	20.37	42.5	0.153	0.015	0.0577	0.0058
G-LEAD_46_48h_B1_DIG	46	48	38.5	11.5	0.0332	0.0005	**0.341**	20.37	42.5	**0.164**	0.016	0.0629	0.0063
G-LEAD_46_192h_B1_DIG	46	196	40.6	12.2	0.0329	0.0005	0.324	20.37	42.5	0.155	0.016	0.0631	0.0063

1	2	3	4	5	6	7	8	9	10	11*	12	13	14
**SAMPLE ID**	**SUBJECT**	**time**	**Total Pb in sample (µg/L)**	**2 *SD***	**^{204}Pb/^{208}Pb**	**1 *SD***	**molar fraction ^{204}Pb-tracer (%)**	**BMI**	**HCT (%)**	**molar fraction ^{204}Pb-tracer (%) (normalized to HCT/BMI)***	***u* (10%)**	**absolute amount of ^{204}Pb from tracer (µg/L) - normalized to HCT/BMI**	***u* (10%)**
G-LEAD_47_BL_B1_DIG	47	0	13.8	4.1	0.0265	0.0005	0.000	23.95	38.4	0.000	0.000	0.0000	0.0000
G-LEAD_47_4h_B1_DIG	47	4	14.4	4.3	0.0273	0.0007	0.044	23.95	38.4	0.027	0.003	0.0040	0.0004
G-LEAD_47_24h_B1_DIG	47	24	14.2	4.3	0.0281	0.0006	0.086	23.95	38.4	0.054	0.005	0.0077	0.0008
G-LEAD_47_48h_B1_DIG	47	48	14.6	4.4	0.0289	0.0004	**0.131**	23.95	38.4	**0.081**	0.008	0.0119	0.0012
G-LEAD_47_192h_B1_DIG	47	197	17.0	5.1	0.0286	0.0004	0.110	23.95	38.4	0.069	0.007	0.0117	0.0012
G-LEAD_48_BL_B1_DIG	48	0	14.8	4.4	0.0282	0.0005	0.000	21.87	41.4	0.000	0.000	0.0000	0.0000
G-LEAD_48_4h_B1_DIG	48	4	14.3	4.3	0.0291	0.0007	0.043	21.87	41.4	0.023	0.002	0.0033	0.0003
G-LEAD_48_24h_B1_DIG	48	24	14.6	4.4	0.0299	0.0005	0.084	21.87	41.4	0.045	0.004	0.0065	0.0006
G-LEAD_48_48h_B1_DIG	48	48	15.4	4.6	0.0298	0.0006	0.080	21.87	41.4	0.042	0.004	0.0065	0.0007
G-LEAD_48_192h_B1_DIG	48	196	14.3	4.3	0.0302	0.0005	**0.102**	21.87	41.4	**0.054**	0.005	0.0077	0.0008
G-LEAD_49_BL_B1_DIG	49	0	7.1	2.1	0.0265	0.0005	0.000	21.63	35.5	0.000	0.000	0.0000	0.0000
G-LEAD_49_4h_B1_DIG	49	4	6.5	1.9	0.0325	0.0009	0.311	21.63	35.5	0.190	0.019	0.0123	0.0012
G-LEAD_49_24h_B1_DIG	49	24	6.5	1.9	0.0487	0.0007	1.155	21.63	35.5	0.703	0.070	0.0454	0.0045
G-LEAD_49_48h_B1_DIG	49	49	7.7	2.3	0.0494	0.0011	1.190	21.63	35.5	0.725	0.073	0.0558	0.0056
G-LEAD_49_192h_B1_DIG	49	197	7.2	2.2	0.0498	0.0013	**1.212**	21.63	35.5	**0.739**	0.074	0.0532	0.0053
G-LEAD_50_BL_B1_DIG	50	0	19.3	5.8	0.0288	0.0005	0.000	24.22	47	0.000	0.000	0.0000	0.0000
G-LEAD_50_4h_B1_DIG	50	4	18.7	5.6	0.0289	0.0004	0.006	24.22	47	0.003	0.000	0.0006	0.0001
G-LEAD_50_24h_B1_DIG	50	24	17.7	5.3	0.0292	0.0003	0.023	24.22	47	0.012	0.001	0.0021	0.0002
n/a		48											
G-LEAD_50_192h_B1_DIG	50	196	16.6	5.0	0.0295	0.0006	**0.041**	24.22	47	**0.021**	0.002	0.0035	0.0004
G-LEAD_51_BL_B1_DIG	51	0	12.0	3.6	0.0276	0.0008	0.000	20.11	41.5	0.000	0.000	0.0000	0.0000
G-LEAD_51_4h_B1_DIG	51	4	12.2	3.7	0.0279	0.0006	0.017	20.11	41.5	0.008	0.001	0.0010	0.0001
G-LEAD_51_24h_B1_DIG	51	24	10.8	3.3	0.0288	0.0006	0.061	20.11	41.5	0.029	0.003	0.0032	0.0003
G-LEAD_51_48h_B1_DIG	51	48	12.4	3.7	0.0289	0.0007	**0.070**	20.11	41.5	**0.034**	0.003	0.0042	0.0004
G-LEAD_51_192h_B1_DIG	51	197	15.8	4.7	0.0283	0.0006	0.036	20.11	41.5	0.017	0.002	0.0027	0.0003

Figure S01-S42 ^{204}Pb enrichment kinetics in 42 subjects as function of time

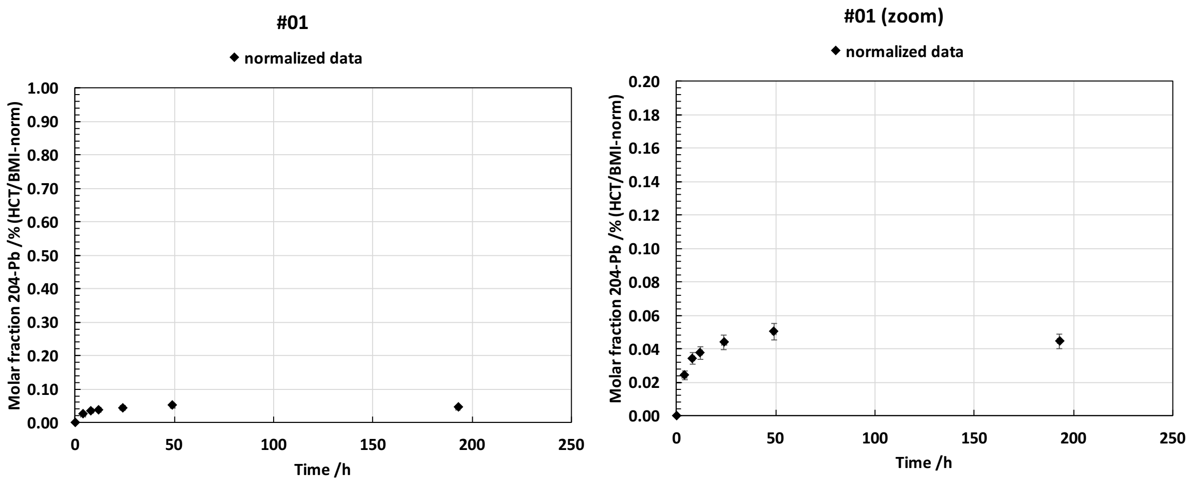

Figure S01 Subject-01: ^{204}Pb-molar fraction in Pb normalized to HCT/BMI.

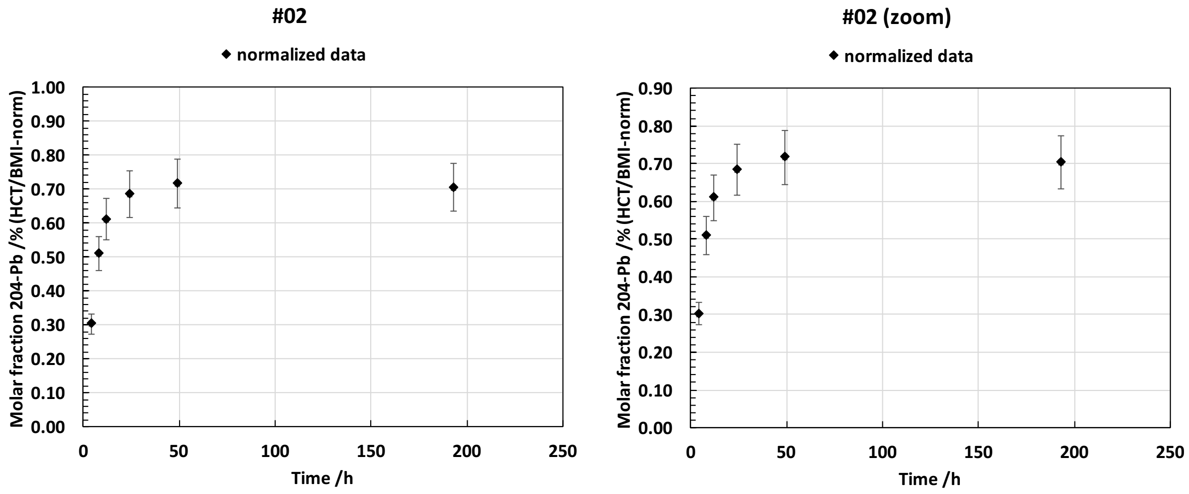

Figure S02 Subject-02: ^{204}Pb-molar fraction in Pb normalized to HCT/BMI.

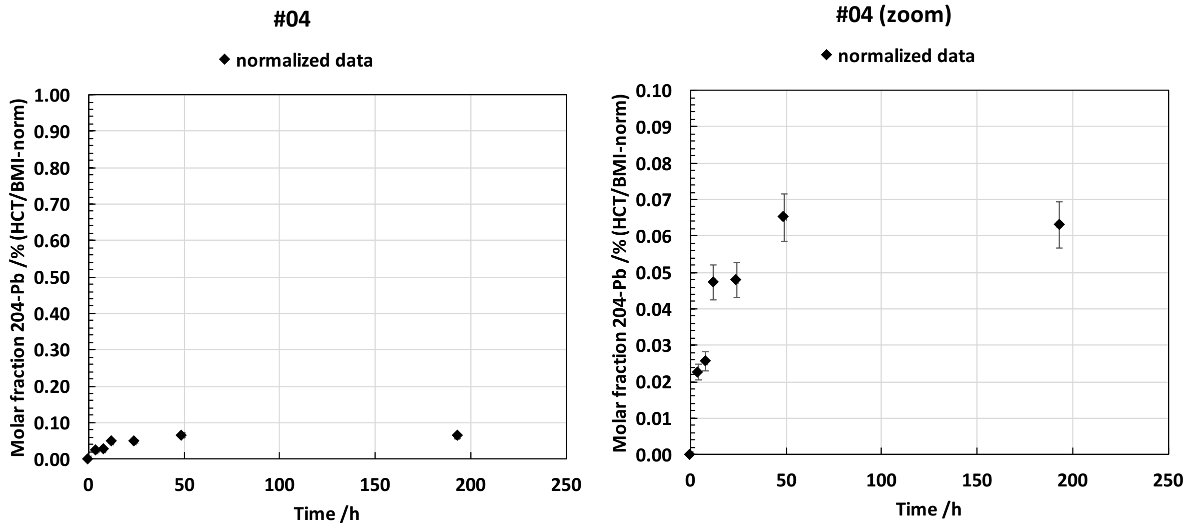

Figure S03 Subject-04: ^{204}Pb-molar fraction in Pb normalized to HCT/BMI.

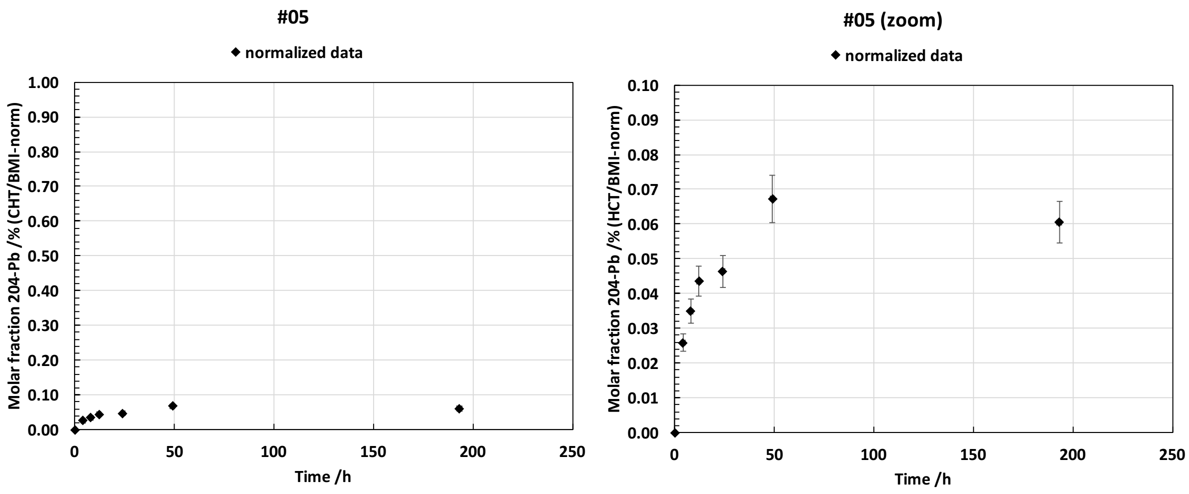

Figure S04 Subject-05: ^{204}Pb-molar fraction in Pb normalized to HCT/BMI.

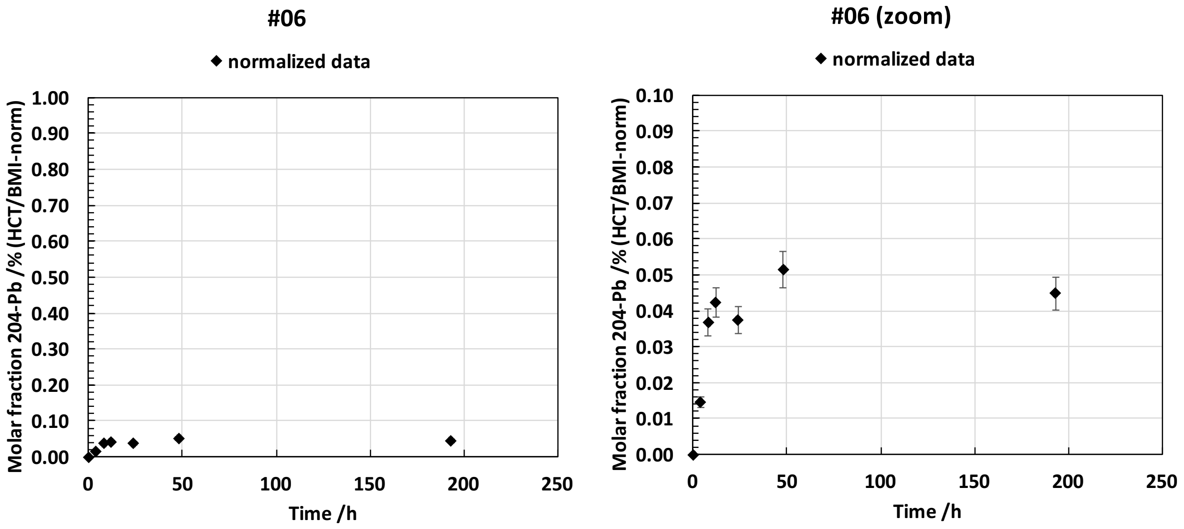

Figure S05 Subject-06: ^{204}Pb-molar fraction in Pb normalized to HCT/BMI.

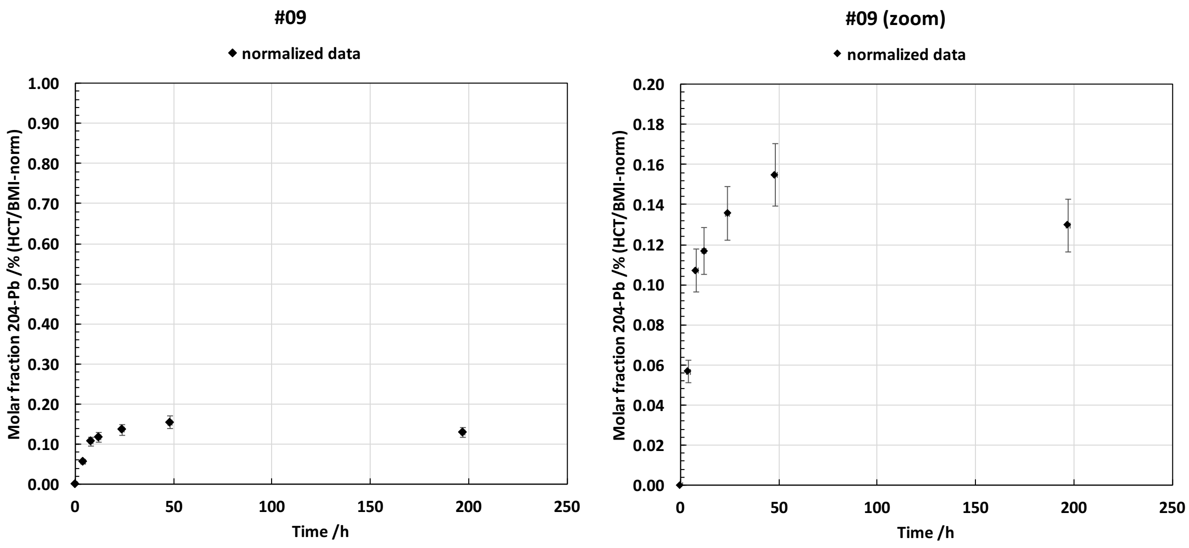

Figure S06 Subject-09: ^{204}Pb-molar fraction in Pb normalized to HCT/BMI.

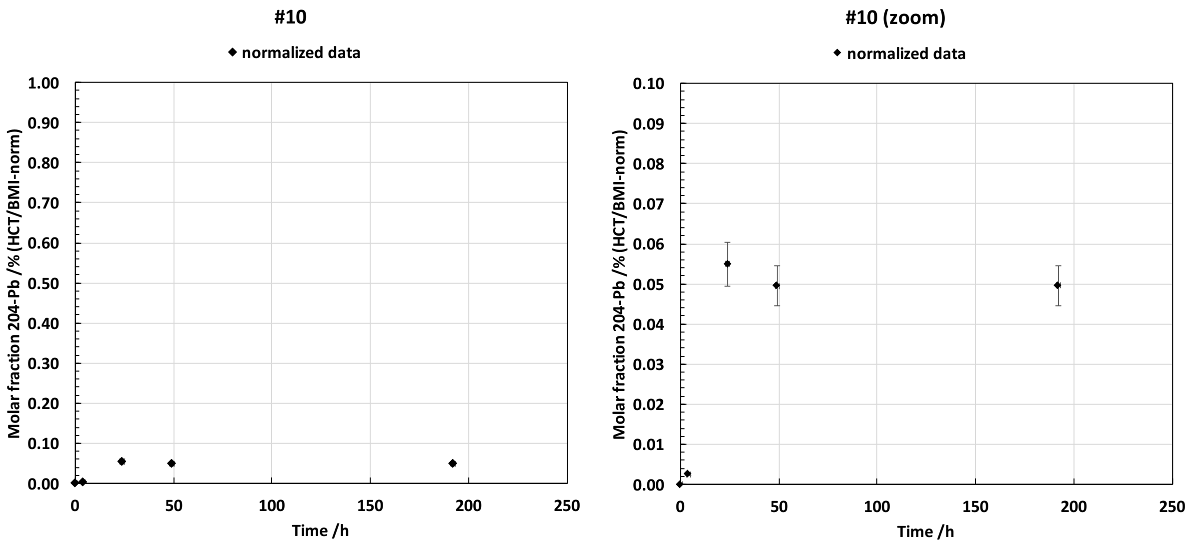

Figure S07 Subject-10: ^{204}Pb-molar fraction in Pb normalized to HCT/BMI.

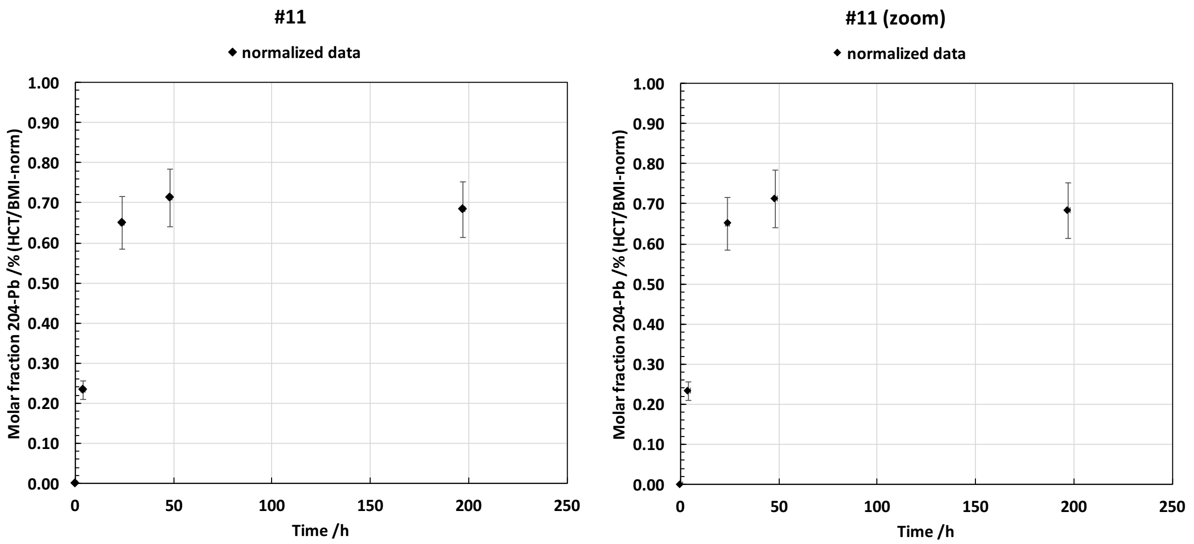

Figure S08 Subject-11: ^{204}Pb-molar fraction in Pb normalized to HCT/BMI.

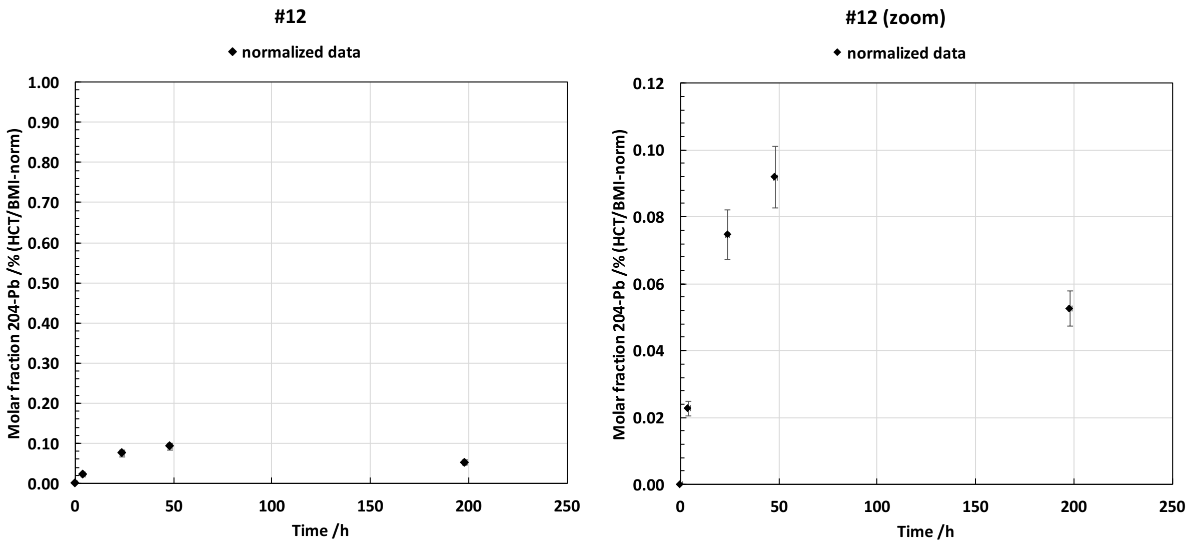

Figure S09 Subject-12: ^{204}Pb-molar fraction in Pb normalized to HCT/BMI.

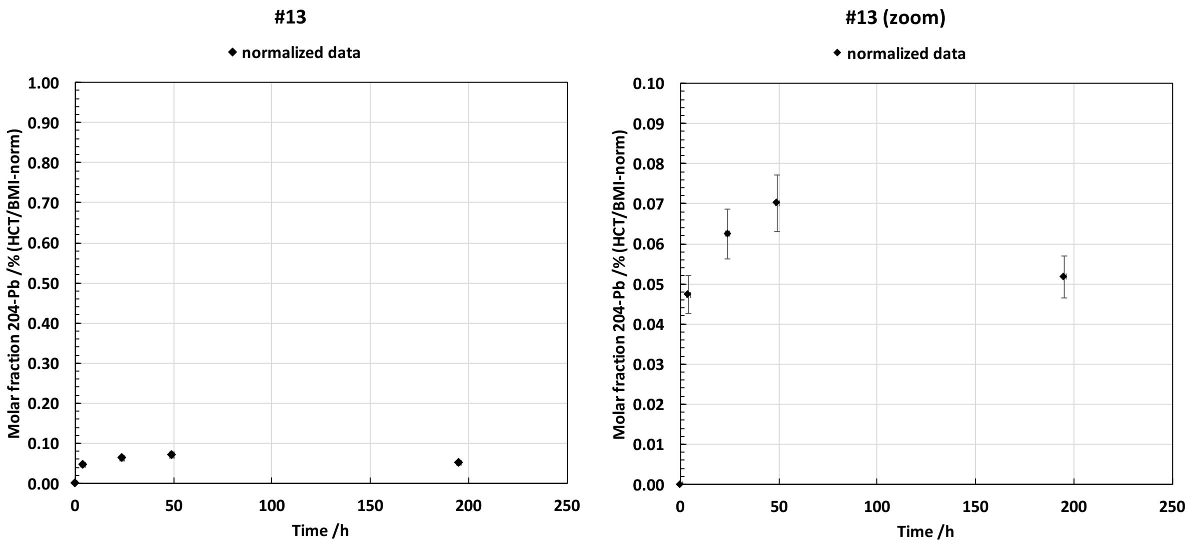

Figure S10 Subject-13: ^{204}Pb-molar fraction in Pb normalized to HCT/BMI.

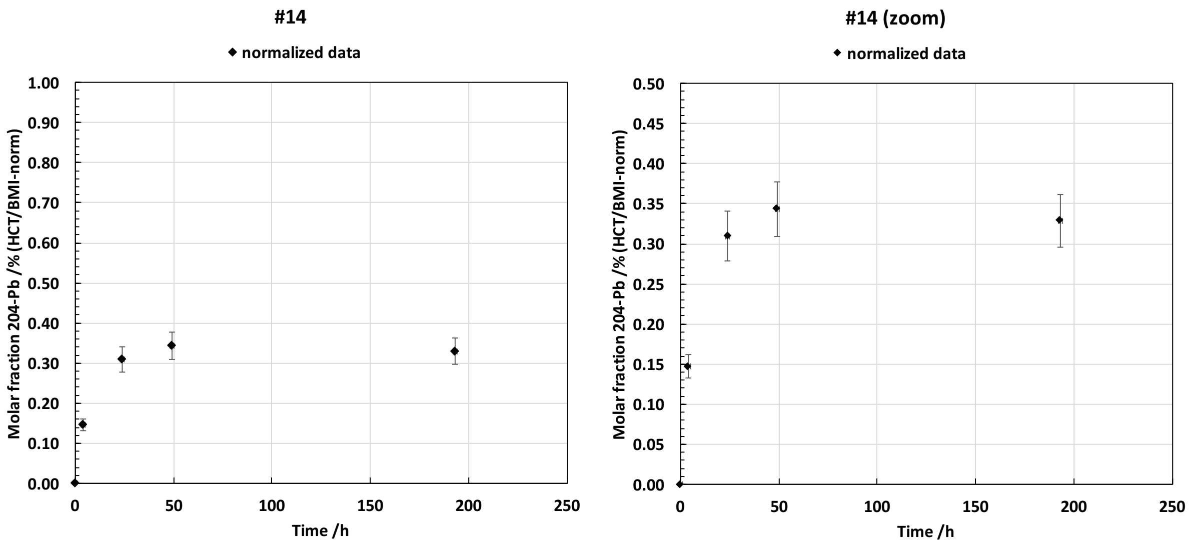

Figure S11 Subject-14: ^{204}Pb-molar fraction in Pb normalized to HCT/BMI.

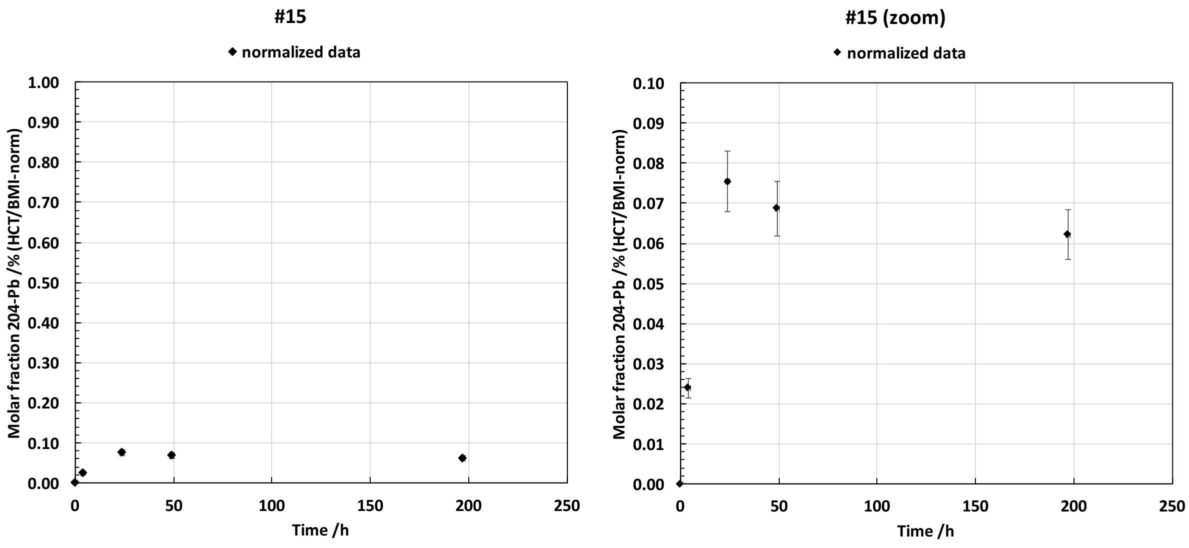

Figure S12 Subject-15: ^{204}Pb-molar fraction in Pb normalized to HCT/BMI.

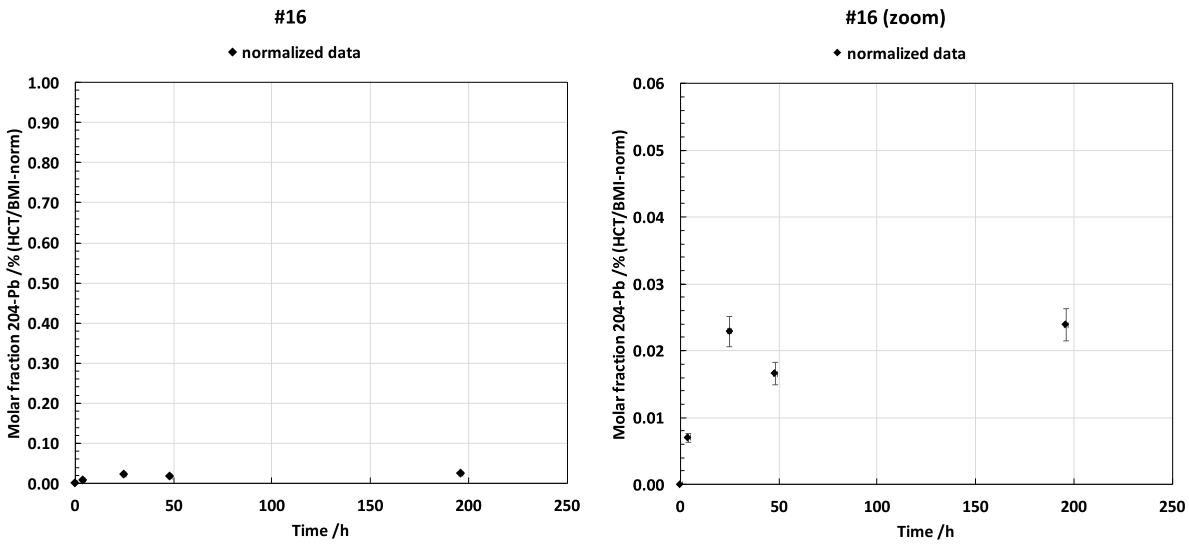

Figure S13 Subject-16: ^{204}Pb-molar fraction in Pb normalized to HCT/BMI.

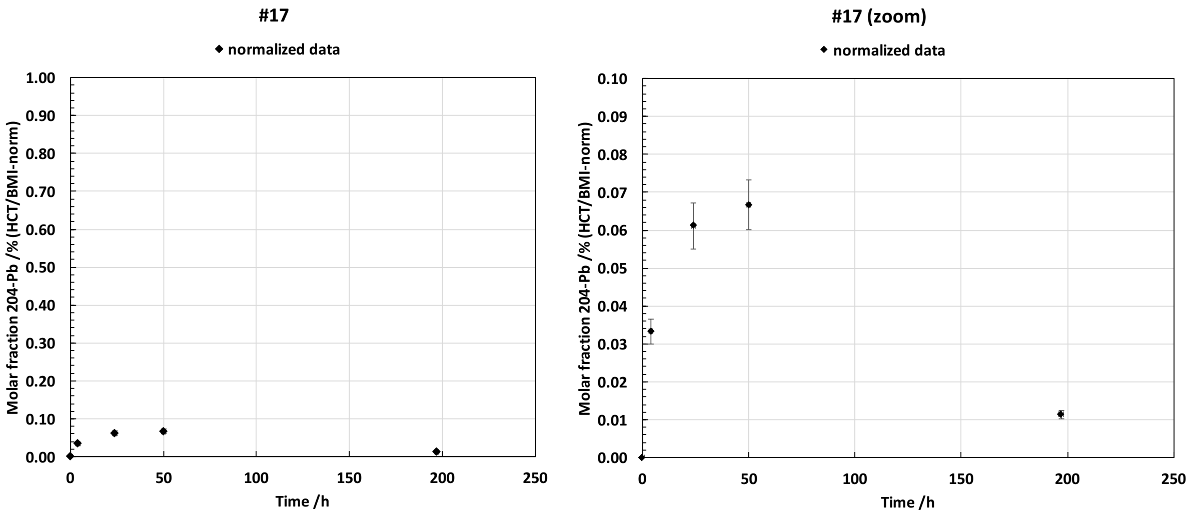

Figure S14 Subject-17: ^{204}Pb-molar fraction in Pb normalized to HCT/BMI. (Note: Sample *t*=192h shows a significant bias due to an increase in natural Pb at *t*=192h; see discussion below)

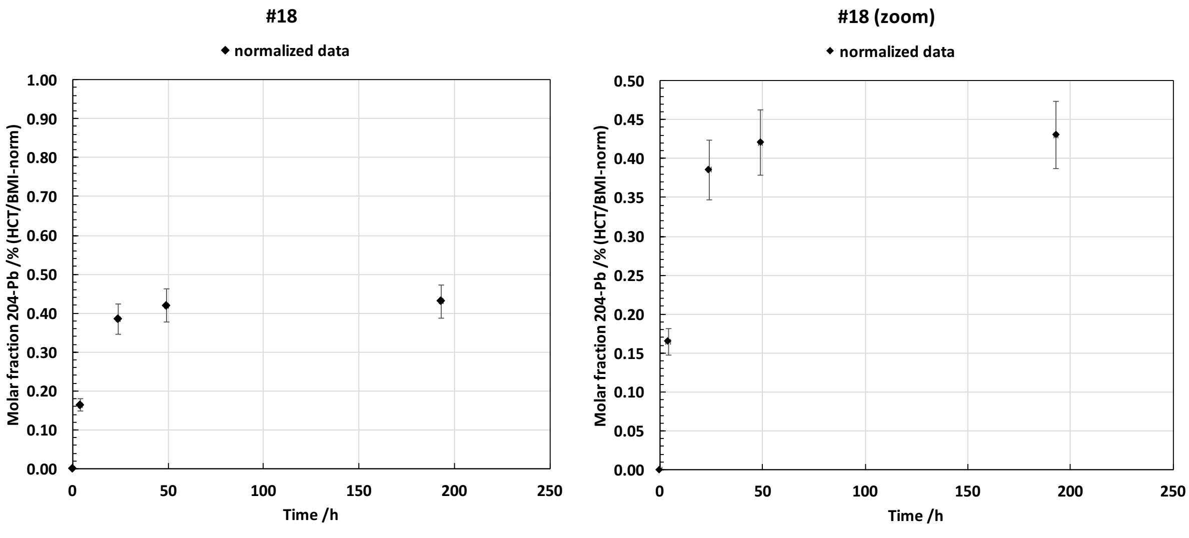

Figure S15 Subject-18: ^{204}Pb-molar fraction in Pb normalized to HCT/BMI.

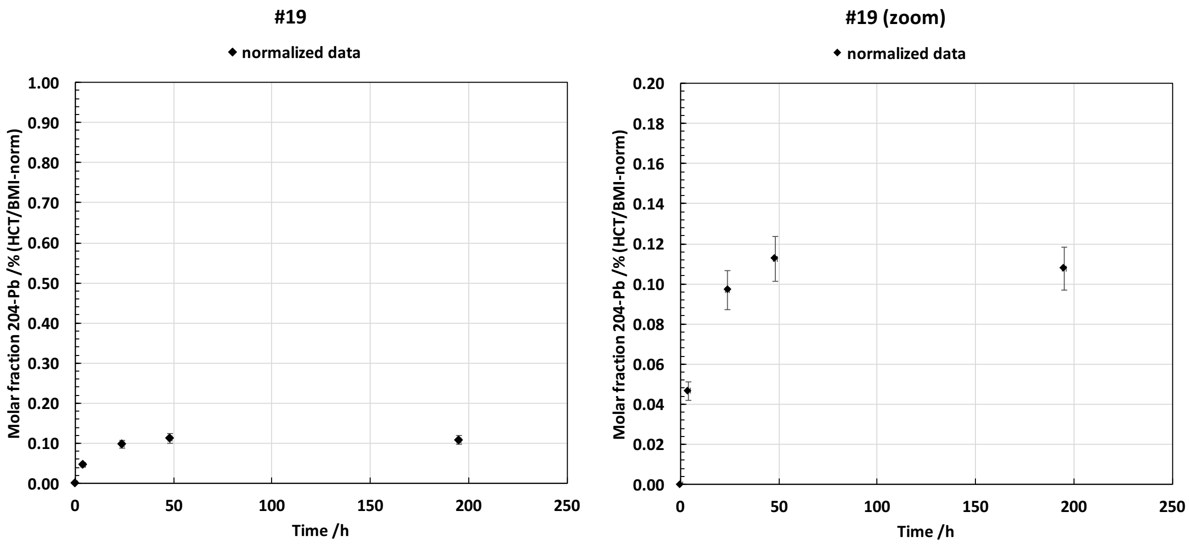

Figure S16 Subject-19: ^{204}Pb-molar fraction in Pb normalized to HCT/BMI.

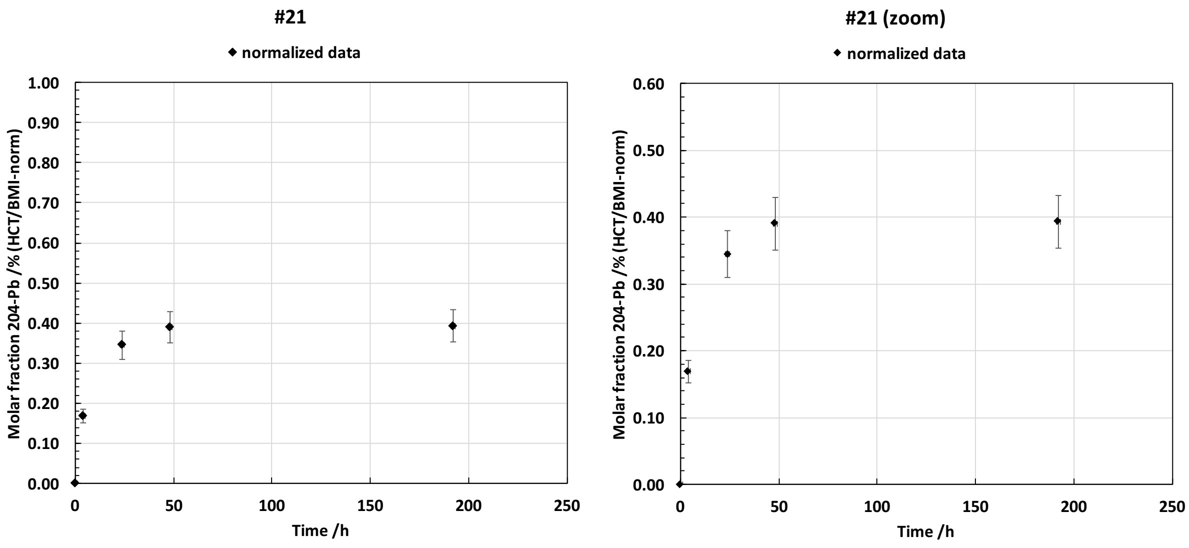

Figure S17 Subject-21: ^{204}Pb-molar fraction in Pb normalized to HCT/BMI.

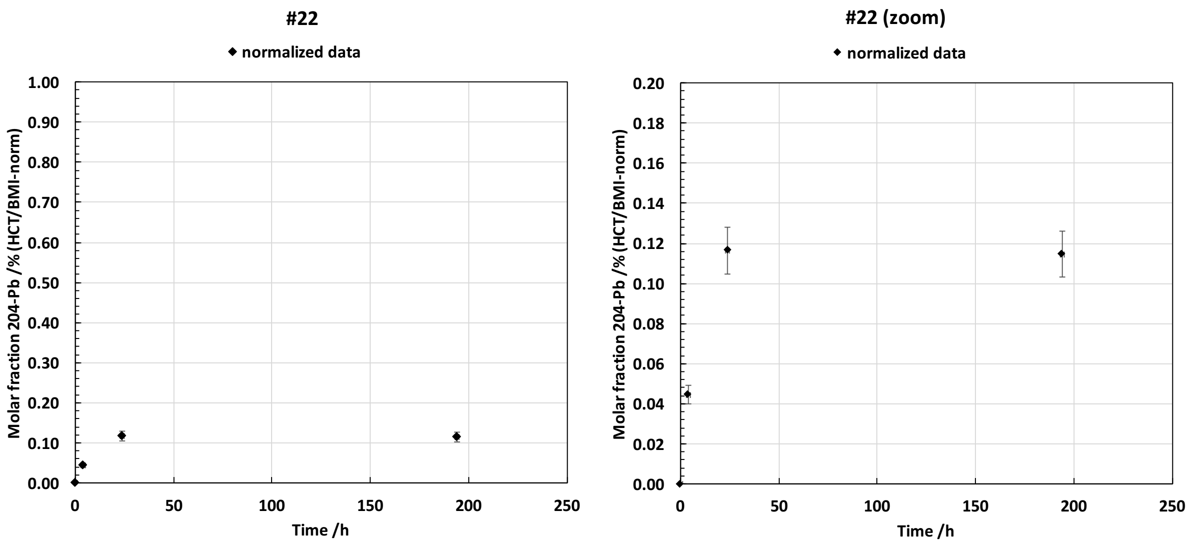

Figure S18 Subject-22: ^{204}Pb-molar fraction in Pb normalized to HCT/BMI. (Note: sample *t*=48h was not available)

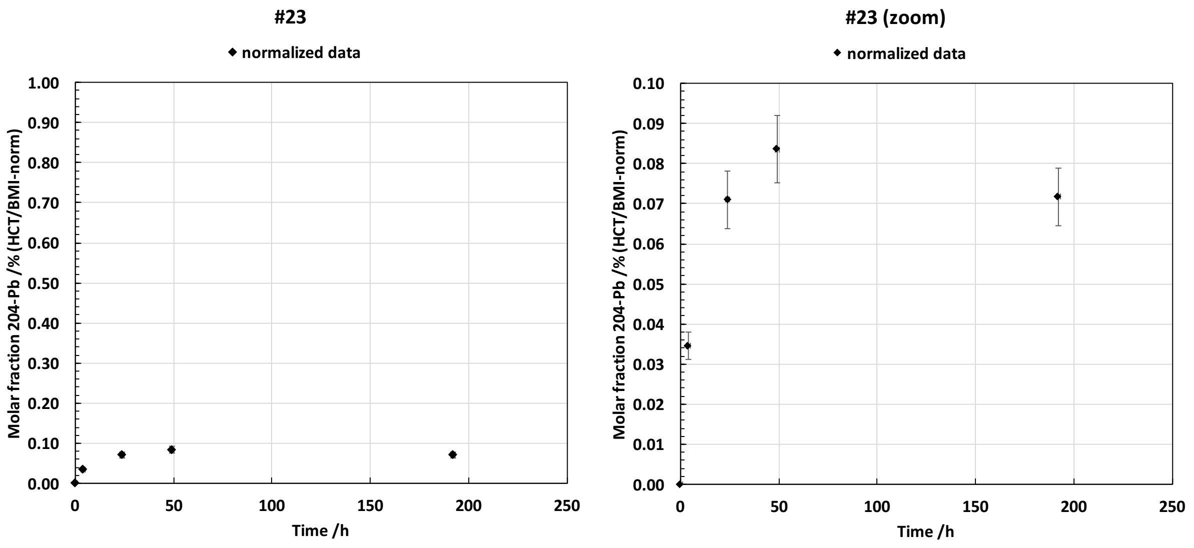

Figure S19 Subject-23: ^{204}Pb-molar fraction in Pb normalized to HCT/BMI.

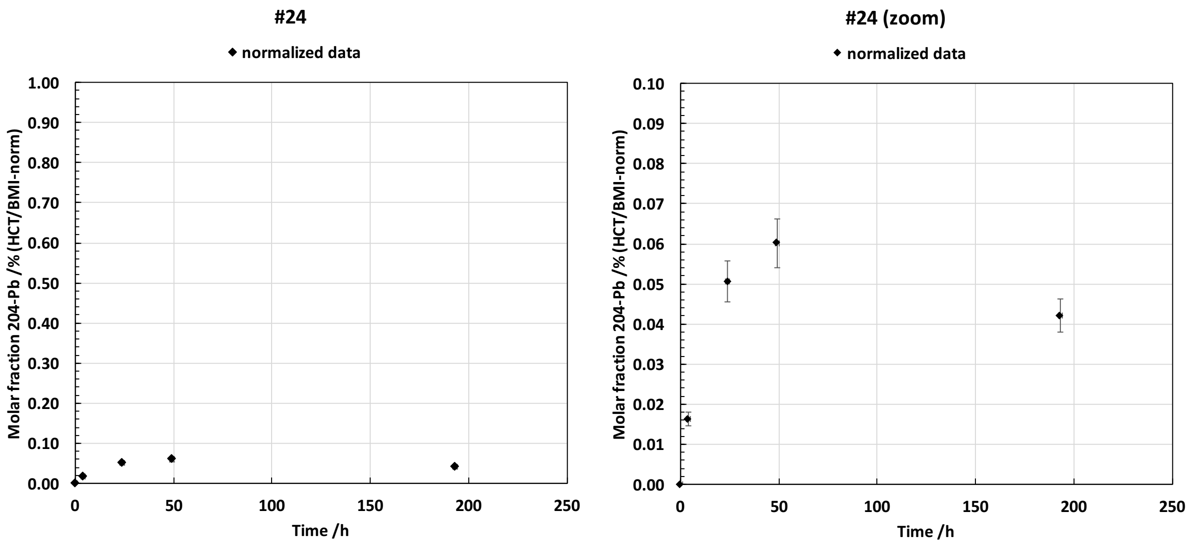

Figure S20 Subject-24: ^{204}Pb-molar fraction in Pb normalized to HCT/BMI.

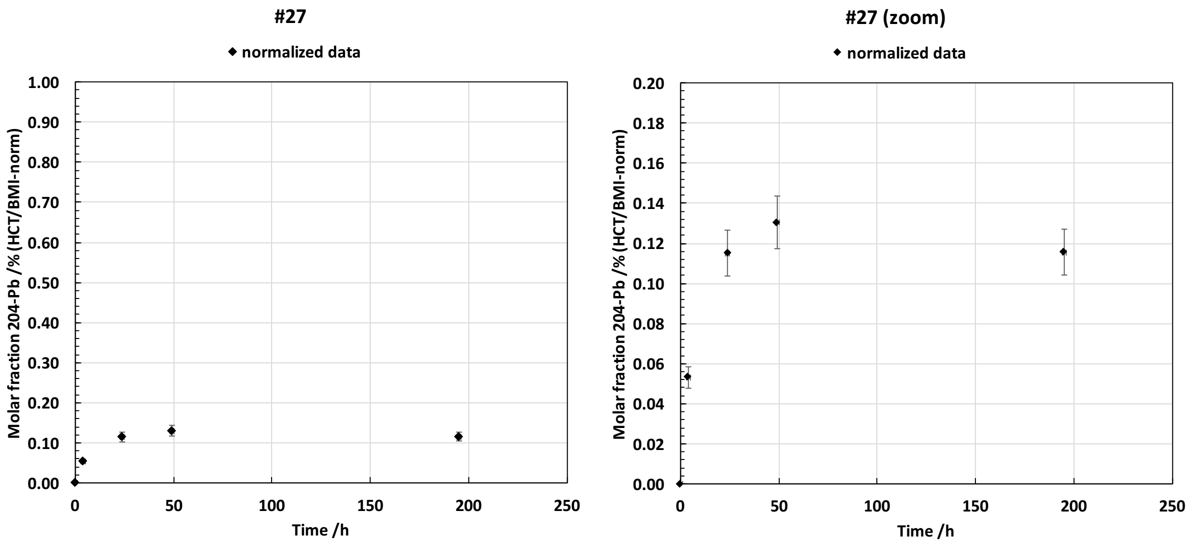

Figure S21 Subject-27: ^{204}Pb-molar fraction in Pb normalized to HCT/BMI.

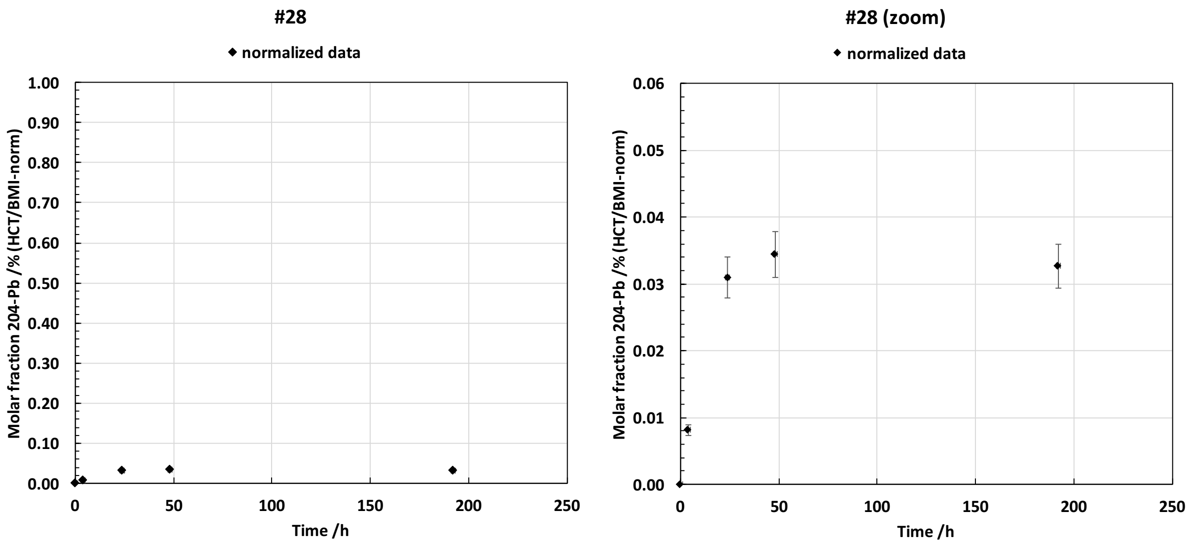

Figure S22 Subject-28: ^{204}Pb-molar fraction in Pb normalized to HCT/BMI.

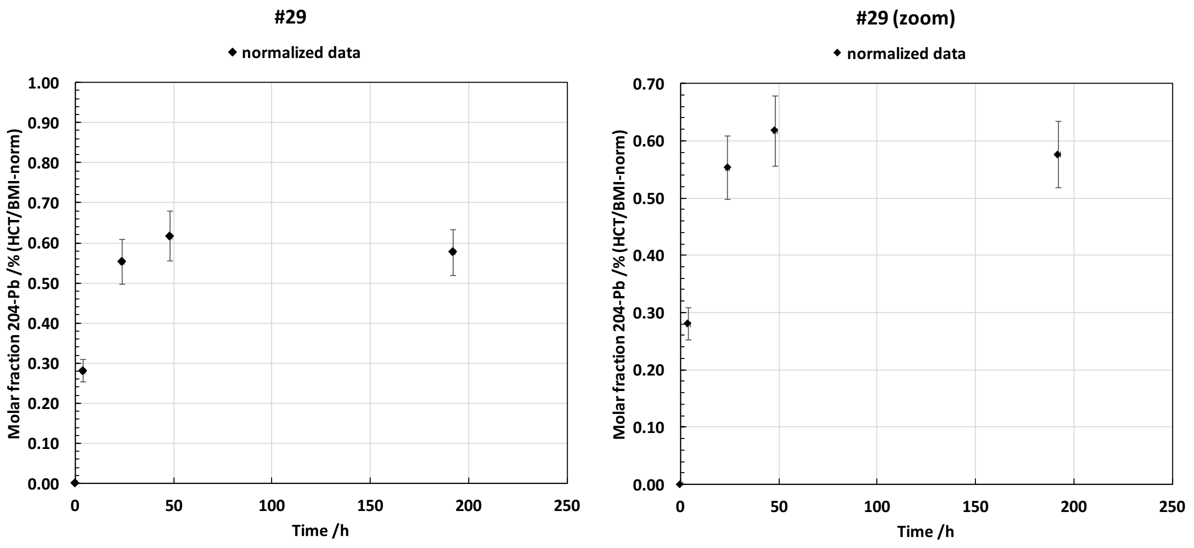

Figure S23 Subject-29: ^{204}Pb-molar fraction in Pb normalized to HCT/BMI.

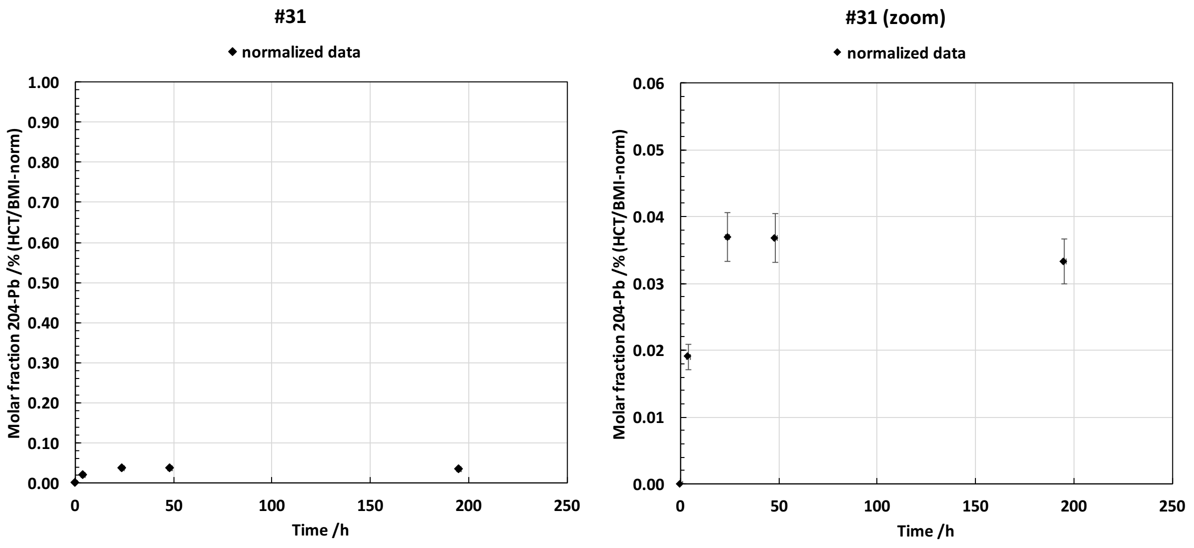

Figure S24 Subject-31: ^{204}Pb-molar fraction in Pb normalized to HCT/BMI.

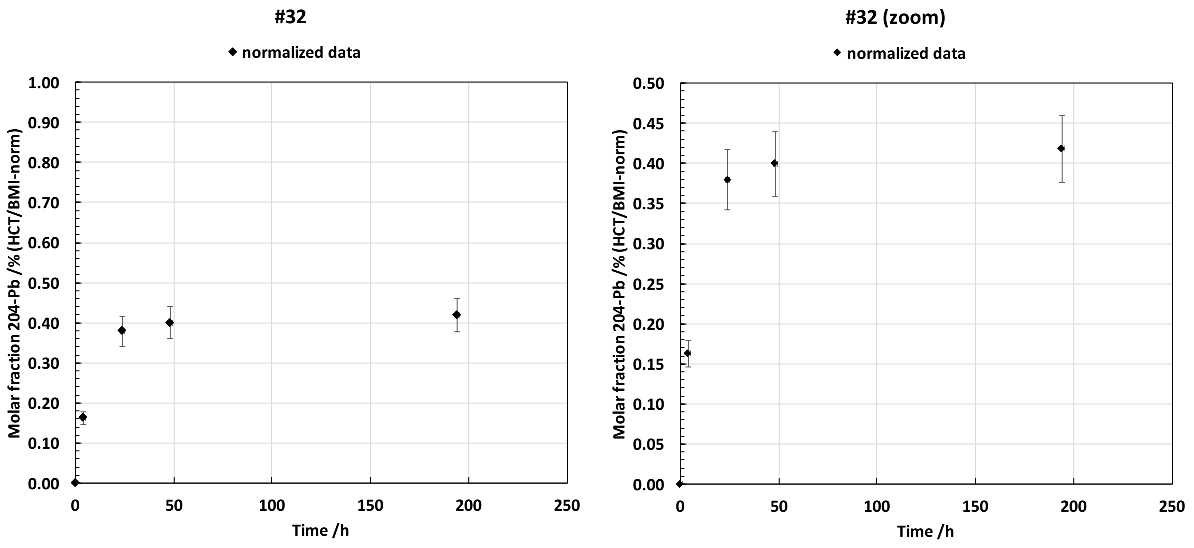

Figure S25 Subject-32: ^{204}Pb-molar fraction in Pb normalized to HCT/BMI.

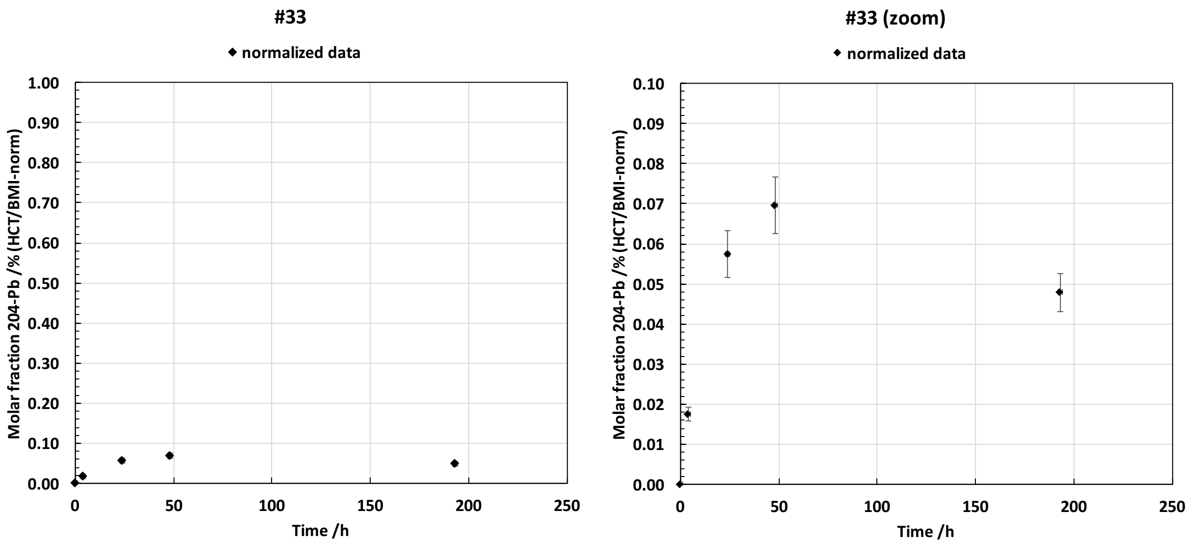

Figure S26 Subject-33: ^{204}Pb-molar fraction in Pb normalized to HCT/BMI.

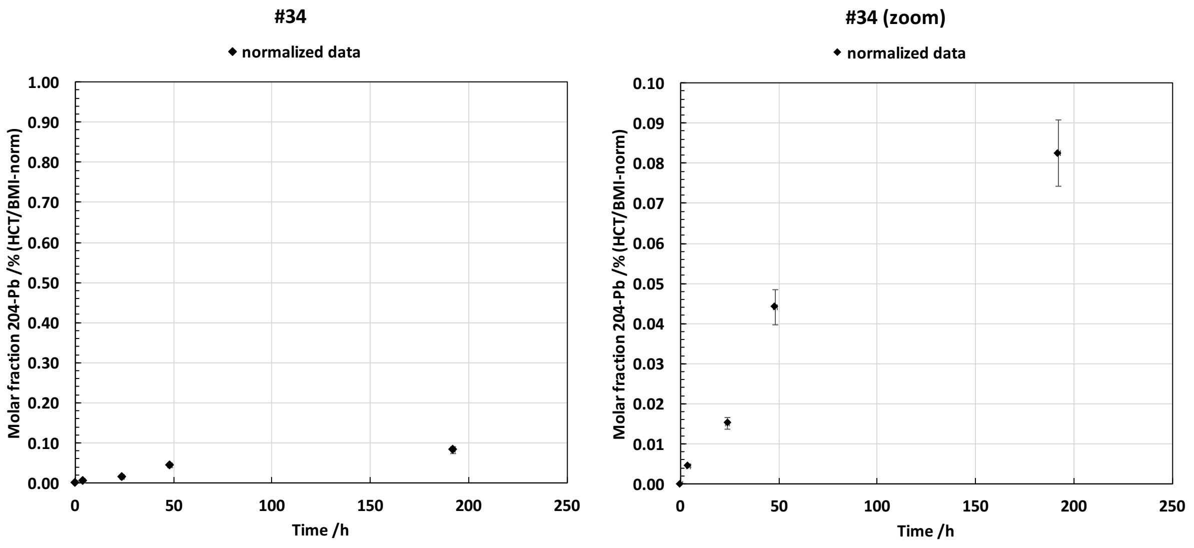

Figure S27 Subject-34: ^{204}Pb-molar fraction in Pb normalized to HCT/BMI.

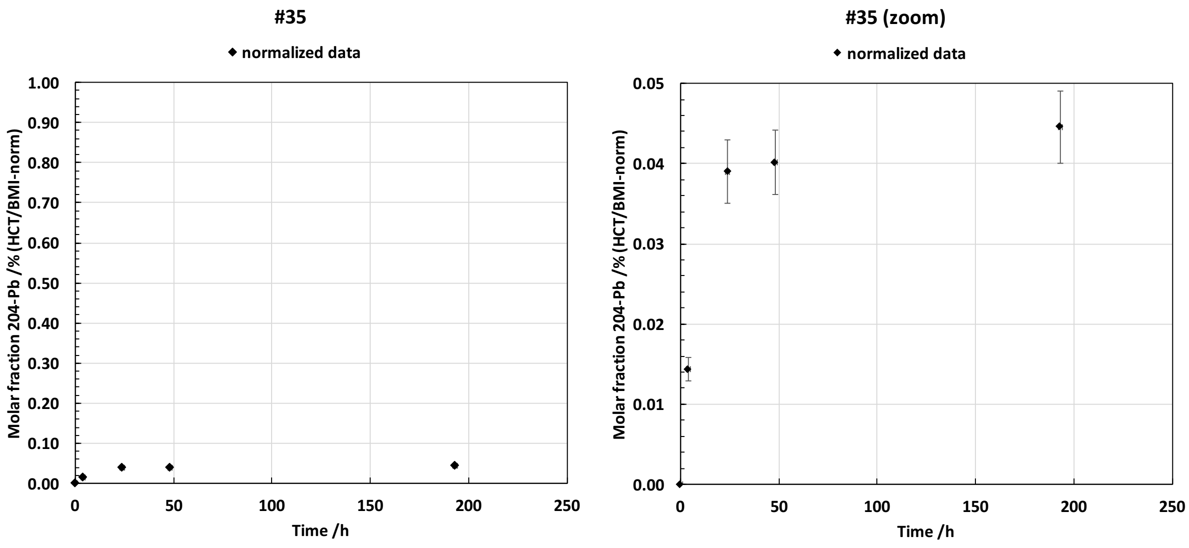

Figure S28 Subject-35: ^{204}Pb-molar fraction in Pb normalized to HCT/BMI.

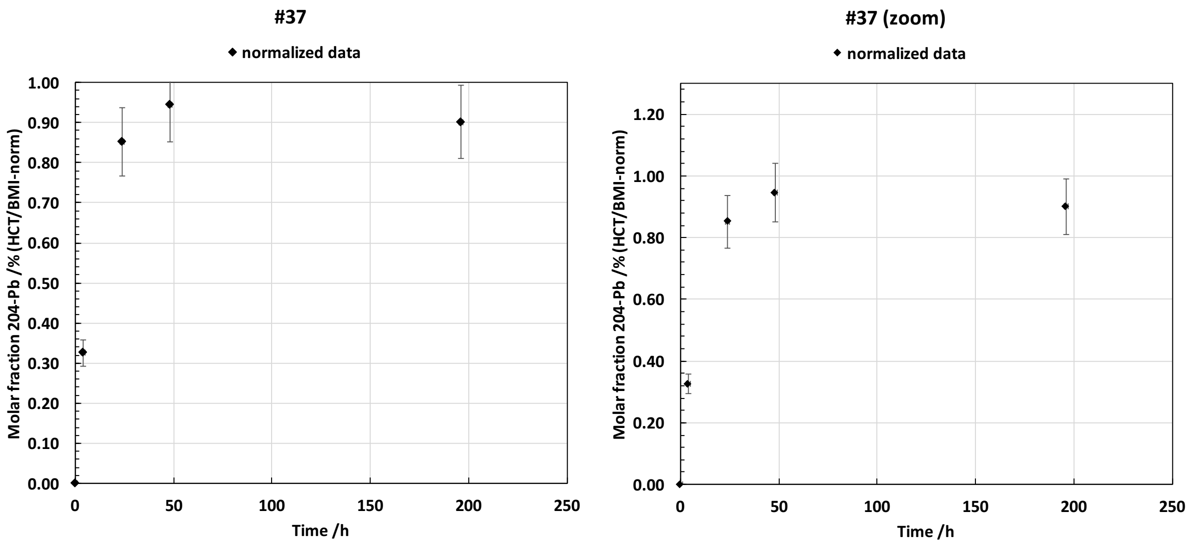

Figure S29 Subject-37: ^{204}Pb-molar fraction in Pb normalized to HCT/BMI.

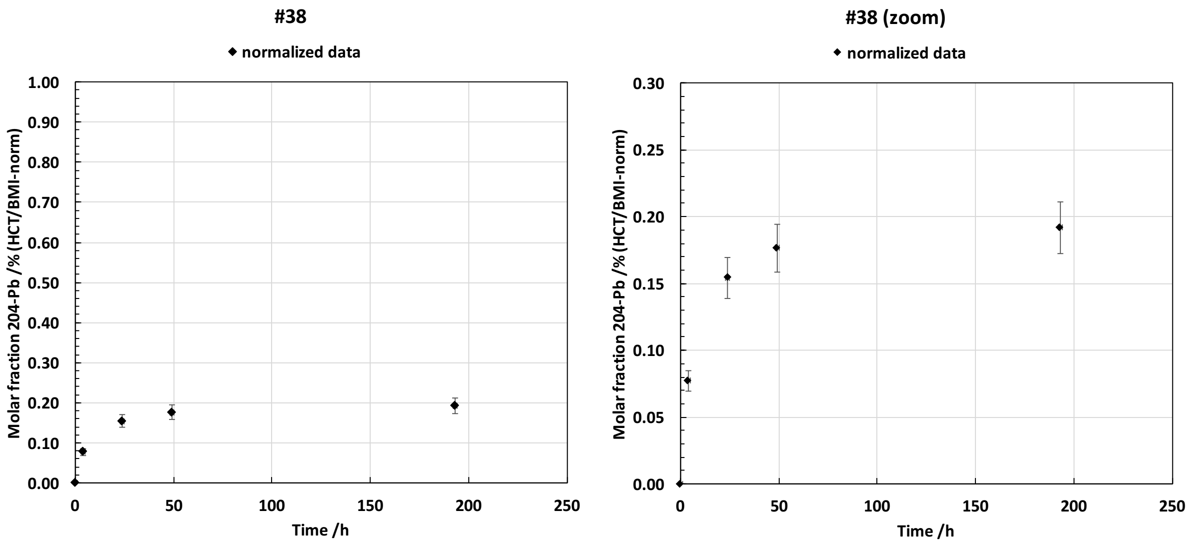

Figure S30 Subject-38: ^{204}Pb-molar fraction in Pb normalized to HCT/BMI.

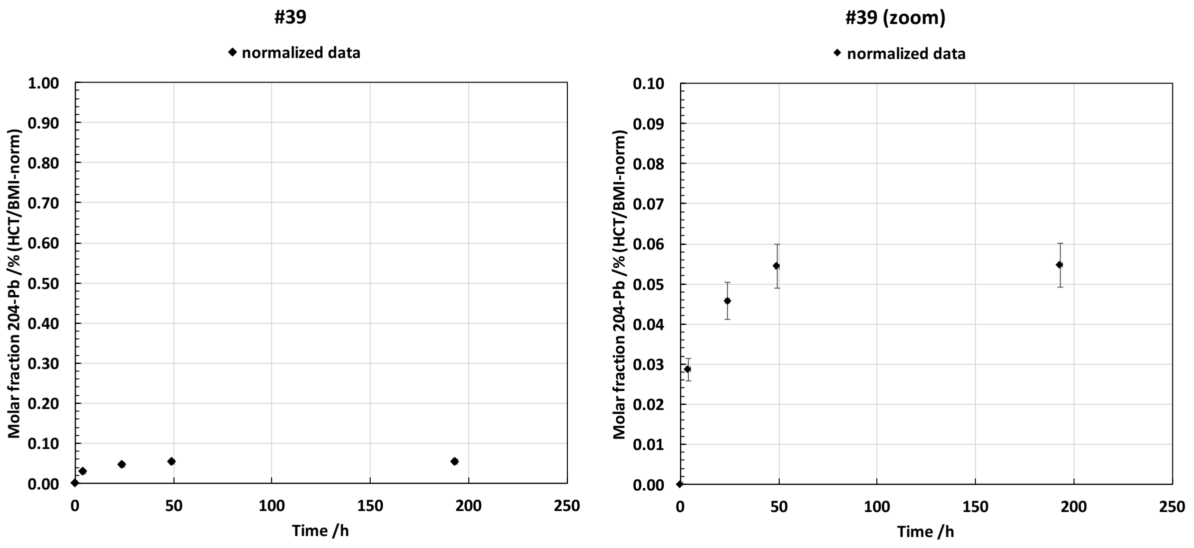

Figure S31 Subject-39: ^{204}Pb-molar fraction in Pb normalized to HCT/BMI.

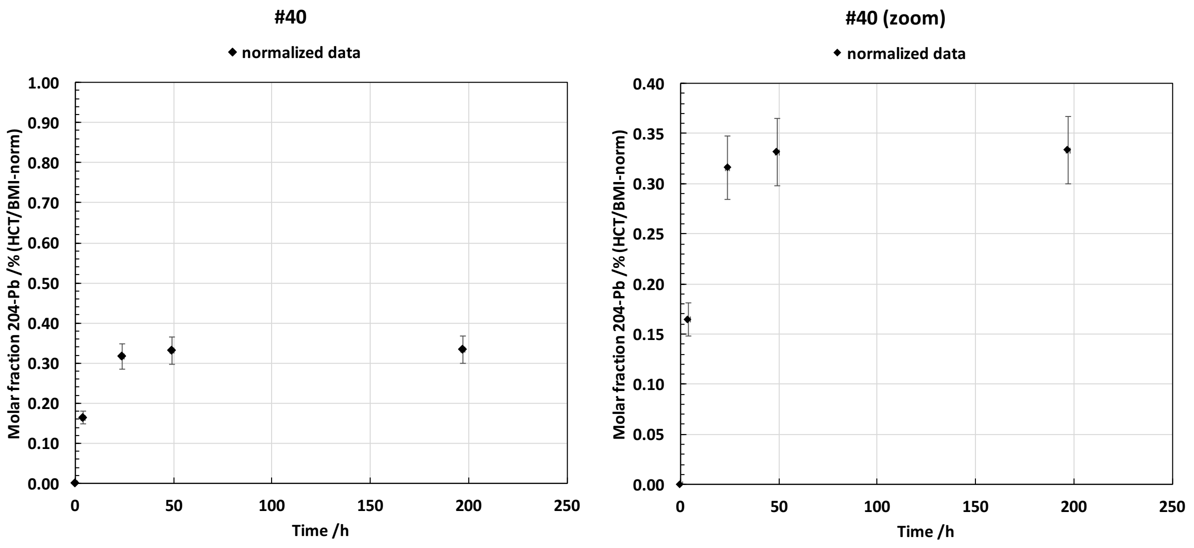

Figure S32 Subject-40: ^{204}Pb-molar fraction in Pb normalized to HCT/BMI.

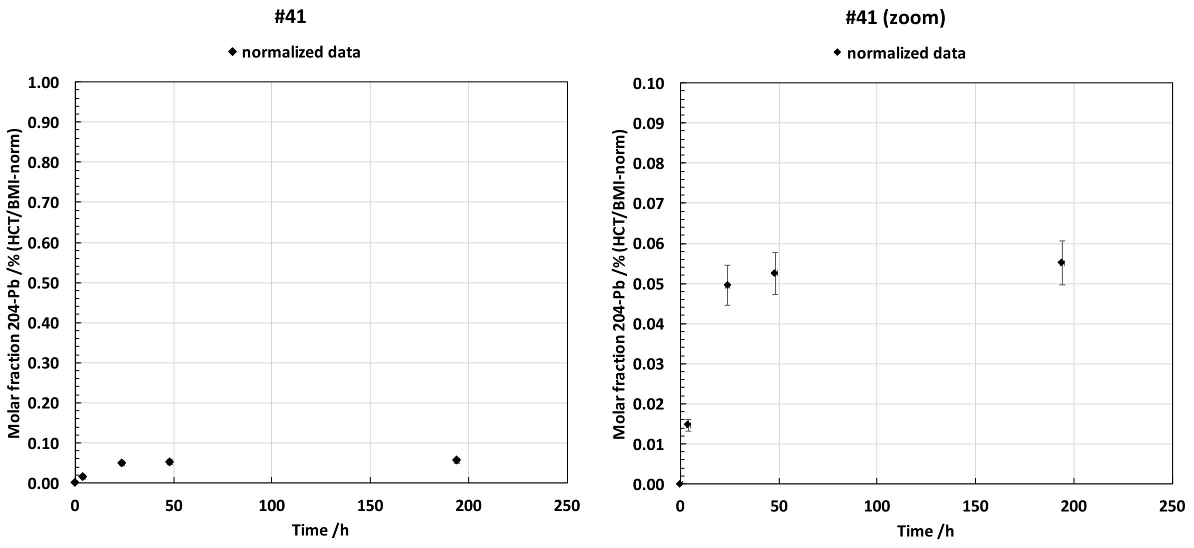

Figure S33 Subject-41: ^{204}Pb-molar fraction in Pb normalized to HCT/BMI.

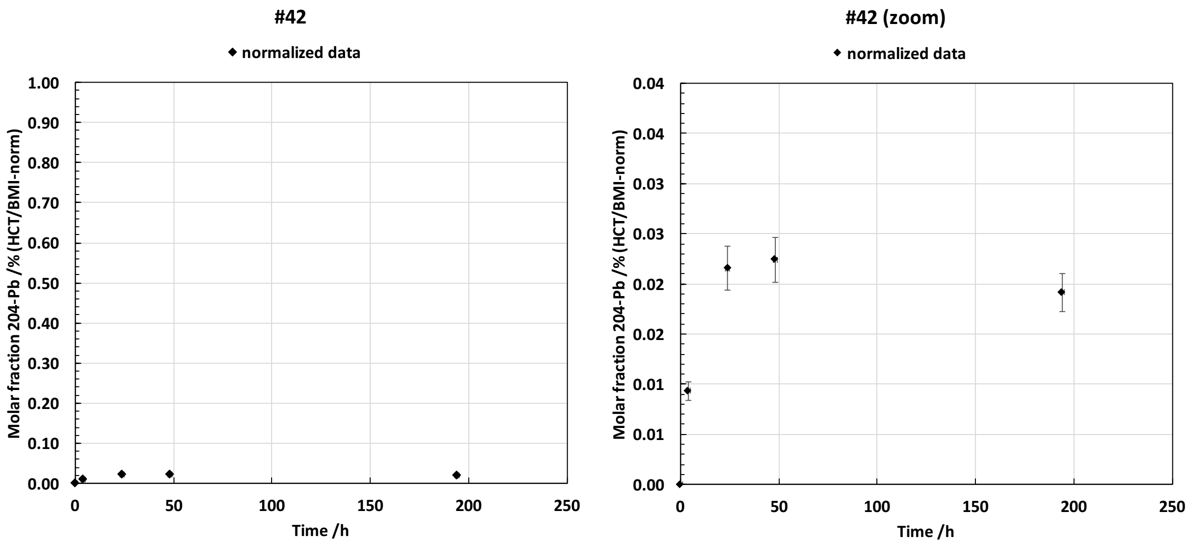

Figure S34 Subject-42: ^{204}Pb-molar fraction in Pb normalized to HCT/BMI.

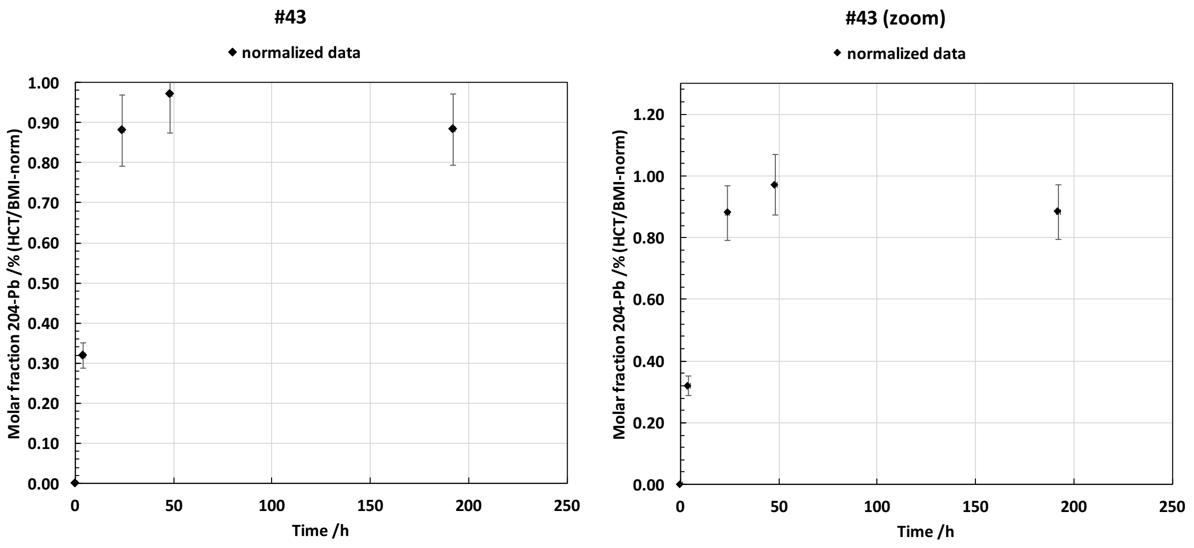

Figure S35 Subject-43: ^{204}Pb-molar fraction in Pb normalized to HCT/BMI.

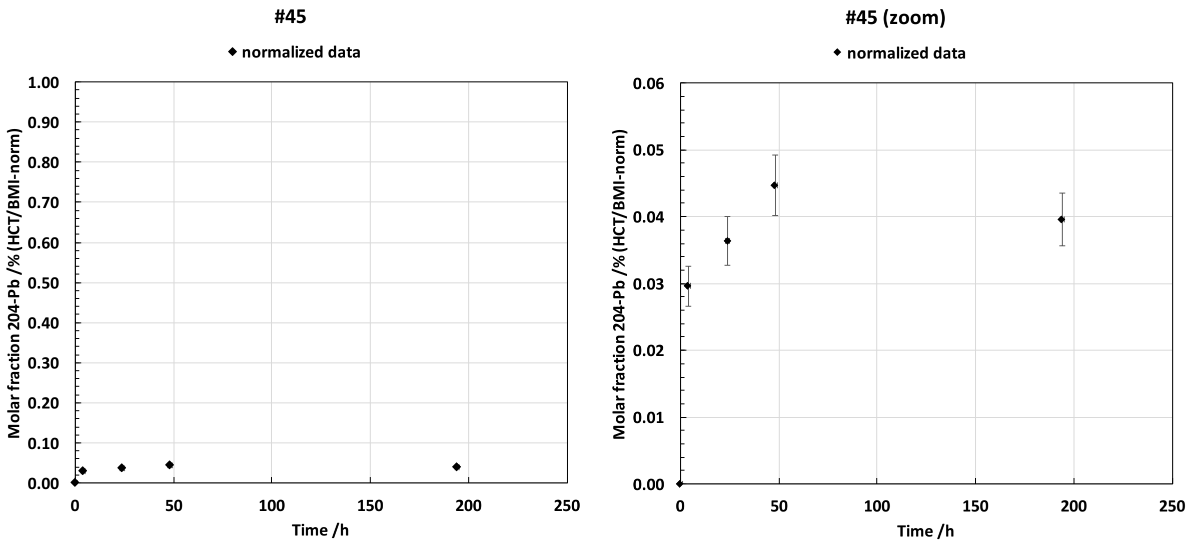

Figure S36 Subject-45: ^{204}Pb-molar fraction in Pb normalized to HCT/BMI.

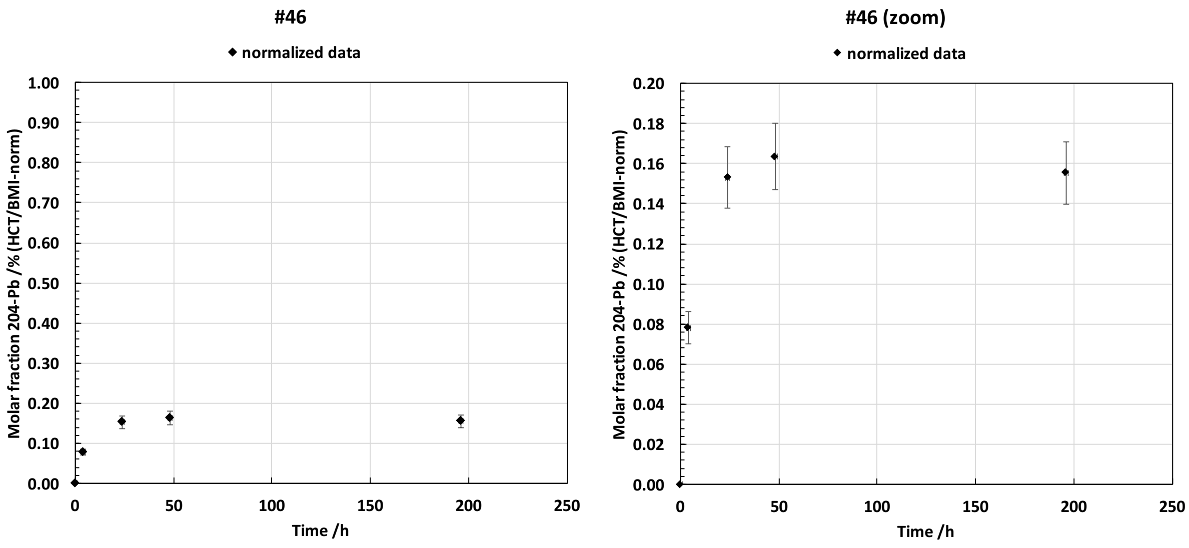

Figure S37 Subject-46: ^{204}Pb-molar fraction in Pb normalized to HCT/BMI.

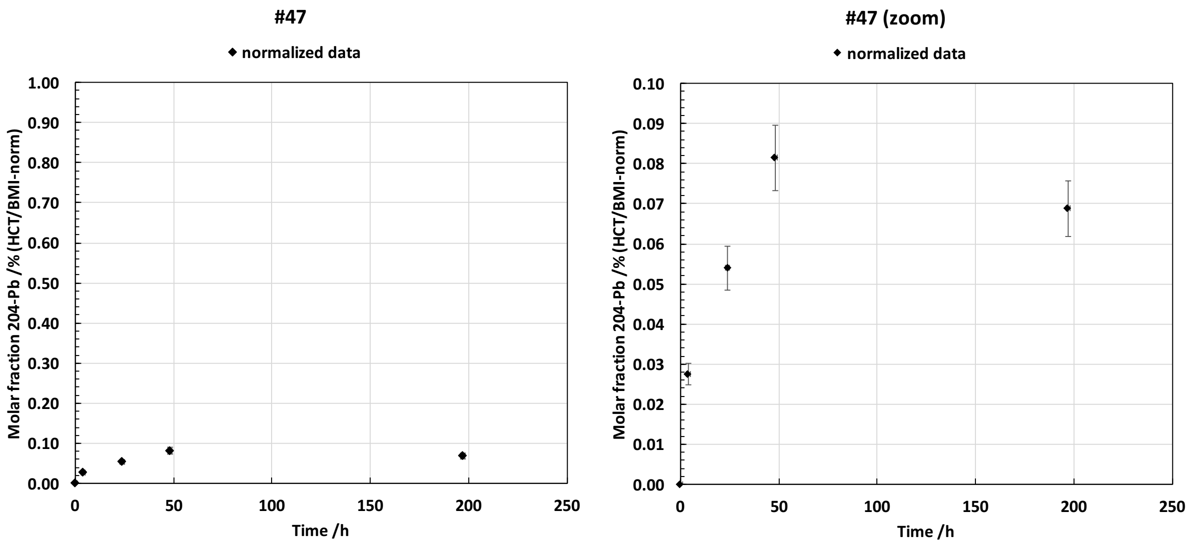

Figure S38 Subject-47: ^{204}Pb-molar fraction in Pb normalized to HCT/BMI.

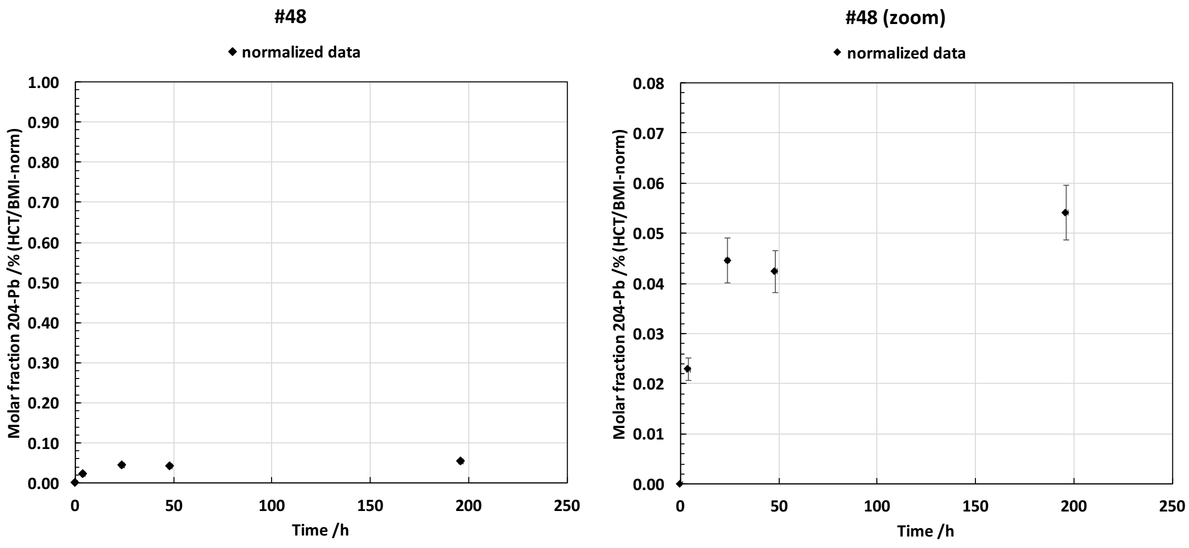

Figure S39 Subject-48: ^{204}Pb-molar fraction in Pb normalized to HCT/BMI.

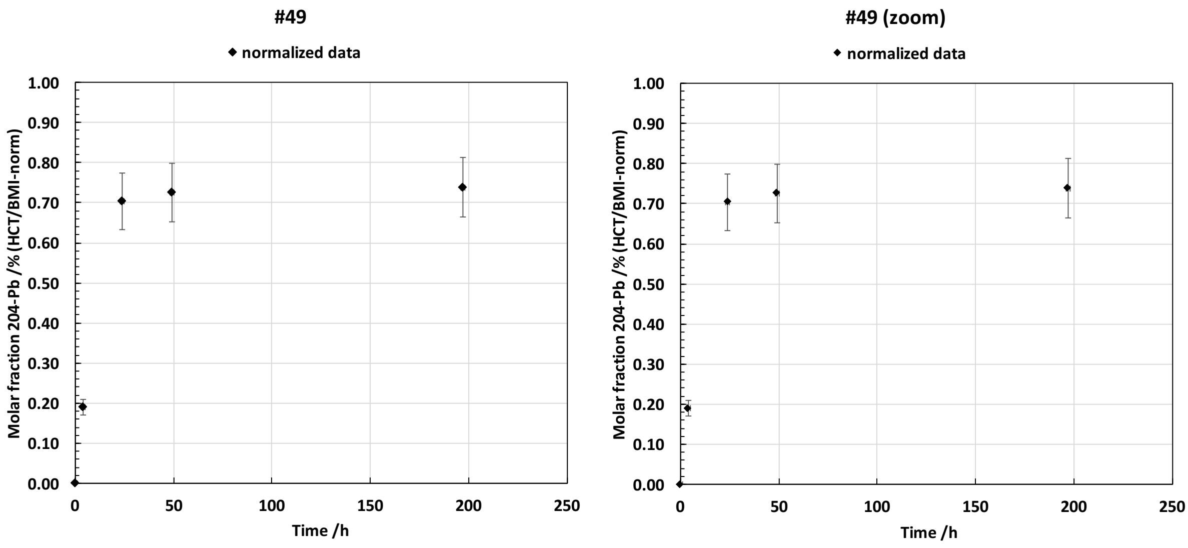

Figure S40 Subject-49: ^{204}Pb-molar fraction in Pb normalized to HCT/BMI.

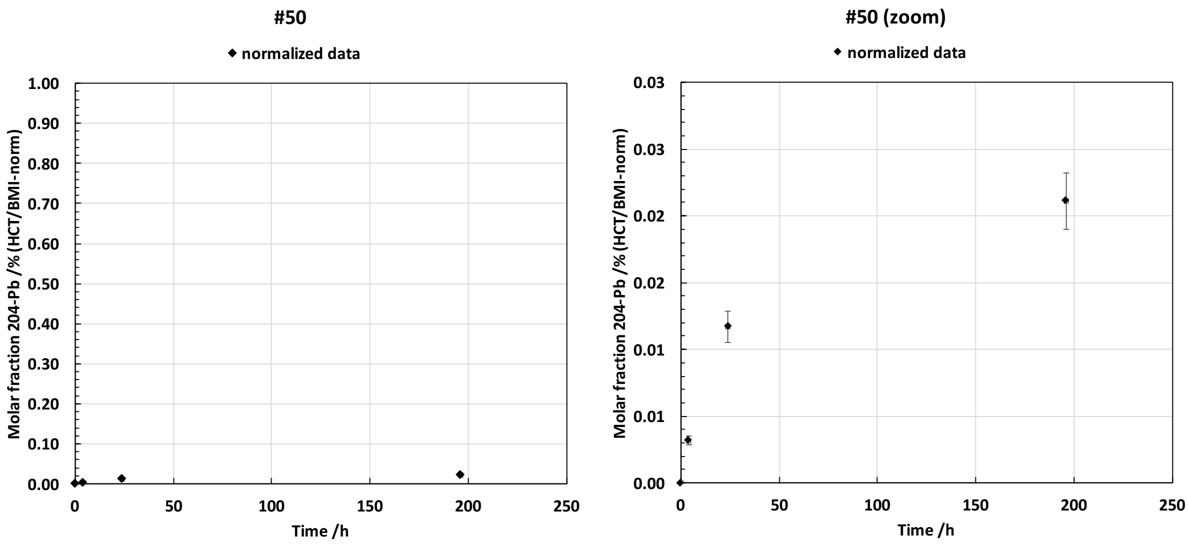

Figure S41 Subject-50: ^{204}Pb-molar fraction in Pb normalized to HCT/BMI. (Note: sample *t*=48h was not available)

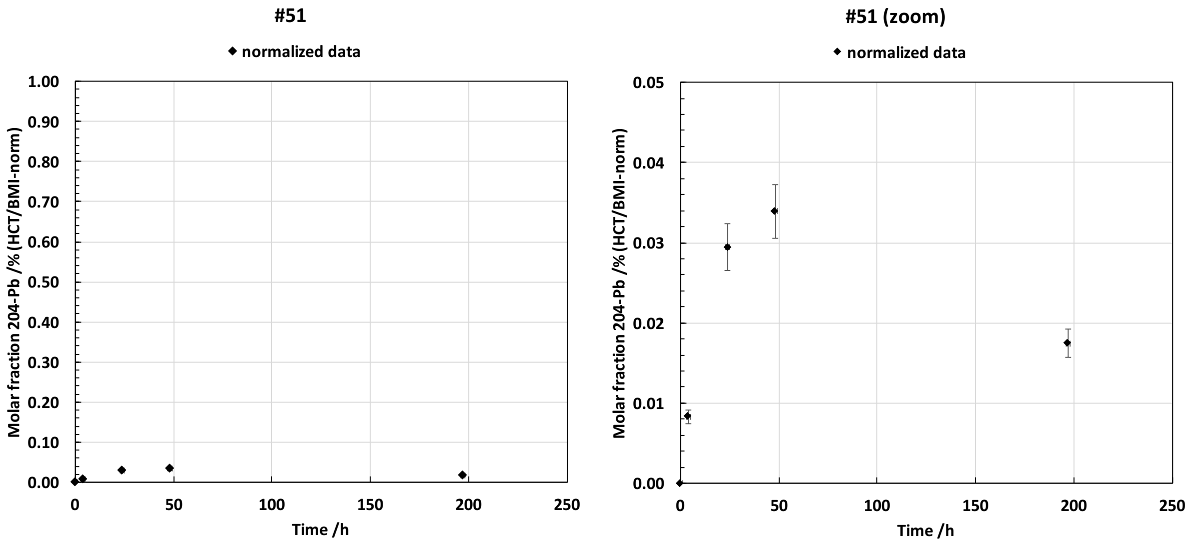

Figure S42 Subject-51: ^{204}Pb-molar fraction in Pb normalized to HCT/BMI

Table S04 Total Pb mass fraction and ^{204}Pb-tracer enrichment as well as mass fraction of creatinine in urine samples. Normalized Pb concentration in µg Pb/g creatinine (CREA) is given in bold letters. ($^{\#}$ secondary endpoint - column 11); *data evaluation by IPD v/r (value rejected) due to sample vessel contamination;

1	2	3	4	5	6	7	8	9	10	11$^{\#}$	12
**SAMPLE ID**	**SUBJECT**	**time**	**Total Pb in sample (µg/L)**	***U***	**Urine Creatinine (mg/dL) (CREA)**	**µg Pb / g CREA**	***U***	***x*(^{204}Pb) /%**	***U* (*x*(^{204}Pb)**	**µg ^{204}Pb-TRACER/ g CREA**	***U* (*x*(^{204}Pb)-norm**
G-LEAD_01_BL_U3_DIG	1	0	1.12	0.77	119.0	0.94	0.65				
G-LEAD_01_24h_U3_DIG	1	24	0.59	0.50	31.0	1.92	1.62	0.1600	0.1354	0.0031	0.0026
G-LEAD_02_BL_U3_DIG	2	0	0.97	0.70	137.7	0.70	0.51				
G-LEAD_02_24h_U3_DIG	2	24	0.34	0.30	32.6	1.03	0.92	1.1274	1.0049	0.0117	0.0104
G-LEAD_04_BL_U3_DIG	4	0	1.60	1.02	175.8	0.91	0.58				
G-LEAD_04_24h_U3_DIG*	4	24	0.64	0.52	44.6	1.43	1.17	v/r	v/r	v/r	v/r
G-LEAD_05_BL_U3_DIG	5	0	1.01	0.74	126.3	0.80	0.59				
G-LEAD_05_24h_U3_DIG	5	24	0.35	0.31	47.5	0.74	0.66	0.0974	0.0870	0.0007	0.0006
G-LEAD_06_BL_U3_DIG	6	0	1.91	1.12	241.1	0.79	0.46				
G-LEAD_06_24h_U3_DIG	6	24	1.07	0.73	80.4	1.33	0.90	0.1074	0.0730	0.0014	0.0010
G-LEAD_09_BL_U3_DIG	9	0	1.76	1.07	209.7	0.84	0.51				
G-LEAD_09_24h_U3_DIG	9	24	0.12	0.12	46.0	0.26	0.26	0.6758	0.6813	0.0018	0.0018
G-LEAD_10_BL_U1_DIG	10	0	0.61	0.37	127.0	0.48	0.29				
G-LEAD_10_24h_U1_DIG	10	24	0.57	0.34	48.3	1.17	0.70	0.0076	0.0046	0.0001	0.0001
G-LEAD_11_BL_U1_DIG	11	0	0.80	0.48	95.9	0.84	0.50				
G-LEAD_11_24h_U1_DIG	11	24	0.63	0.38	32.0	1.97	1.18	1.0362	0.6217	0.0205	0.0123
G-LEAD_12_BL_U1_DIG	12	0	0.61	0.36	137.1	0.44	0.27				
G-LEAD_12_24h_U1_DIG	12	24	0.39	0.23	61.3	0.64	0.38	0.0205	0.0123	0.0001	0.0001
G-LEAD_13_BL_U1_DIG	13	0	2.34	1.40	148.1	1.58	0.95				
G-LEAD_13_24h_U1_DIG	13	24	0.49	0.29	35.7	1.36	0.82	0.7118	0.4271	0.0097	0.0058
G-LEAD_14_BL_U1_DIG	14	0	1.10	0.66	157.9	0.70	0.42				
G-LEAD_14_24h_U1_DIG	14	24	1.02	0.61	42.5	2.40	1.44	0.6122	0.3673	0.0147	0.0088
G-LEAD_15_BL_U1_DIG	15	0	1.32	0.79	162.4	0.81	0.49				
G-LEAD_15_24h_U1_DIG	15	24	0.97	0.58	44.6	2.17	1.30	0.0053	0.0032	0.0001	0.0001
G-LEAD_16_BL_U1_DIG	16	0	1.57	0.94	167.0	0.94	0.57				

1	2	3	4	5	6	7	8	9	10	11#	12
SAMPLE ID	SUBJECT	time	Total Pb in sample (µg/L)	*U*	Urine Creatinine (mg/dL) (CREA)	µg Pb / g CREA	*U*	$x(^{204}Pb)$ /%	U $(x(^{204}Pb)$	µg ^{204}Pb-TRACER/ g CREA	U $(x(^{204}Pb)$-norm
G-LEAD_16_24h_U1_DIG	16	24	0.76	0.46	29.3	2.61	1.56	0.1783	0.1070	0.0047	0.0028
G-LEAD_17_BL_U1_DIG	17	0	0.85	0.51	169.0	0.50	0.30				
G-LEAD_17_24h_U1_DIG	17	24	0.62	0.37	80.5	0.77	0.46	0.5143	0.3086	0.0040	0.0024
G-LEAD_18_BL_U1_DIG	18	0	0.99	0.60	74.7	1.33	0.80				
G-LEAD_18_24h_U1_DIG	18	24	0.70	0.42	52.4	1.34	0.80	1.0693	0.6416	0.0143	0.0086
G-LEAD_19_BL_U1_DIG	19	0	0.73	0.44	143.0	0.51	0.31				
G-LEAD_19_24h_U1_DIG	19	24	1.66	1.00	86.0	1.93	1.16	0.2000	0.2000	0.0039	0.0039
G-LEAD_21_BL_U1_DIG	21	0	1.50	0.90	185.8	0.81	0.48				
G-LEAD_21_24h_U1_DIG	21	24	0.66	0.40	71.0	0.94	0.56	1.6823	1.0094	0.0157	0.0094
G-LEAD_22_BL_U1_DIG	22	0	0.64	0.39	89.8	0.72	0.43				
G-LEAD_22_24h_U1_DIG	22	24	0.59	0.35	33.4	1.77	1.06	0.0849	0.0509	0.0015	0.0009
G-LEAD_23_BL_U1_DIG	23	0	0.75	0.45	233.1	0.32	0.19				
G-LEAD_23_24h_U1_DIG	23	24	0.76	0.46	48.5	1.56	0.94	0.1905	0.1143	0.0030	0.0018
G-LEAD_24_BL_U1_DIG	24	0	0.72	0.43	162.3	0.44	0.27				
G-LEAD_24_24h_U1_DIG	24	24	0.57	0.34	46.1	1.24	0.75	0.2385	0.1431	0.0030	0.0018
G-LEAD_27_BL_U1_DIG	27	0	2.52	1.51	157.4	1.60	0.96				
G-LEAD_27_24h_U1_DIG	27	24	2.01	1.20	62.5	3.21	1.93	0.0901	0.0540	0.0029	0.0017
G-LEAD_28_BL_U1_DIG	28	0	1.03	0.62	209.6	0.49	0.29				
G-LEAD_28_24h_U1_DIG	28	24	0.72	0.43	48.6	1.47	0.88	0.2349	0.1409	0.0035	0.0021
G-LEAD_29_BL_U1_DIG	29	0	0.62	0.37	68.6	0.91	0.54				
G-LEAD_29_24h_U1_DIG	29	24	1.07	0.64	33.1	3.22	1.93	0.4633	0.2780	0.0149	0.0090
G-LEAD_31_BL_U1_DIG	31	0	1.00	0.60	181.9	0.55	0.33				
G-LEAD_31_24h_U1_DIG	31	24	0.71	0.43	60.2	1.18	0.71	0.0278	0.0167	0.0003	0.0002
G-LEAD_32_BL_U1_DIG	32	0	0.43	0.26	87.3	0.49	0.30				
G-LEAD_32_24h_U1_DIG	32	24	0.81	0.49	48.9	1.66	1.00	0.3528	0.2117	0.0059	0.0035
G-LEAD_33_BL_U1_DIG	33	0	0.89	0.53	190.4	0.47	0.28				
G-LEAD_33_24h_U1_DIG	33	24	0.59	0.35	31.9	1.85	1.11	0.2448	0.1469	0.0045	0.0027
G-LEAD_34_BL_U1_DIG	34	0	0.52	0.31	144.6	0.36	0.22				

1	2	3	4	5	6	7	8	9	10	11#	12
**SAMPLE ID**	**SUBJECT**	**time**	**Total Pb in sample (µg/L)**	***U***	**Urine Creatinine (mg/dL) (CREA)**	**µg Pb / g CREA**	***U***	**$x(^{204}Pb)$ /%**	***U*** **$(x(^{204}Pb)$**	**µg ^{204}Pb-TRACER/ g CREA**	***U*** **$(x(^{204}Pb)$-norm**
G-LEAD_34_24h_U1_DIG	34	24	0.46	0.28	45.3	1.01	0.61	0.5000	0.5000	0.0051	0.0030
G-LEAD_35_BL_U1_DIG	35	0	0.88	0.53	264.1	0.33	0.20				
G-LEAD_35_24h_U1_DIG	35	24	0.04	0.03	52.4	0.09	0.05	0.2000	0.2000	0.0002	0.0002
G-LEAD_37_BL_U1_DIG	37	0	1.29	0.77	239.2	0.54	0.32				
G-LEAD_37_24h_U1_DIG	37	24	0.66	0.39	94.8	0.69	0.42	0.4700	0.4700	0.0033	0.0033
G-LEAD_38_BL_U1_DIG	38	0	0.77	0.46	164.3	0.47	0.28				
G-LEAD_38_24h_U1_DIG	38	24	1.10	0.66	46.2	2.39	1.43	0.6000	0.6000	0.0143	0.0143
G_LEAD_39_BL_U1_DIG	39	0	1.57	0.94	361.6	0.44	0.26				
G-LEAD_39_24h_U1_DIG	39	24	0.62	0.37	97.3	0.63	0.38	0.4952	0.2971	0.0031	0.0019
G-LEAD_40_BL_U1_DIG	40	0	1.54	0.92	281.3	0.55	0.33				
G-LEAD_40_24h_U1_DIG	40	24	0.81	0.49	34.4	2.36	1.42	1.0011	0.6007	0.0236	0.0142
G-LEAD_41_BL_U1_DIG	41	0	1.41	0.85	299.0	0.47	0.28				
G-LEAD_41_24h_U1_DIG	41	24	1.25	0.75	39.9	3.14	1.88	0.0605	0.0363	0.0019	0.0011
G-LEAD_42_BL_U1_DIG	42	0	2.54	1.52	145.1	1.75	1.05				
G-LEAD_42_24h_U1_DIG	42	24	1.22	0.73	40.4	3.02	1.81	0.3009	0.1805	0.0091	0.0055
G-LEAD_43_BL_U1_DIG	43	0	1.07	0.64	186.5	0.57	0.34				
G-LEAD_43_24h_U1_DIG	43	24	0.58	0.35	41.2	1.41	0.85	2.5306	1.5184	0.0357	0.0214
G-LEAD_45_BL_U1_DIG	45	0	1.81	1.08	167.8	1.08	0.65				
G-LEAD_45_24h_U1_DIG	45	24	0.73	0.44	46.4	1.57	0.94	0.3765	0.2259	0.0059	0.0036
G-LEAD_46_BL_U1_DIG	46	0	3.44	2.07	224.7	1.53	0.92				
G-LEAD_46_24h_U1_DIG	46	24	1.65	0.99	73.4	2.25	1.35	0.9490	0.5694	0.0214	0.0128
G-LEAD_47_BL_U1_DIG	47	0	0.89	0.53	91.3	0.98	0.59				
G-LEAD_47_24h_U1_DIG	47	24	0.67	0.40	38.3	1.76	1.05	0.0399	0.0240	0.0007	0.0004
G-LEAD_48_BL_U1_DIG	48	0	1.30	0.78	284.4	0.46	0.27				
G-LEAD_48_24h_U1_DIG	48	24	0.70	0.42	52.3	1.33	0.80	0.0033	0.0020	0.0000	0.0000
G-LEAD_49_BL_U1_DIG	49	0	0.69	0.41	139.8	0.49	0.30				
G-LEAD_49_24h_U1_DIG	49	24	0.95	0.57	44.5	2.13	1.28	0.5419	0.3252	0.0116	0.0069
G-LEAD_50_BL_U1_DIG	50	0	1.82	1.09	267.2	0.68	0.41				

1	2	3	4	5	6	7	8	9	10	11#	12
**SAMPLE ID**	**SUBJECT**	**time**	**Total Pb in sample (µg/L)**	***U***	**Urine Creatinine (mg/dL) (CREA)**	**µg Pb / g CREA**	***U***	**$x(^{204}Pb)$ /%**	**$U\ (x(^{204}Pb)$**	**µg ^{204}Pb-TRACER/ g CREA**	**$U\ (x(^{204}Pb)$-norm**
G-LEAD_50_24h_U1_DIG	50	24	0.68	0.41	72.6	0.94	0.56	0.3229	0.1937	0.0030	0.0018
G-LEAD_51_BL_U1_DIG	51	0	0.55	0.33	86.4	0.63	0.38				
G-LEAD_51_24h_U1_DIG	51	24	0.81	0.49	43.3	1.88	1.13	0.0487	0.0292	0.0009	0.0005
